# Analysis of synoptic weather patterns of heatwave events

**DOI:** 10.1007/s00382-023-06828-1

**Published:** 2023-05-19

**Authors:** Sergi Ventura, Josep Ramon Miró, Juan Carlos Peña, Gara Villalba

**Affiliations:** 1 Sostenipra Research Group (SGR 01412), Institute of Environmental Science and Technology (MDM-2015-0552), Z Building, Universitat Autònoma de Barcelona (UAB), Campus UAB, 08193 Bellaterra, Barcelona Spain; 2 Department of Territory and Sustainability, Meteorological Service of Catalonia, Generalitat de Catalunya, Barcelona, Spain; 3 Department of Chemical, Biological and Environmental Engineering, Universitat Autònoma de Barcelona (UAB), Campus UAB, 08193 Bellaterra, Barcelona Spain

**Keywords:** CORDEX, ERA5 reanalysis, Multivariate analysis, Climatic trends, Heatwaves

## Abstract

Heatwaves (HWs) are expected to increase both in duration and intensity in the next decades, but little is known about their synoptic and mesoscalar behavior, which is especially important in mid-latitude regions. Most climate research has focused on temperature analysis to characterize HWs. We propose that a combination of temperature and synoptic patterns is a better way to define and understand HWs because including atmospheric circulation patterns provides information about different HW structures that can irregularly affect the territory, and illustrate this approach at the regional and urban scales using the Iberian Peninsula and the Metropolitan Area of Barcelona as case studies. We first select HW events from 1950 to 2020 and apply a multivariate analysis to identify synoptic patterns based on mean sea level pressure, geopotential height at 500 hPa, and maximum daily 2 m temperature. The results indicate that four synoptic patterns reproduce at least 50% of the variance in HWs, namely, “stationary and stable”, “dynamic and advective”, “stationary and advective”, and “dynamic, advective and undulated”. Next, we apply the analysis to the Representative Concentration Pathway future scenarios (RCPs) 4.5 and 8.5 from the Coordinated Regional Climate Downscaling Experiment (CORDEX) to determine how these synoptic trends can change in the future. The analysis shows that the four synoptic patterns continue to explain 55 to 60% of the variance in HWs. Future HW events will be characterized by an increase in geopotential height at 500 hPa due to the northward shift of the anticyclonic ridge. This is especially true for RCP8.5, which simulates business as usual incrementing fossil fuel use and additionally shows an increase in atmospheric dynamism in north advections from all directions in comparison with RCP4.5. These findings point to the importance of considering the geopotential height in HW prediction, as well as the direction of advections.

**Supplementary Information:**

The online version contains supplementary material available at 10.1007/s00382-023-06828-1.

## Introduction

According to the World Meteorological Organization, the annual mean global temperature is likely to rise between 0.9 and 1.8 °C above preindustrial levels (1850–1900) in the next 5 years (WMO 2021). This global warming is mainly attributed to the increase in greenhouse gas (GHG) concentrations in the atmosphere, which mostly (78%) results from fossil fuel burning and industrial processes (Blanco et al. 2014). The negative effects of global warming are not equally distributed but depend on geography and weather patterns (D’ippoliti et al. 2010). According to the Mediterranean Experts on Climate and Environmental Change (MedECC), the Mediterranean region is warming 20% faster than the global average (MedECC 2020). The European climate depends on mid-latitude atmospheric circulation, which is mainly controlled by westerly flows from the Atlantic Ocean (Ozturk et al. 2021). The Mediterranean climate is temperate with a dry summer season but suffers significant variability due to the transition between cold mild latitudes and the tropics, generating notorious circulation changes. Wind flow anomalies in the upper troposphere and the half-degree dip in the sea‒land temperature gradient may be the main causes of this atmospheric hotspot (Tuel and Eltahir 2020). The Third Report on Climate Change in Catalonia (Government of Catalonia 2017) predicts temperature increments ranging from + 1.1 to + 2.5 °C for 2031–2050 summers in comparison to 1971–2000, which is higher than the annual increment, which ranges from + 1.0 to + 2.2 °C in this region.

In addition to an overall temperature increase, the consequences of global warming are also expected to include an increase in the intensity and duration of heatwaves (HWs), a trend that has already been seen in recent years (Perkins-Kirkpatrick & Lewis 2020). There are multiple ways to define an HW. The World Meteorological Organization (WMO 2021) considers an HW as a five-day episode with temperatures 5 °C higher than the maximum mean of May–September calculated from the reference period (1971–2000). (Peña et al. 2015) defined an HW as a period during which the 95th percentile of summer temperatures is reached during at least three consecutive days. Such anomalous prolonged periods of excessive heat can cause wildfires and severe negative impacts on human health, agriculture and nature (de Rigo et al. 2017; McMichael & Lindgren 2011; Turco et al. 2014). For example, the HW of July 2003 in Europe culminated in 30,000 deaths (14,800 deaths in France) (Bouchama 2004).

The effects of global warming and HWs are further exacerbated in cities, which additionally suffer the urban heat island (UHI) effect due to the heat accumulation of building materials and human activity (Liu et al. 2018; Morris et al. 2017). A UHI is defined as the temperature difference between the urban center and the rural surroundings due to the property alteration of the atmospheric boundary layer (Streutker 2002; Segura et al. 2021), including turbulence (Grimmond and Oke 1999) and moisture (Hoffmann et al. 2011). During HW episodes, UHIs can raise temperatures by 0.5 °C in comparison with surrounding areas and by 2 °C at night (Basara et al. 2010), which can lead to heat stress (Guarino et al. 2014; López-Bueno et al. 2021). Furthermore, an increase in temperature can increase the demand for energy and water for cooling, which is accompanied by an increase in pollutants (Santamouris 2014). Currently, cities concentrate more than half of the world´s population, and by 2035, they are expected to hold 62.5% of the world population and 85% of the population in high-income countries (United Nations 2020). As cities plan adaptive and mitigation strategies for such events (Gilabert et al. 2021; Segura et al. 2022), a rigorous understanding of potential future scenarios of HW episodes is highly relevant and necessary (United Nations 2016).

Future predictions of HW episodes state that extreme episodes such as 2003 could occur every 15 years in the 2020–2049 period (Barriopedro et al. 2011). Furthermore, according to (Lau and Nath 2014), HWs are expected to increase in duration (by a factor of 1.4–2.0), frequency (by a factor of 2.2–4.5) and number of HW days per year (by a factor of 3–7) in Europe. To simulate and interpret future predictions of HWs, some studies have used reanalysis datasets (Bengtsson et al. 2004; Engdaw et al. 2021; Santer et al. 2004) together with climate models to statistically find climate trends focused on percentiles of temperature related to HWs. However, focusing solely on temperatures is insufficient to determine climate trends characterizing HWs; synoptic structures are also needed. The mechanisms that contribute to the formation of HWs do not occur independently of circulation conditions, and some configurations are more likely to produce extremely warm periods (Sfîcă et al. 2017).

An analysis of the behavior of synoptic weather patterns (SWPs) could improve our understanding of HWs because including atmospheric circulation patterns provides information about the structures that generate these events. This better understanding could improve HW forecasts, which could help alert authorities and decrease the negative effects of extremely high temperatures (Della-Marta et al. 2007). Previous studies have analyzed synoptic patterns to understand how extreme climate events are generated, such as wildfires (García-Ortega et al. 2011), Saharan dust intrusions (Díaz et al. 2017), and heatwaves (Sousa et al. 2019).The latter shows that, in August 2018 and June 2019, a cyclonic circulation in the northeastern Atlantic and a subtropical ridge pattern over the Iberian Peninsula advected an anomalously warm air mass which generated a HW episode. Other studies have related HW events and synoptic patterns, such as Choi et al. (2021) who found a positive linear relationship between HW events and the synoptic stagnation index which is an indicator of atmospheric stability and clear sky days. Another Asian study (An and Zuo 2021) found that regional dry heatwaves tend to happen when there is a high-pressure ridge situated to the northwest of North China and when the northern edge of the western North Pacific Subtropical high is south of 30° N. Such synoptic behavior analysis is key in understanding heatwave generation. In this study we aim to add to this knowledge by providing a long historical statistical analysis to rigorously characterize HWs using temperature and synoptic structure in a way that can be applied to future climate predictions provided by regional climate models (RCM).

To define the most representative SWPs related to HWs, we classify HW episodes based on the mean sea level pressure (MSLP) and geopotential height at 500 hPa (Z500) using principal sequence pattern analysis (PSPA) (Peña et al. 2011), which is a variant of principal component analysis (PCA) set in T-mode (correlation between fields) instead of S-mode (correlation between temporal series). PCA has the advantages of reducing and interpreting massive datasets while simultaneously minimizing information loss. This method finds the most correlated input variables that represent the highest number of total variances possible and generates new variables, which are linear functions of those in the original dataset. (Hotelling 1933; Pearson 1901) utilized the first references of this method, which was not well known until computers obtained more computational power (Jolliffe et al. 2016). More recently, PCA has been applied in different fields, such as urban traffic and meteorological data (Shiva Nagendra et al. 2003) or climate change assessment (Tadić et al. 2019).

With the overall objective of understanding HW development in terms of atmospheric structure and its effects in an urban area, we use the Metropolitan Area of Barcelona (hereafter referred to as AMB) as a case study to develop a method to classify synoptic behavior in terms of HW events. We first classify past HW events using historical ERA5 reanalysis data (1951–2020) and then analyze various simulations of future climate scenarios (2011–2100) provided by the Coordinated Regional Climate Downscaling Experiment (CORDEX) to determine the SWPs that give rise to HW episodes. We attempt to answer two questions: (1) What are the synoptic structures that give rise to HWs? (2) How well do models forecast the synoptic structures associated with HWs?

This article is organized as follows: Sect. 2 describes the area of study and data, including the datasets used for the study, the typical weather, land use and geography. Section 3 is dedicated to the methods, which include the selection of HW days, the PSPA, and the creation of mean maximum temperature at 2 m (TMAX) maps in relation to SWP trends. Section 4 applies the historic analysis to future climate scenarios to analyze possible trends in the atmospheric structure. Finally, Sect. 5 presents a summary and conclusions.

## Area of study and data

### Area of study

**Fig. 1:**
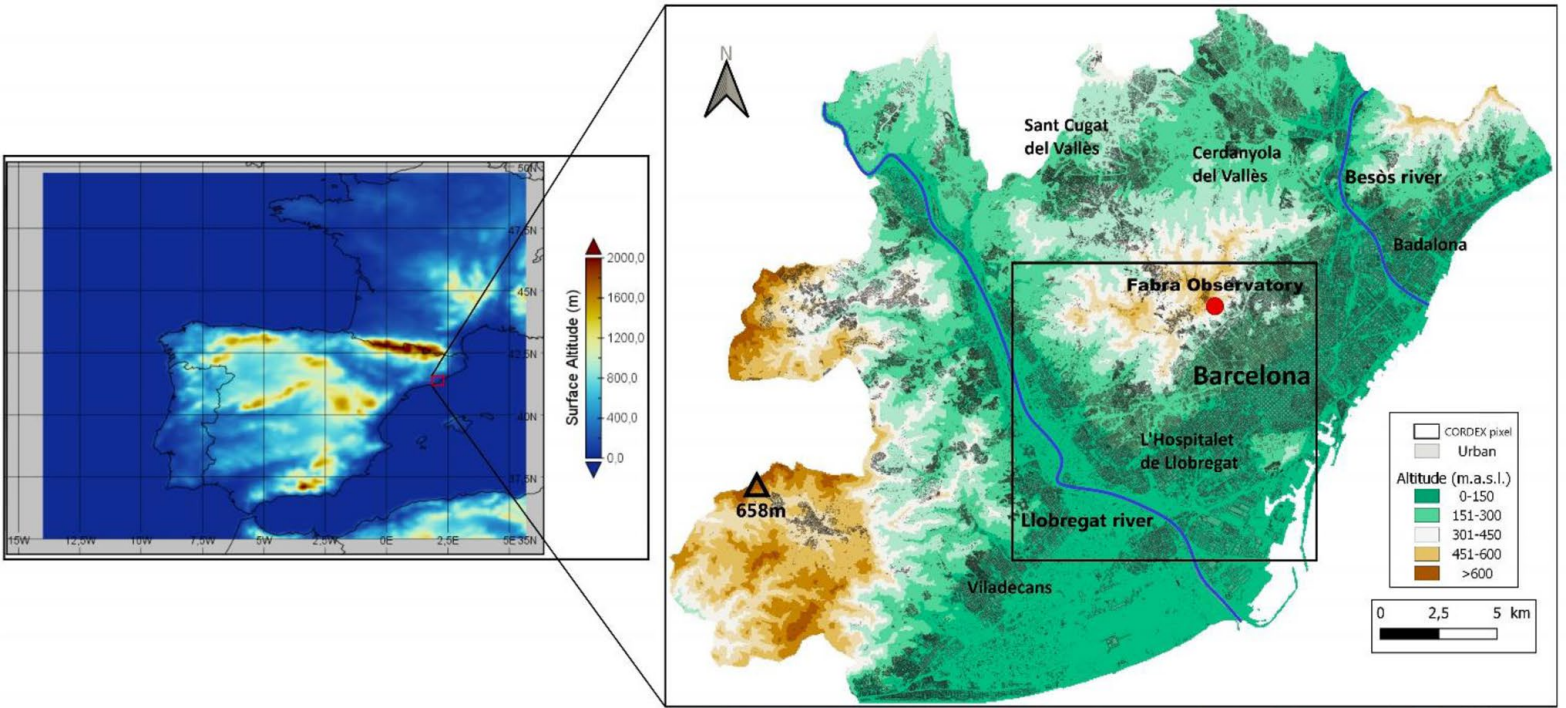
On the left, the selected area from ERA5 and CORDEX simulations, showing the surface altitude. The Metropolitan Area of Barcelona (AMB) is marked in red. On the right, the topography of the AMB, including altitude, urban fraction, the names of several municipalities and two main rivers, as well as the location of the CORDEX pixel, coinciding with the meteorological station of Fabra Observatory, used for extracting the 95th percentile of the CORDEX datasets

The region of interest is the AMB, located in the northeastern Iberian Peninsula (41–42° N/1.5–2.5° E), as shown in Fig. 1.

The climate is Mediterranean, characterized by warm and dry summers influenced by sea breezes that regulate temperatures. According to the Fabra Weather Station (Fabra for short), the mean maximum and minimum temperatures in summer months (June, July and August: hereafter referred to as JJA) are 27.7 °C and 18.8 °C, respectively, with a mean summer precipitation of 96 mm (15.3% of annual total, which is 625 mm). HWs in Barcelona can reach values of + 39 °C and are exacerbated by the high values of relative humidity generated by the Mediterranean Sea (summer mean of 71%), causing a situation of thermal stress for the inhabitants of Barcelona.

The topography of the area is heterogeneous, with a coastal mountain range 10 km from the sea reaching + 650 m.a.s.l. (meters above sea level), two important rivers, and the delta of the Llobregat River, which covers 98 km^2^. This region covers an area of 636 km^2^ and has a population of more than 3,300,000 inhabitants. From this area, 48% is urbanized, while the rest is occupied by more than 250 km^2^ of green area.

### Data

In this study, we used hourly observational data of 2 m temperature available from the weather station of the Fabra Observatory (41° 25′ 06″ N, 2° 07′ 27″ E, 415 m.a.s.l.) from 1951 to 2020. This database can be downloaded at https://apidocs.meteocat.gencat.cat/. We filtered this data selecting the summer months (JJA) and it was used to find the HW days. The HW episodes are selected based on the definition of the Catalan Meteorological Service (SMC) adapted by (Peña et al. 2015), in which an HW is an episode of three or more consecutive days that reaches the 95th percentile of the maximum summer temperatures. In addition to the observational data from the SMC, we used the Spain02 dataset providing daily temperature and precipitation from 1951 to 2020 and covering Spain. This dataset is downloaded in a regular grid of 5 km from Spain02 interpolated products (data source: https://www.aemet.es/es/serviciosclimaticos). Mean maximum temperature maps were used to support the results.

Reanalysis gridded data at a 0.25° resolution from ERA5 was used for the historical period (1951–2020) analysis. This data was necessary for the methods described in Sects. 3.1 and 3.2 and was downloaded from the Copernicus Climate Data Store available at https://cds.climate.copernicus.eu/cdsapp#!/home. The region used for this analysis covers from 35° N, 14° W to 50° N, 6° E, as represented in Fig. 1.

EURO-CORDEX data was used for the whole period (1951–2100), considering two representative concentration pathways (RCPs) 4.5 and 8.5. The domain of the EURO-CORDEX simulations includes the entire European continent, from North Africa and the Atlantic in the south and west, to the west of Russia and Turkey in the south and east (official source: https://cordex.org/domains/cordex-region-euro-cordex/). The left hand panel of the Fig. 1 shows the selected area for the statistical analysis of the synoptic weather patterns over the Iberian Peninsula and the AMB. The simulations of the historical period and both scenarios are available from Copernicus (https://cds.climate.copernicus.eu/cdsapp#!/dataset/projections-cordex-domains-single-levels?tab=form). The RCPs are pollutant concentration pathways used in the Fifth Assessment Report of the Intergovernmental Panel on Climate Change (IPCC). The values of the RCPs refer to the radiative forcing that would occur due to the increase in anthropogenic emissions by 2100 (IPCC 2014). Thus, RCP4.5 is described as the most likely scenario, with greenhouse gas emissions peaking in 2040 followed by a decline. RCP8.5 is the worst-case scenario in which emissions continue to rise throughout the twenty-first century. In this work, three RCM have been used (Table 1) at 0.1° resolution to perform the analysis.

**Table 1:** Description of the CORDEX data used in this study

CMIP5 GCM	Resolution GCM (º)	EURO-CORDEX RCM	Resolution RCM (^o^)	Institution (country)
IPSL-CM5A-LR	**1.9 × 3.75**	**WRF381P**	0.11	IPSL (France)
MPI-M-MPI-ESM-LR	**1.9 × 1.9**	**REMO2009**	0.11	MPI-CSC (Germany)
MOHC-HadGEM2-ES	**1.9 × 1.25**	**HIRHAM5**	0.11	DMI (Denmark)

## Methods

**Fig. 2:**
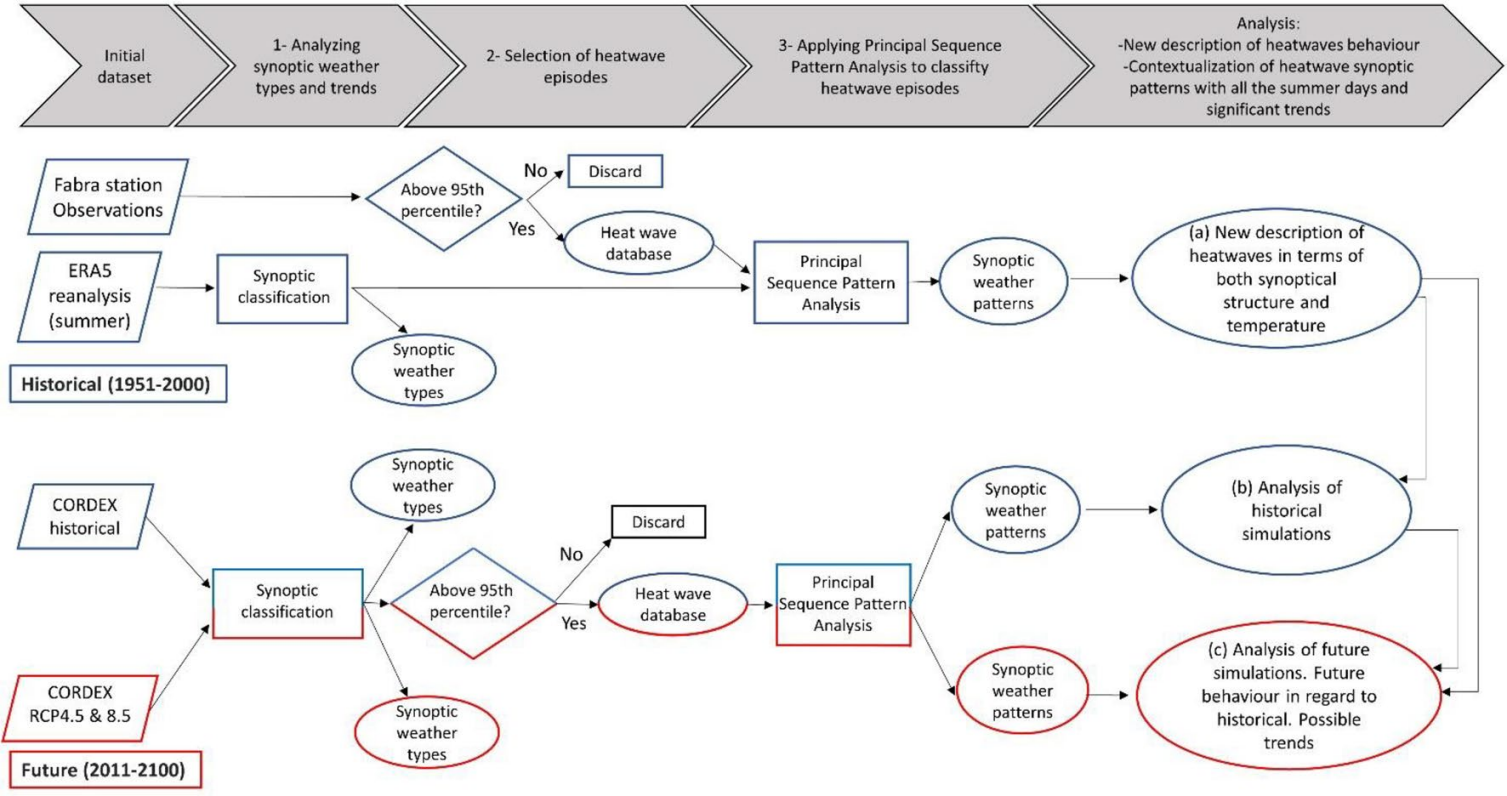
Graphical workflow: Steps 1, 2, and 3 are defined at the top of the image. Rhomboids represent publicly available datasets needed to apply methods (rectangles) at each step. Triangles represent questions, and ovals include the dataset or analysis resulting from each step. The upper section (in blue) corresponds to the historical analysis based on 1951 through 2000, whereas in parallel but below and in red, the methods are applied to future (2011–2100) simulations obtained from CORDEX for future climate scenarios RCP 4.5 and 8.5

The methods employed to classify HWs into synoptic and atmospheric structures consist of the three steps outlined in Fig. 2: (1) the classification of all the JJA days by synoptic weather types; (2) the extraction of HW days from observed data using the 95th percentile of maximum temperatures in the summer months; and (3) the application of the statistical PSPA to the HW days extracted in the previous step to reduce the dataset, improve the interpretability and find the SWP associated with HWs. In the following paragraphs, we describe each of these steps and the datasets used (all publicly available) in further detail.

### Analyzing synoptic weather types and trends

The JJA days from 1951 to 2020 are classified according to an objective synoptic classification appropriate in Mediterranean climates as described by (Miró et al. 2020) and shown in Table 2. This method is based on the classic Jenkinson–Collison classification (Jenkinson and Collison 1977) but with an additional geopotential level of Z500 to account for the important effect of mid-troposphere mechanisms in Mediterranean weather (Tuel and Eltahir 2020; Ward 1963), resulting in 13 synoptic weather types for the northeastern Iberian Peninsula.

**Table 2:** Synoptic weather types with a brief description as defined by (Jenkinson & Collison 1977) and the frequency of HW events during the period 1951–2020 in the AMB

Synoptic weather type	Description	% Frequency (1951–2020)
Type01	West advection	0
Type02	Anticyclonic western advection	11.6
Type03	Northwest advection	0.5
Type04	North advection	1.1
Type05	Northeast advection	2.1
Type06	East advection	2.1
Type07	East advection with cutoff low above	2.7
Type08	South advection	0
Type09	Southwest advection	5.8
Type10	Trough	9
Type11	Low or cyclone	13.2
Type12	Shallow cyclone or undetermined pressure gradient	49.7
Type13	Anticyclone	2.1

We used the Mann–Kendall test (Mann 1945; Kendall 1975) to classify all summer days between 1951 and 2000 into the 13 synoptic weather types. This technique has been widely used for detecting trends in hydrometeorological series (Liu et al. 2013; Zhang et al. 2000). It is a nonparametric method that is not influenced by extreme values due to its robustness to outliers in time series data. The presence of a monotonic trend in the series has been estimated using the tau statistic at the 95% level (p value < 0.05). The Mann–Kendall test checks the null hypothesis, which indicates that there is no significant trend (p value > 0.05). If this cannot be verified (p value < 0.05), the alternative hypothesis is accepted, which indicates an increasing or decreasing trend in the time series data. In this work, the software provided by (Hussain and Mahmud 2019) is applied to generate the Mann–Kendall trends from the synoptic weather types. If the output value has a significant trend, the Tau–Kendall value is used to check if it is an increasing (τ > 0) or a descending trend (τ < 0). However, studies that consider a set of statistical inferences simultaneously could face the problem of multiple testing (Streiner and Geoffrey 2011). For this, we adjusted the p value using the Holm-Bonferroni sequential correction (Holm 1979), which has been proved with better results than the Bonferroni procedure (Aickin and Gensler 1996). In this paper, we calculated the adjusted p values using a Microsoft Excel  online calculator developed by (Gaetano 2013).

The results of these methods can be categorized into four possibilities: a nonsignificant increasing trend (NSIT), which shows an increasing trend that is not statistically significant; a nonsignificant decreasing trend (NSDT), which shows a decreasing trend that is not statistically significant; a significant increasing trend (SIT), which is statistically significant with a trend to increase; and a significant decreasing trend (SDT), which is statistically significant and has a trend to decrease.

### Selection of heat wave episodes

The HW episodes can be selected following multiple definitions, since there is not a universal definition for it. In this article, we used the definition of the SMC (Sect. 2.2), which has been elaborated for this region of study. The 95th percentile has been calculated for each climatic period of 30 years separately and, consequently, has been changing over time. The output of this step is a database of all the HW episodes registered in each climatic period.

### Applying principal sequence pattern analysis to classify heat wave episodes

We refer to this third step of the methods as the PSPA. This process was applied for all days previously classified as HW days to obtain a synoptic classification related to these events, following the definition from 2.2. For this, we generated a database of HW episodes divided into five climatic periods of 30 years each (1951–1980, 1961–1990, 1971–2000, 1981–2010, 1991–2020), following the climatological standard normal (WMO 2016). The PSPA was carried out using gridded data at a 0.25° resolution from ERA5 reanalysis (see Sect. 2.2.).

Analyses of the synoptic history can be undertaken using a multivariable classification of the synoptic sequences related to the main atmospheric parameters, with an hourly or daily resolution. Methodologically, this classification is supported as a variant of PCA and is known as PSPA (Aran et al. 2011; Compagnucci et al. 2001; Escobar et al. 2004; Esteban 2008; Jacobeit et al. 2006; Peña et al. 2015; Philipp 2009). The analysis integrates different atmospheric levels (MSLP and Z500) with the purpose of understanding the main features that account for the dynamic atmospheric processes. The need for this has been recognized in previous studies (Houssos et al. 2008; Peña et al. 2015; Sioutas and Flocas 2003), which mention the interest of using this information to build climatological classifications and implement them into forecast systems.

We applied the PSPA in T-Mode to the HW dataset resulting from the previous step using a correlation matrix (Escobar et al. 2004; Huth et al. 2008), a scree test to extract the most relevant components (Cattell 1966) and orthogonal Varimax rotation to satisfy the orthogonality condition of the model (Richman 1986). More details about the data matrix can be found in the supplementary material S1. The analysis was conducted for the domain from 30° N to 70° N and from 30° W to 30° E for the period 1951–2020. As a result, the output of the PSPA process is a set of SWPs (both from MSLP and Z500) that describe the main patterns associated with HW days. We have developed a Python script to run this and have made it publicly available through GitHub [https://github.com/URBAG-ICTA/PSPA_HW.git].

### Analysis of CORDEX historical HW episodes

One of the main objectives of this study is to analyze how future climate change simulations predict HWs in terms of the synoptic structure. However, we first need to analyze how the climate models perform in representing synoptic behavior at the regional level. The objective of this section is to analyze the synoptic structures of the simulations available from CORDEX for the period 1951 to 2000 and determine if they are well represented when compared to reanalyzed ERA5 data.

Quantile‒quantile mapping transformation (Q–Q, Amengual et al. 2012) is used to normalize the model simulation grid. This is done to align the statistical distribution of ERA5 reanalysis and CORDEX datasets, which removes technical variation from noisy data. In this work, we have applied this method for all three variables used, MSLP, Z500 and T2M. This procedure consists of calculating the changes, quantile by quantile, in the cumulative distribution frequency (CDF) of the daily MSLP and Z500 outputs and the observed data. The statistical adjustment is based on the relationship between the ranked value of the corresponding CDFs for past calibrations (1951–2000), the control instrumental or baseline (1971–2000), and the raw control simulated by CORDEX models (1951–2000). The Q–Q method is applied to the CORDEX data from simulations obtained from three different regional climate models: WRF, REMO, and HIRHAM for the historical period 1951–2000 (Table 1). Once the model simulation outputs for MSLP and Z500 are corrected, steps 3.1, 3.2 and 3.3 are repeated for the period 1951 through 2000 to obtain an analysis of historical simulations (see Fig. 2).

### Analysis of CORDEX future scenarios of HW episodes

The same three steps 3.1, 3.2 and 3.3 are repeated to determine the synoptic behavior of the HW events occurring in two future scenarios for the period 2011 through 2100: RCPs 4.5 and 8.5. Simulations of these scenarios using WRF, HIRHAM and REMO are available from CORDEX, which details can be found at Table 1. The result of this step is a set of SWPs, as shown in Fig. 2.

## Results

### Analyzing synoptic weather types and trends

In this section, we classified all the summer days to different weather types following the objective classification by (Miró et al. 2020) and, next, we applied the Mann–Kendall test to find the significant trends. This analysis for the historical reanalysis data (1951–2020) shows that synoptic weather types 11 (low or cyclone) and 12 (shallow cyclone or undetermined pressure gradient) are the most frequent during the summer months, dominating up to 18% and 40% of the time, respectively, followed by types 2 (anticyclonic western advection), 5 (northeast advection) and 10 (trough), as shown in Table 3. This means that summers are mostly characterized by undetermined pressure gradients (type 12), which are periods without any dominant high or low pressures. This pattern is commonly defined by a stationary high-pressure center located over the Azores that maintains low pressures in northern regions, blocking any possible weather front. Low pressures (type 11) can be defined by two different situations. In the first case, low pressure situations are structures with pressure levels lower than 1013 hPa, strong cyclonic winds and structures that generate weather fronts. However, in HW situations, the most common structure in the Iberian Peninsula is a thermal low resulting from the heating of the lower troposphere, generating weak cyclonic circulations. Table 3 also shows the trend determined by the Mann–Kendall method.

**Table 3:** Percentage of times that synoptic events occur for every climatic period for JJA

	1951–1980	1961–1990	1971–2000	1981–2010*	1991–2020*	Tau–Kendall	p value **	Trend
TYPE01	1.38	1.12	1.30	1.52	1.41	− 0.05	0.59	NSDT
TYPE02	9.64	9.71	9.75	11.09	11.12	0.05	0.58	NSIT
TYPE03	2.75	2.36	2.54	2.14	2.50	0.06	0.51	NSIT
TYPE04	6.20	5.14	4.93	4.35	4.93	− 0.16	0.07	NSDT
TYPE05	7.97	8.19	8.41	8.22	8.70	0.12	0.16	NSIT
TYPE06	1.92	2.17	2.36	2.57	2.10	− 0.05	0.56	NSDT
TYPE07	2.50	2.21	1.67	1.67	1.56	**− 0.18**	**0.05**	**SDT**
TYPE08	0.04	0.11	0.07	0.11	0.04	0.03	0.81	NSIT
TYPE09	3.91	3.84	4.38	3.70	3.19	− 0.11	0.20	NSDT
TYPE10	7.90	7.68	8.19	7.75	7.14	− 0.07	0.44	NSDT
TYPE11	18.12	18.55	18.55	17.10	16.34	− 0.09	0.27	NSDT
TYPE12	36.78	38.01	36.96	39.02	40.04	0.15	0.07	NSIT
TYPE13	0.90	0.91	0.91	0.76	0.94	− 0.05	0.61	NSDT

Mann–Kendall (Tau–Kendall, p value and trend

NSDT nonsignificant decreasing trend, NSIT nonsignificant increasing trend, SDT significant decreasing trend, SIT significant increasing trend)

*2003 not considered

**p value adjusted using the Holm–Bonferroni method

Looking at the right of Table 3, only one significant trend exists in the sample, which is the decreasing trend of type 07. Although type 07 has a frequency of 2.5% in the first climatic period, its trend is to decrease, and in the last climatic period, it has a frequency of 1.56%.

There are two synoptic weather trends that are close to having a significant trend according to the criterion used in this work (p value ≈ 0.05), which are types 04 and 12.Type 12 (which is a potential HW pattern according to the synoptic classification) has an increase from 36.78% to 40.04%, and it is close to an increasing trend (p value = 0.07).

### Selection of heat wave episodes

**Fig. 3:**
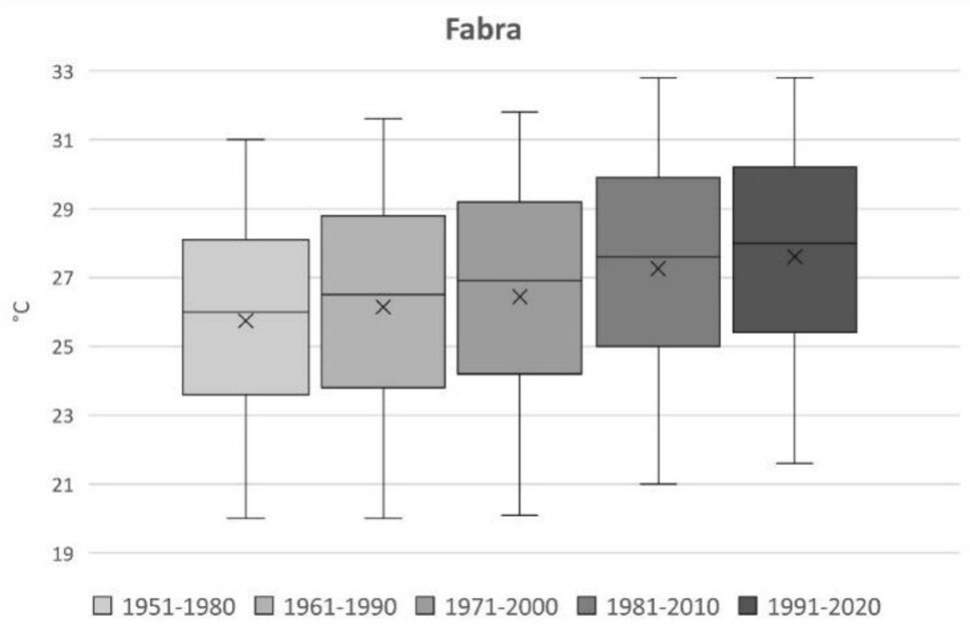
Boxplot from observations for the 1951–2020 JJA period. The boxes are defined by 25–75th quantiles, and whiskers are defined by the 5–95th quantiles. The 95th percentile is quantified (°C)

A rising trend in temperatures from 1951 until 2020 can be noted in Fig. 3, where each boxplot shows the mean, median and the 5th, 25th, 75th and 95th percentiles of maximum summer temperatures for the historic five time periods using observed data from the Fabra weather station. Due to their insignificance in the scope of the study, outliers were not included in the preparation of this figure. From these boxplots, the 95th percentile is extracted to select the episodes of three or more days according to the definition of HW adopted in this study. The result is a database of 139 HW days ranging from 31.9 to 39.8 °C. According to these results, the Fabra weather station registered an increment of 1.8 °C for HW episodes between the first and the last climatic period under study.

### Applying principal sequence pattern analysis to classify HW episodes.

The PSPA method is applied for all HW days previously identified to obtain a synoptic classification related to each HW event. The results show that four SWPs are responsible for approximately 50% of the variance in MSLP and Z500 for all historic time periods, as shown in Table 3. These four SWPs (named SWP1, SWP2, SWP3, and SWP4) are characterized by mean matrices of multiple HW days that are constructed from linear combinations of the initial variables. The four resultant SWPs are sorted by the amount of explained variance, in which SWP1 always explains more variance than SWP2 and so forth. In this study, the explained variance refers to the statistical measure that explains the variation in a dataset attributable to each of the SWPs.

**Fig. 4:**
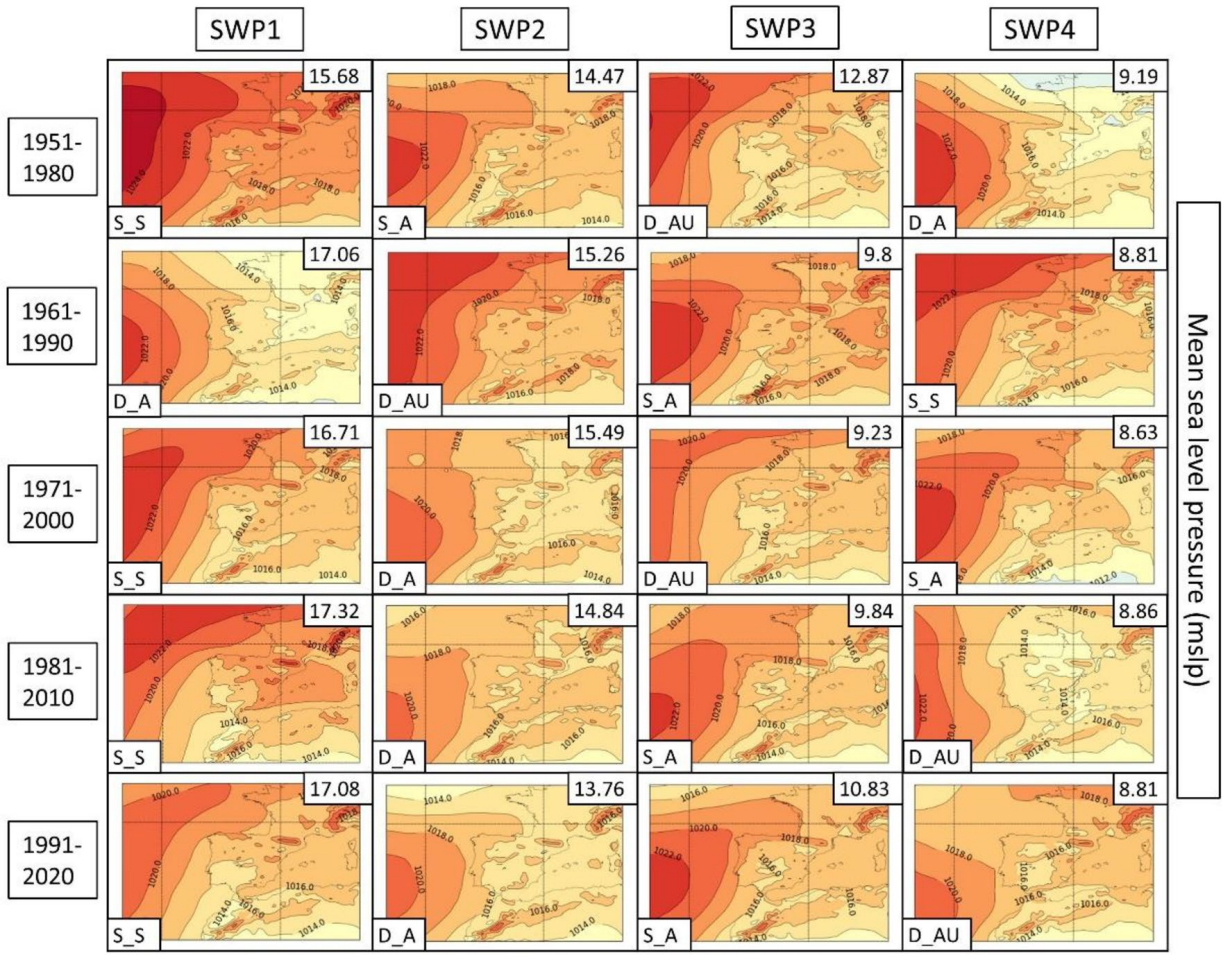
Mean sea level pressure (MSLP) variable (in hPa) for each SWP for the historical period 1951–2020. The variance % is given in the upper-right corner and the pattern in the bottom-left. Four patterns are found: S_S (stationary and stable), D_A (dynamic and advective), S_A (stationary and advective) and D_AU (dynamic, advective and undulated)

In the next few paragraphs, we give a description of each SWP for each climatic period, which can be found in Fig. 4. The amount of variance can be found in the upper right of each SWP and the short-name description in the bottom left. This explained variance ratio is a metric commonly used to evaluate the utility of SWPs and to choose the number of patterns considered (Jolliffe 2016). For the results, we have divided the four SWPs into four differentiated structures that are repeated in each climatic period. The different structures take into account both MSLP and Z500 variables, locating the mean elements such as low- and high-pressure centers, thermal lows, distance of the isolines or the distribution of ridges and troughs. Dynamism (main meteorological structures that change over time) and stationarity (stationary patterns that can remain permanent for multiple days) are also considered and discussed in the results.

**Fig. 5:**
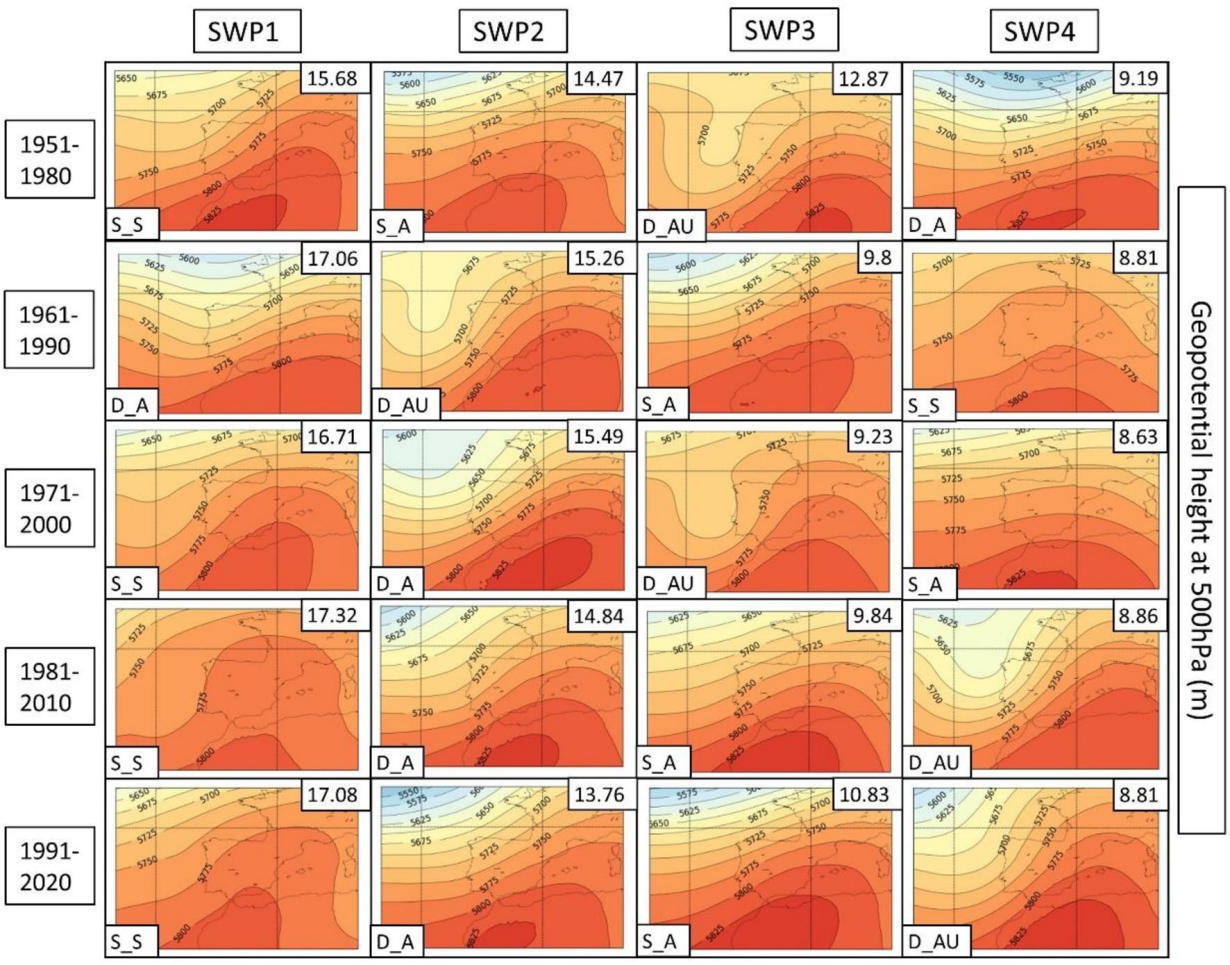
SWP for the ERA5 historical period. 500 hPa geopotential height (Z500) variable (in meters) plotted for 1951–2020. The variance is explained in the upper-right corner and the pattern in the bottom-left. Four patterns are found: S_S (stationary and stable), D_A (dynamic and advective), S_A (stationary and advective) and D_AU (dynamic, advective and undulated)

**SWP-S_S: stationary and stable pattern**: MSLP (Fig. 4) shows a thermal low and a blocking anticyclone over the Atlantic Ocean. There is a trend in surface level to an increase in the dominance of the thermal low from the north of Africa and the south of the Iberian Peninsula. The Z500 level (Fig. 5) provides more information about the evolution to a more stationary anticyclonic ridge due to the jet stream moving north. SWP-S_S is the first component in four out of five periods. In the 1961–1990 period is SWP2 (15.3% of the total explained variance). There is a trend toward an increase in the variance of this pattern from 15.7% in the first period to 17.1% in the last period.

**SWP-S_A: stationary and advective**: The MSLP variable indicates a stronger high-pressure center in the western Iberian Peninsula in comparison with SWP-D_A and a thermal low in northern Africa and the Iberian Peninsula. Z500 indicates a SW flux due to the ridge in the S and the trough in the NW of the Iberian Peninsula. The variance of this pattern decreases from 14.4 to 10.8%. SWP-S_A is the second component in 1951–1980 (14.4% of the variance), the fourth component during the climatic period 1971–2000 (8.6% of variance), and the third component in the rest of the climatic periods (9.8%, 9.8%, and 10.8% of the variance, respectively).

**SWP-D_AU: dynamic, advective and undulated**: MSLP indicates an area without the influence of high or low pressures with warm air masses that induce a thermal low in the Iberian Peninsula and northern Africa. There is a trend toward a decrease in high-pressure dominance in the NW. In Z500, there is an important undulation of the general circulation that advects winds from the south. There is a reduction trend for this pattern from 12.9 to 8.8%. SWP-D_AU is the third component in the first and third climatic periods (12.9% and 9.2% of the variance, respectively), the second component during the 1961–1990 period (15.3% of the variance), and the fourth component in the last two climatic periods (≈ 8% of the variance).

**SWP-D_A: dynamic and advective**: MSLP shows a thermal low in the Mediterranean area due to the warm sea. The high pressures tend to lose importance due to the dominance of the thermal low. The Z500 variable shows SW flux, which is getting more undulations from the mainstream in comparison with the first climatic periods. Cold air is restricted in the Atlantic Ocean. This pattern, which is becoming more advective in height, has increased in frequency from 9.19 to 13.76%. SWP-D_A is the fourth component in the first climatic period (9.2%% of explained variance), the first component during the 1961–1990 period (17.1% of variance), and the third component in the rest of the climatic periods (15.5%, 14.9%, and 13.8% of the variance, respectively).

**Fig. 6:**
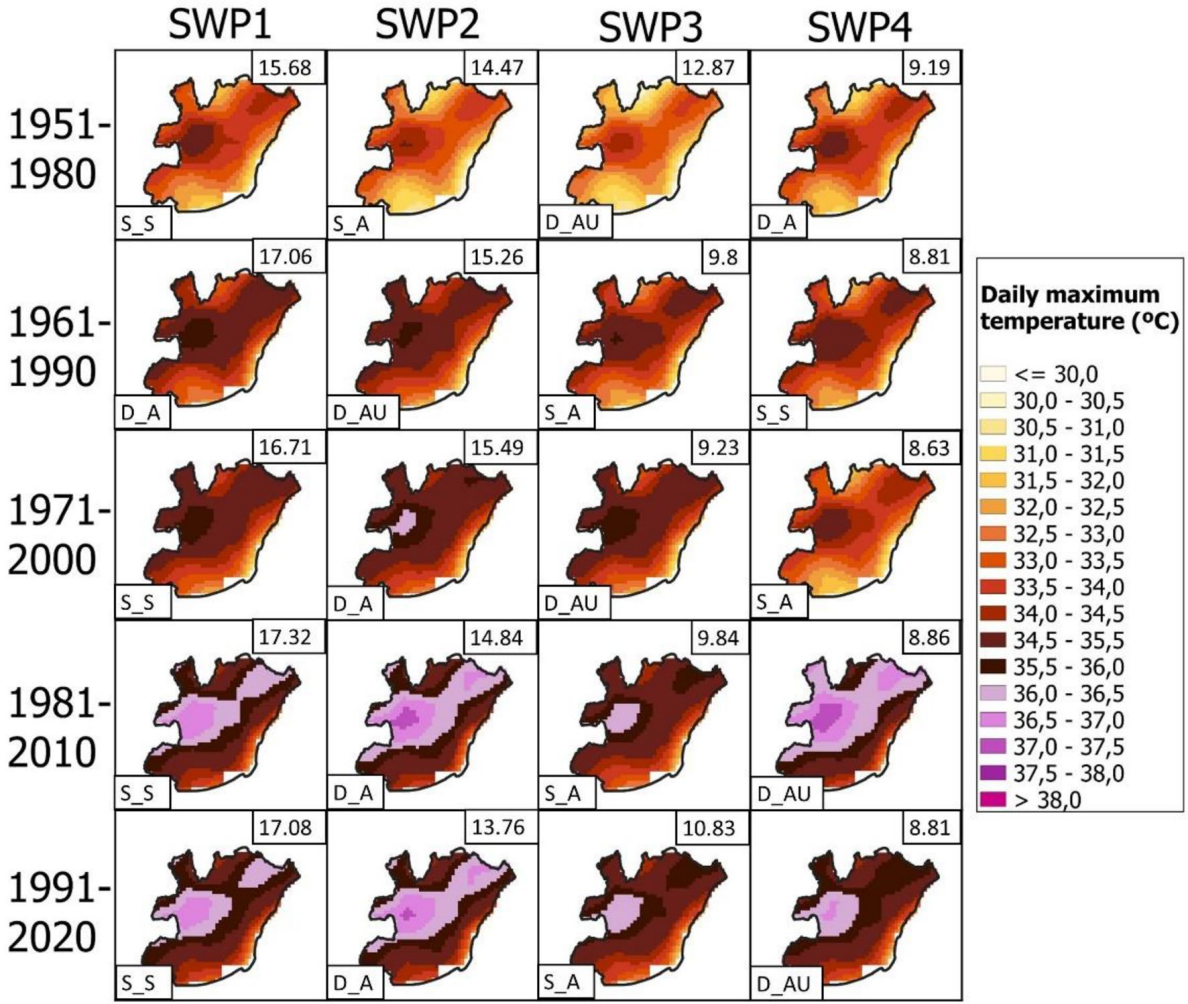
Mean maximum temperature maps (in °C) for the Metropolitan Area of Barcelona associated with SWPs by climatic period: **a** 1951–1980, **b** 1961–1990 and **c** 1971–2000. Marked: Fabra Meteorological Station

Once the HW events have been classified into the four SWPs for the Iberian Peninsula, we next analyze the effect of these four SWPs on the intensity of HWs in the Metropolitan Area of Barcelona. We use a dataset providing daily temperature and precipitation from 1951 to 2020 in a regular grid of 5 km from Spain02 interpolated products (data source: https://www.aemet.es/es/serviciosclimaticos) to generate Fig. 6: a composite temperature map showing the mean of the daily maximum temperatures corresponding to each SWP. Please see Supplementary Materials S2 and S3 for the tables with temperature values and S4 for the mean map covering all of Spain.

In the AMB, the warmest HW patterns are SWP-D_A and SWP-D_AU, which are dynamic and advective. Specifically, SWP-D_A of the 1981–2010 period is the warmest (35.1 °C, mean daily maximum temperature in the AMB), followed by SWP-S_A in 1981–2010 (35.0 °C) and SWP-S_A in the 1991–2020 period (34.9 °C). According to Fig. 6, prefrontal patterns are the warmest (D_AU) in the AMB, advecting air from the S‒SW at 500 hPa. Moreover, SWP-D_A is the pattern with a thermal low over the Mediterranean, indicating a warm sea influencing the AMB temperatures. Intense ridges at 500 hPa with a thermal low at the surface (SWP-S_S) also generate warm conditions (34.6 °C in the last climatic period) in the AMB but especially in the rest of the Iberian Peninsula. Maximum temperatures have increased by approximately 2 °C between the first and last climatic periods analyzed.

HWs registered in the AMB are mostly related to HW episodes in most of the Iberian Peninsula (see Fig. S4 in the Supplementary Material). The only SWP that differs from the rest is SWP-D_AU, in which a cold front generates lower temperatures in the NW of the peninsula.

### CORDEX historical

**Fig. 7:**
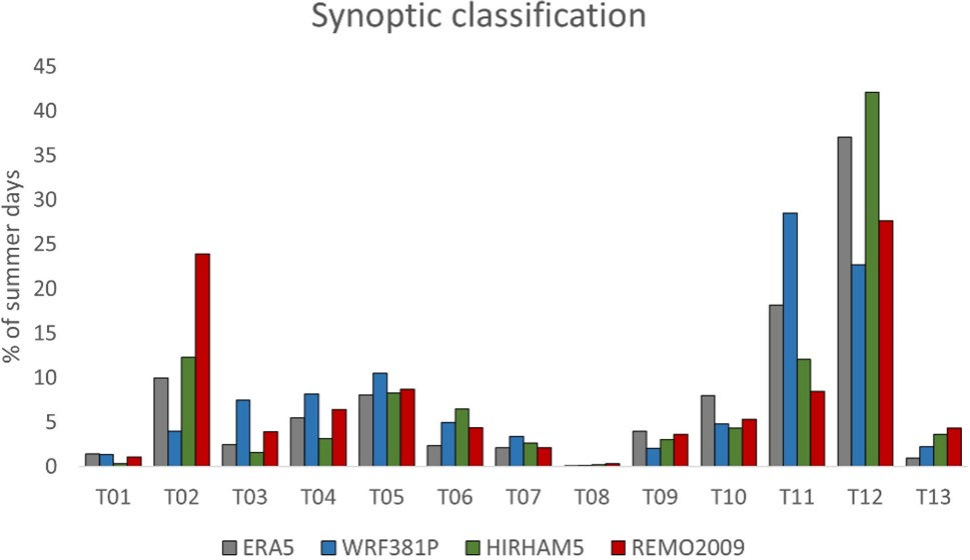
Synoptic weather types of both reanalysis (ERA5) and historical simulations (CORDEX) for the period 1951–2000. Only JJA days were selected

The same analysis that was done for historical observed data (steps 1–3 of the methods section) is next repeated for the same historical period 1951–2000 JJA but with modeled CORDEX (WRF, HIRAM and REMO) data to analyze the performance of the various models. Since one of the main research objectives is to understand future HW events in terms of the SWPs in which they develop, it is important to first establish how well historical simulations of synoptic weather types are generated. First, all JJA days for the period of 1951–2000 from the output of the WRF, HIRHAM, and REMO models were classified into 13 synoptic weather types following the method described in Sect. 3.1, as shown in Fig. 7. Most JJA days of both ERA5 reanalysis and CORDEX simulations fall into synoptic weather type 12 “shallow cyclone or undetermined pressure gradient”. HIRHAM estimates more JJA days in type 12 than ERA5 (+ 5.05%), while REMO and WRF underestimate type 12 days (− 9.45% and − 14.39%, respectively).

The main differences among model outputs can be found in types 02, 11 and 12. REMO simulations show more dynamism at 500 hPa (due to advections from different directions) and stability at the surface level (high pressures), which generates weather types 02 and 12. HIRHAM has the most similar general structure in comparison with ERA5, with a similar percentage of cases in all the most repeated patterns (02, 11 and 12). WRF has a significant increase in synoptic weather type 11, which is related to low pressures and thermal lows.

The results of the Mann–Kendall test are shown in Table S5: the CORDEX models do not simulate any significant trend in the 1951–2000 period, which is consistent with the ERA5 reanalysis. Analyzing the most frequent synoptic weather type (type 12), WRF and REMO forecast a nonsignificant decrease in cases, while HIRHAM forecast a nonsignificant increase.

Next, we analyze the differences among the HW days of the various models and compare them with the HW days from the observed data and the ERA5 reanalysis data. Boxplots with percentiles of temperature are generated to study the variability of every climatic period, shown in the supplementary material S6. The 95th percentile, represented as the top of the whisker, is the HW temperature according to the definition used in this study.

The 95th percentile of Fabra recorded an increase of + 0.81 °C between 1951–1980 and 1971–2000, while ERA5 reflected an increase of + 0.55 °C. CORDEX datasets register increases that range between 0.18 °C (HIRHAM), 0.29 °C (WRF) and 0.32 °C (REMO). The adjustment of the CORDEX dataset corrects the overestimated values that all three models have, resulting in three datasets with similar percentiles in comparison with ERA5.

**Fig. 8:**
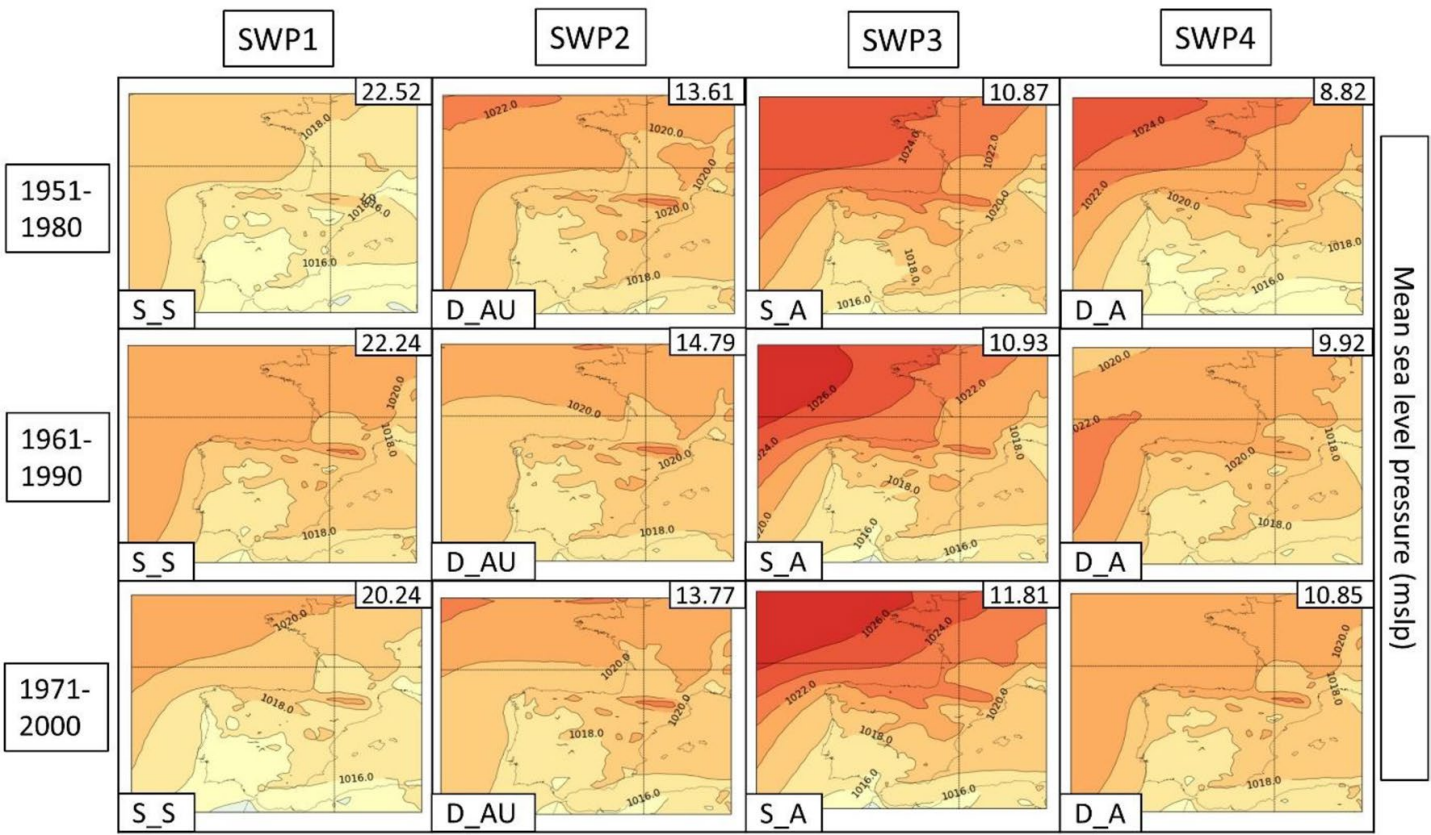
SWPs for the CORDEX historical period. Mean sea level pressure (MSLP) variable (in hPa) plotted by the climatic periods divided by files. The columns represent the SWPs with the variance explained in the upper-right corner and the synoptic pattern in the bottom-left corner. Model: WRF

**Fig. 9:**
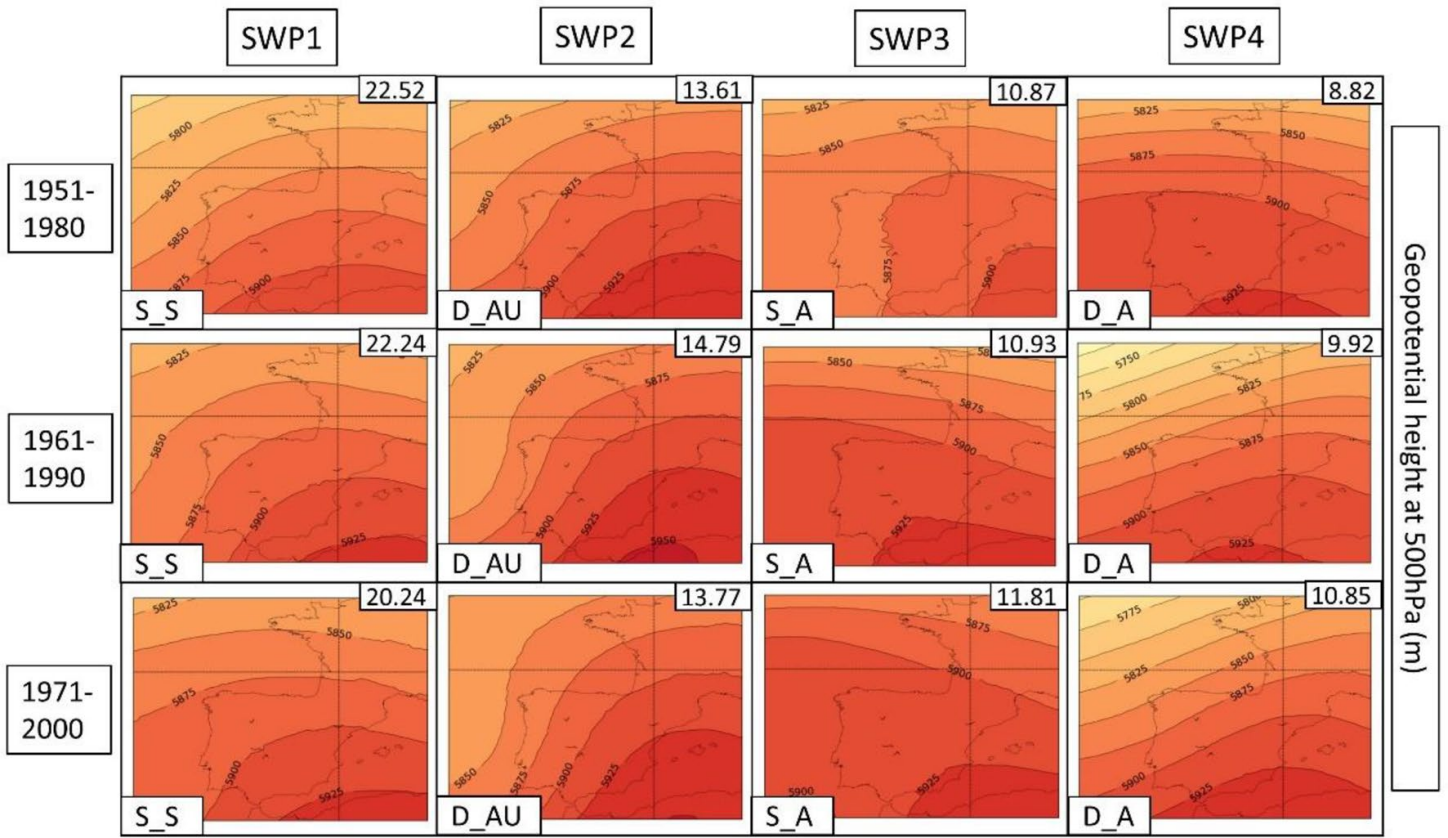
SWPs for the CORDEX historical period. Geopotential height at 500 hPa (Z5000) variable (in m) plotted by the climatic periods divided by files. The columns represent the SWPs with the variance explained in the upper-right corner and the synoptic pattern in the bottom-left corner. Model: WRF

**Fig. 10:**
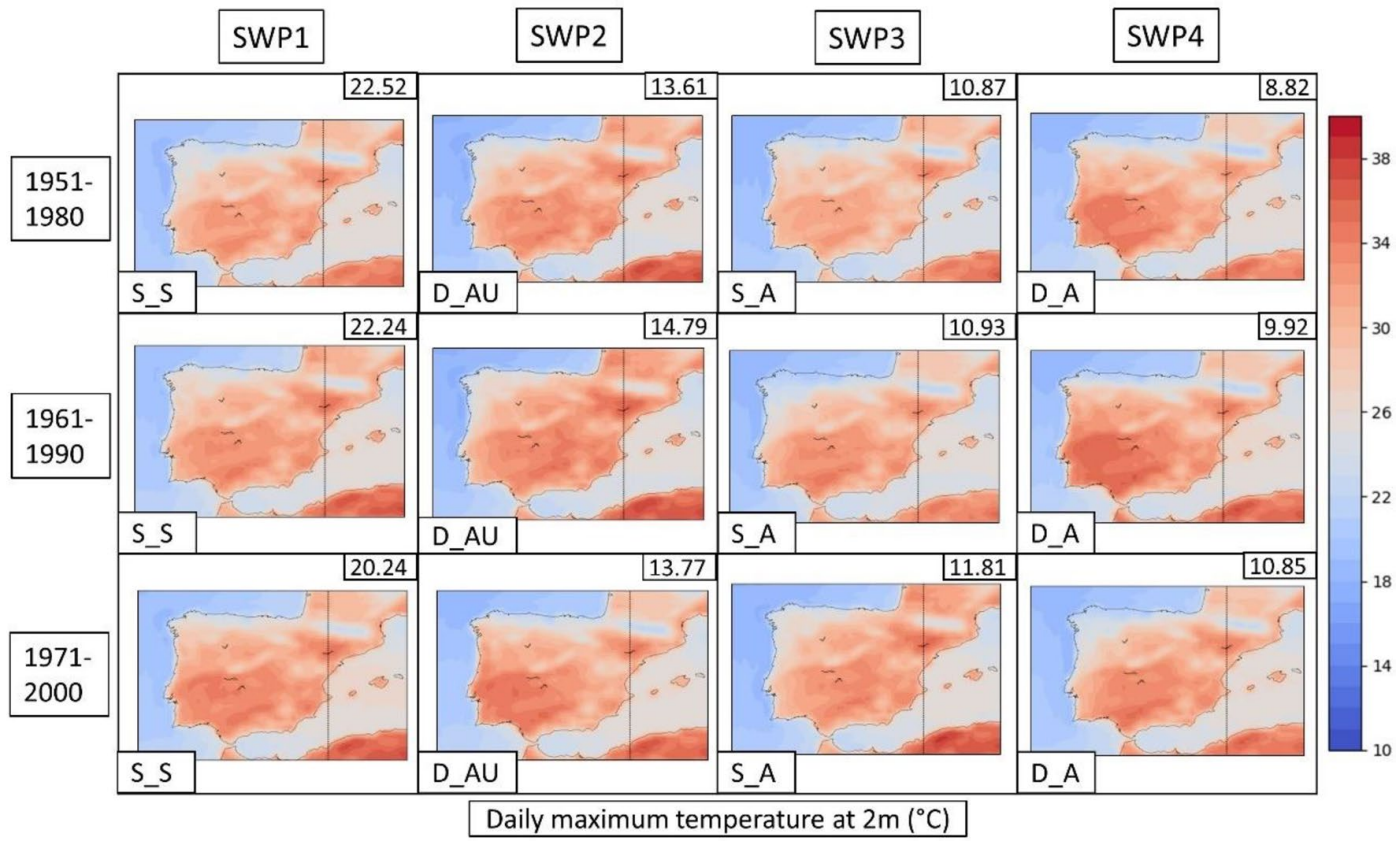
SWPs for the CORDEX historical period. Daily maximum temperature at 2 m (TMAX) variable (in °C) plotted by the climatic periods divided by files. The columns represent the SWPs with the variance explained in the upper-right corner and the synoptic pattern in the bottom-left corner. Model: WRF

The PSPA (3.3 of methods section) for the three CORDEX historical datasets reaches more than 50% of the variance in four SWPs (variance represented in the upper-right of the SWPs in Figs. 8, 9, 10). In this section, we show and describe the results of the WRF model due to the better representation of SWPs in comparison with ERA5. The description for all the CORDEX models is in Supplementary Material S7 and REMO-HIRHAM plots in S8–S9.

The criteria used to select the model that best replicates the SWPs found in ERA5 data have considered multiple factors. WRF is the model with less overestimation for the SWP1 (SWP-S_S) variance, according to Figs. 8 (MSLP), 9 (Z500) and 10 (TMAX). WRF shows a variance that ranges between 20.24 and 22.52% for SWP-S_S, which is higher than ERA5 (8.81–17.32%) but less than the rest of the CORDEX models (20.39–25.63% for REMO and 22.36–28.32% for HIRHAM). The rest of the SWPs have similar variance values (+ − 5%). WRF is the model that best shows the presence of the general stream undulation, especially for D_AU, in comparison with ERA5 reanalysis. This fact has been considered due to the intensity of HWs in terms of the temperature that this pattern generates, as described in the previous section. REMO and HIRHAM show the SWP-S_S with more advection at Z500 in comparison with ERA5. Due to the important amount of variance explained by this pattern (the most relevant), it is necessary to show it in a similar structure. The locations of the anticyclonic ridges (Z500) and high-pressure centers (MSLP) shown by WRF are well approximated, which is important to define the direction of wind advections to the AMB.

The daily maximum temperature of each SWP is plotted for WRF in Fig. 10 (REMO and HIRHAM are found at the bottom of S8 and S9 for the Iberian Peninsula. Due to the lack of resolution (0.1°), an analysis for the AMB scale cannot be performed. The mean temperatures for the Iberian Peninsula range between 23.52 and 24.41 °C (WRF), 22.92–24.17 °C (REMO) and 22.73–23.91 °C (HIRHAM), which are slightly underestimated in comparison to ERA5 (23.85–25.3 °C). In all three models, the mean TMAX for the SWP-S_S is the warmest pattern in the Iberian Peninsula, which matches with ERA5.

### CORDEX future scenarios

**Fig. 11:**
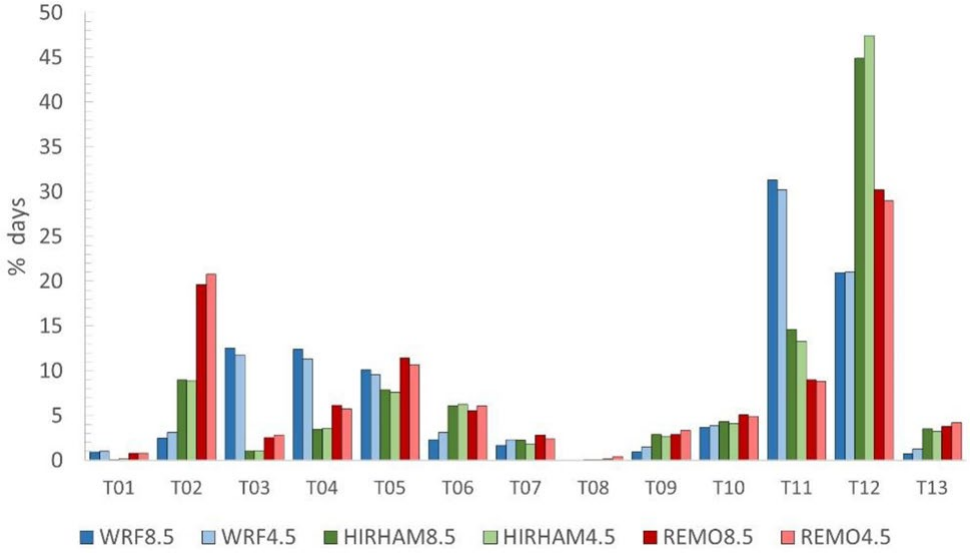
Synoptic weather types of CORDEX (scenarios 8.5 and 4.5) for the period 1951–2000. Only JJA months were selected

Having understood how well the various models perform in terms of simulating synoptic behaviors that give rise to HW episodes, we next apply the methods described above to future scenarios RCP4.5 and RCP8.5. Figure 11 shows the classification of JJA days from the model output into the thirteen synoptic weather patterns. There is no significant difference in how the days are classified between scenarios RCP4.5 and RCP8.5 for each model. The HIRHAM model, which best estimated the synoptic weather patterns in comparison with the ERA5 reanalysis, simulates an increase in JJA days in type 12 in both scenarios, going from 42.09% in the historic CORDEX to 47.4% for RCP4.5 and 44.88% for RCP8.5. However, REMO and WRF do not simulate significant increases or decreases in type 12 with respect to ERA5. CORDEX models agree that RCP8.5 increases types 05 and 11 in comparison with RCP4.5, which are patterns with northeast advections and cyclones. On the other hand, the CORDEX models simulate a decrease in the number of days of types 06, 09 and 13 in the case of RCP8.5, which are east advections and pure anticyclones.

The Mann–Kendall test was run for scenarios 4.5 (Table S10) and 8.5 (Table S11). A detailed description of all the model trends is found in supplementary material S12. Although in the CORDEX historical dataset, there are no significant trends, the CORDEX future datasets show some possible significant increasing and decreasing trends. In RCP4.5, HIRHAM is the only model that shows significant trends, which are on synoptic weather type 02 (SDT), type 12 (SIT) and type 13 (SDT). Type 12, which contains the highest number of days, shows an increasing trend from 43.37 to 50.57% of the JJA days. This increasing trend is not shown by WRF or REMO. RCP8.5 has more significant trends in all the CORDEX models. The most remarkable ones (due to the high percentage of days) are the SDT for type 02 (shown both in HIRHAM and REMO), the SIT for type 11 (shown in HIRHAM dataset) and the significant trends shown by type 12, which conflicts WRF (SDT) and REMO (SIT).

Next, the temperature percentiles of both scenarios 4.5 and 8.5 are analyzed to subsequently select the HW potential days. CORDEX models show an increase in temperatures in all their percentiles (more details in the Supplementary Materials S13), but there are differences among them. For scenario 4.5, WRF simulations result in a small increase in the 95th percentile (+ 0.69 °C) in the last climatic period (2071–2100), reaching 31.57 °C in comparison to the first period (2011–2040), which reaches 30.59 °C. For HIRHAM, the 95th percentile increases by + 3.17 °C (from 30.53 °C to 33.7 °C), and REMO increases by + 0.99 °C (from 30.58 °C to 31.57 °C).

In scenario 8.5, the CORDEX models simulate a higher increase in temperatures for all percentiles. The 95th percentile for the WRF model rises 3.18 °C, ranging from 30.9 to 34.08 °C, HIRHAM shows an increase of 5.7 °C (from 30.79 to 36.49 °C) and REMO also considers an increase of 3.89 °C (from 30.59 to 34.48 °C). All the CORDEX models and scenarios agree in an increase in HW thresholds, which are higher in scenario 8.5. For scenario 4.5, the increase in HW thresholds by periods of 10 years ranges from + 0.12 °C/10Y (WRF) to + 0.53 °C/10Y (HIRHAM), while in scenario 8.5, the 95th percentile ranges from + 0.53 °C/10Y (WRF) to + 0.95 °C/10Y (HIRHAM). WRF is the model with the lowest heating, and HIRHAM is the model that increases the most.

**Fig. 12:**
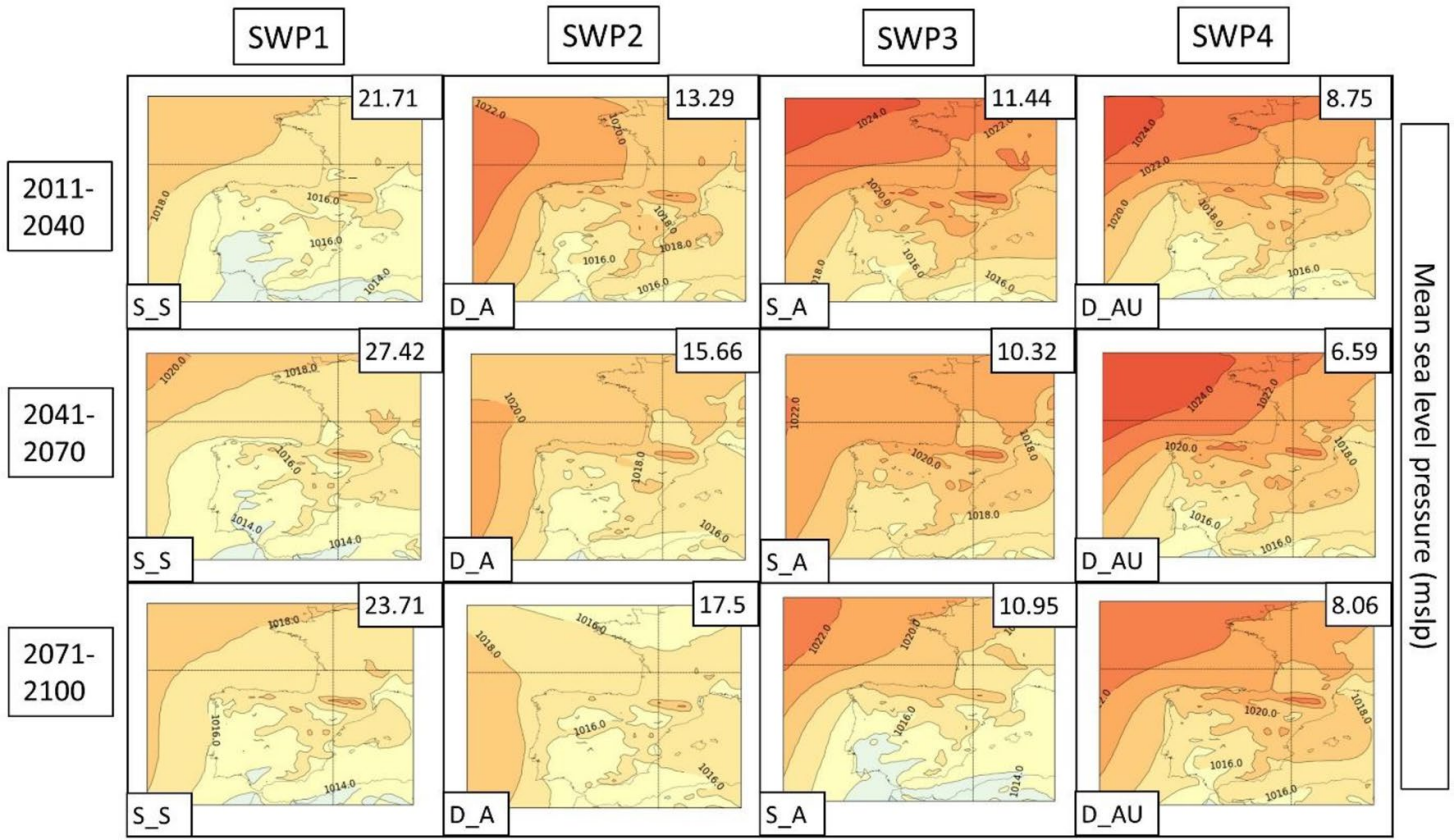
SWPs for the CORDEX RCP4.5 scenario. The MSLP variable (in hPa) is represented. Resumed figure with the periods 2011–2040, 2041–2070 and 2071–2100. Variance explained in the upper-right corner and the pattern in the bottom-left. Model: WRF

**Fig. 13:**
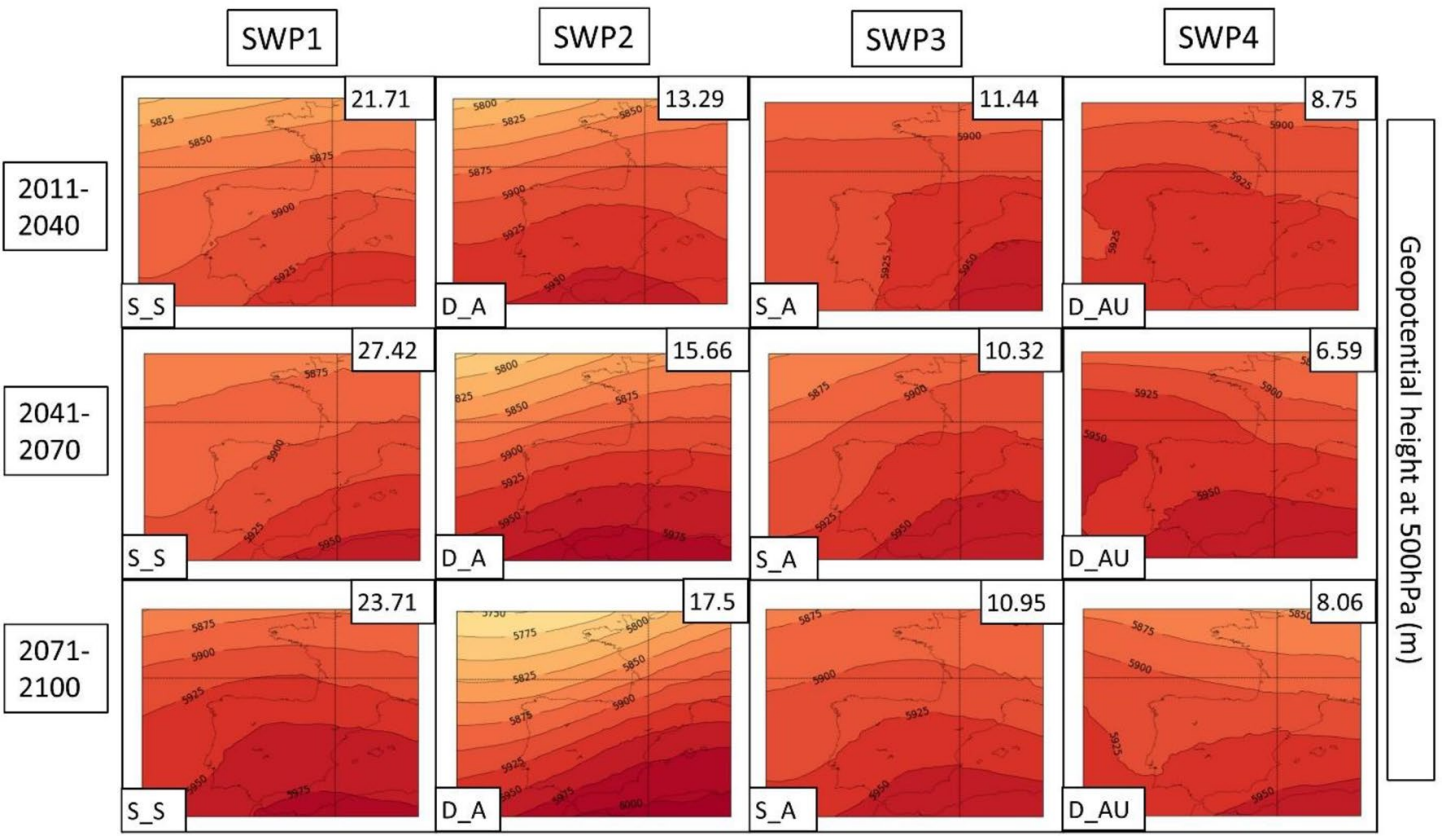
SWPs for the CORDEX RCP4.5 scenario. Geopotential height at 500 hPa (Z500) variable (in m) plotted by the climatic periods divided by files. Variance explained in the upper-right corner and the pattern in the bottom-left. Model: WRF

**Fig. 14:**
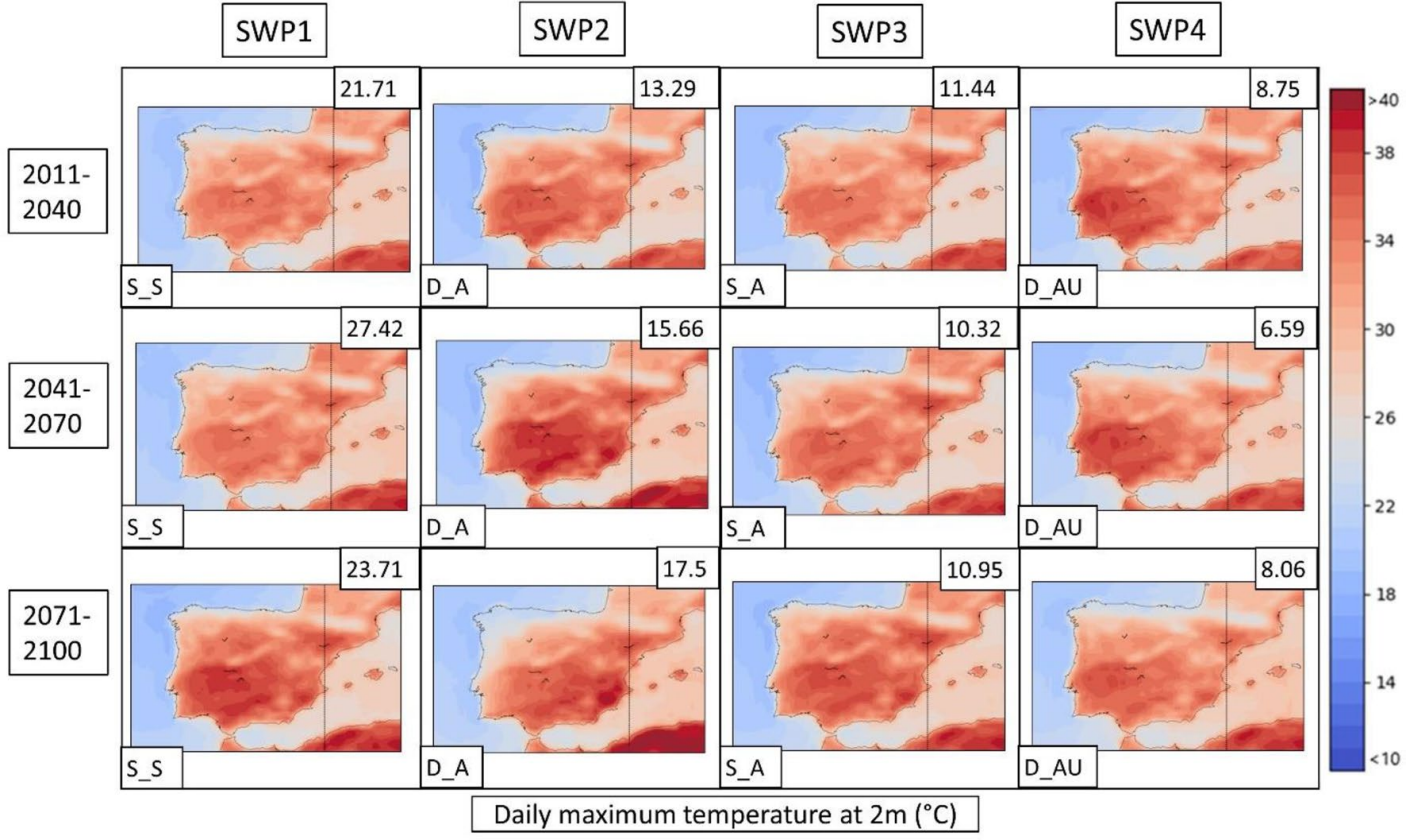
SWPs for the CORDEX RCP4.5 scenario. Daily maximum temperature at 2 m variable (in °C) plotted by the climatic periods divided by files. Variance explained in the upper-right corner and the pattern in the bottom-left. Model: WRF

**Fig. 15:**
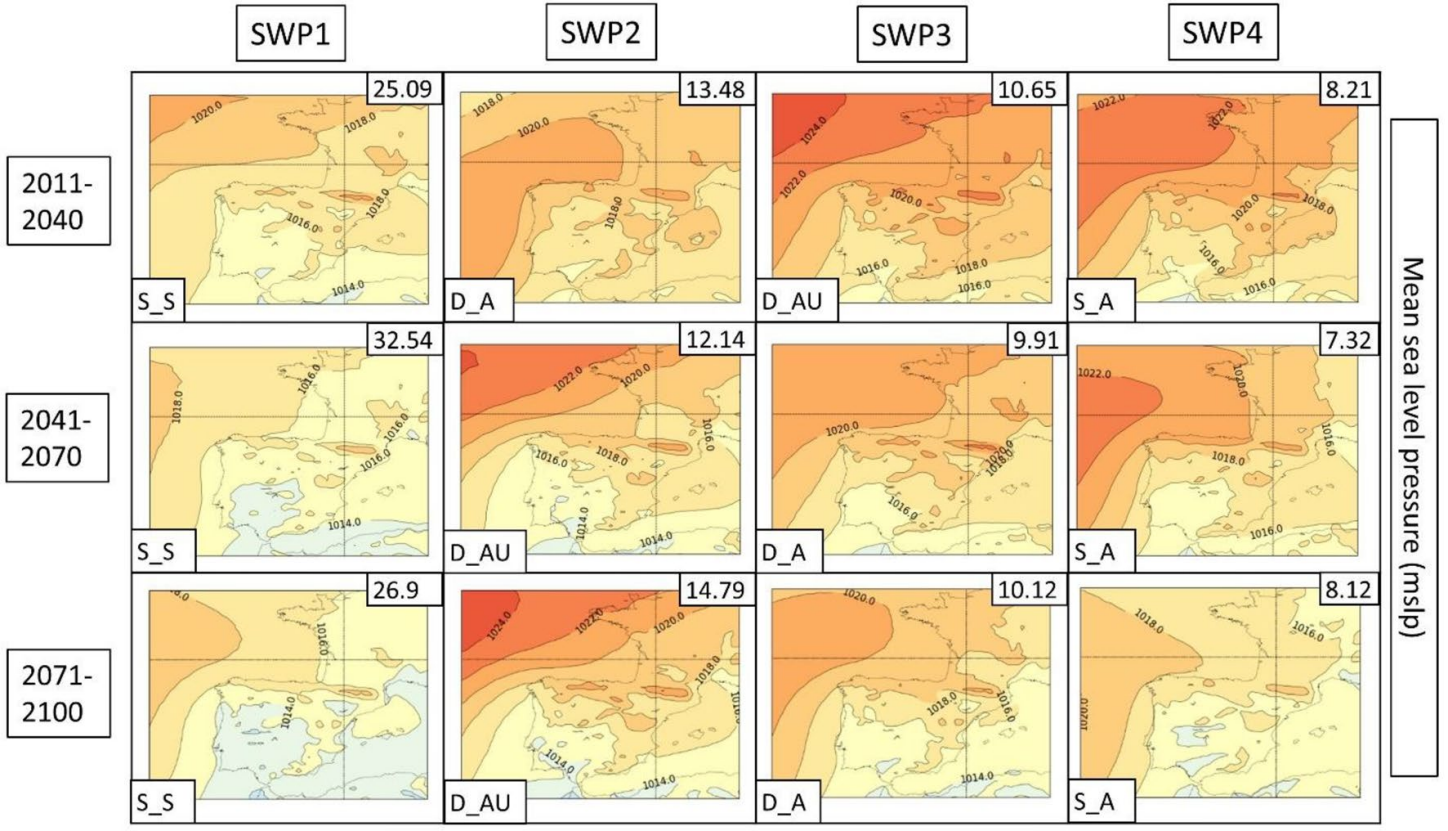
SWPs for the CORDEX RCP8.5 scenario. The MSLP variable (in hPa) is represented. Resumed figure with the periods 2011–2040, 2041–2070 and 2071–2100. Variance explained in the upper-right corner and the pattern in the bottom-left. Model: WRF

**Fig. 16:**
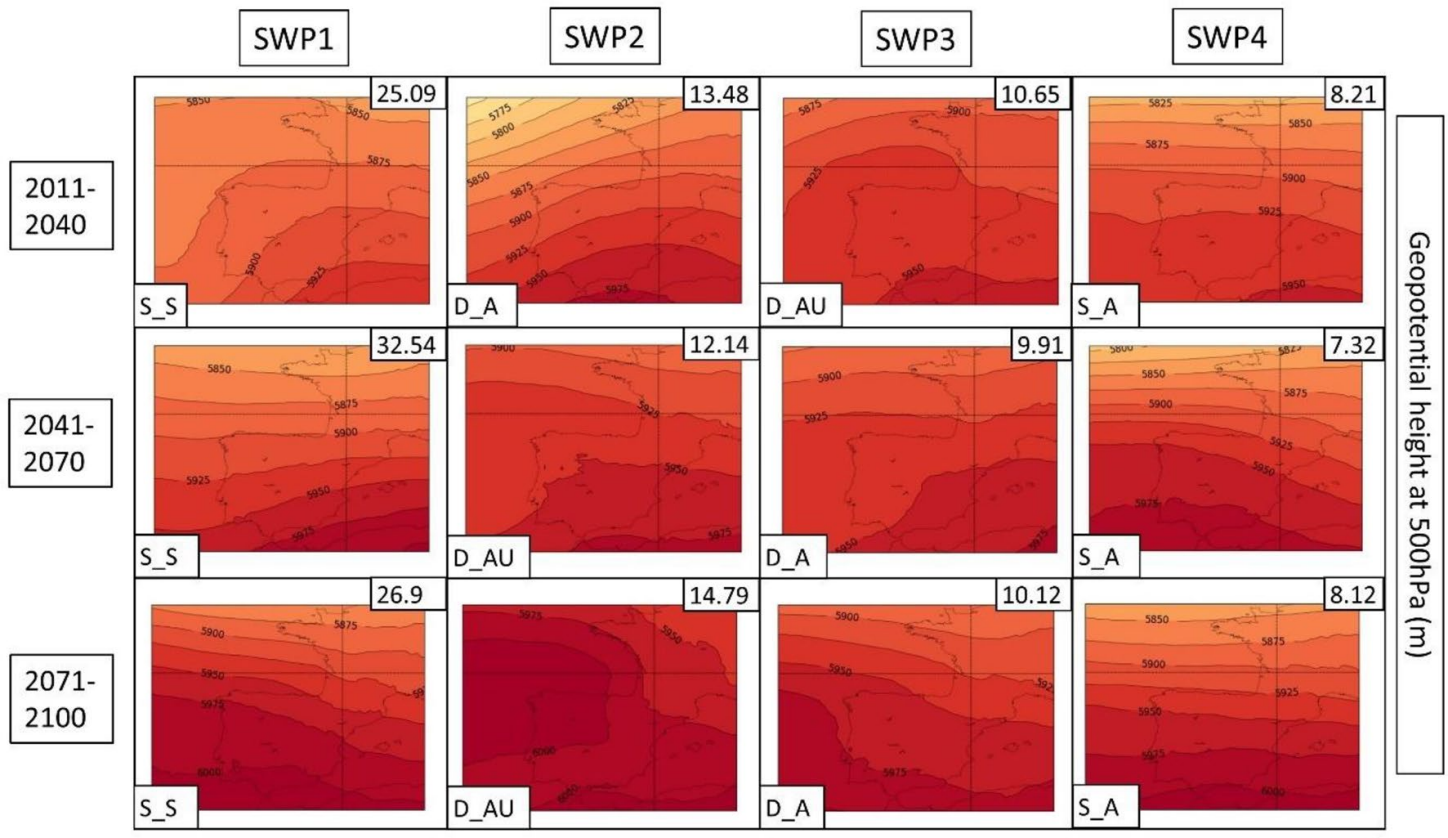
SWPs for the CORDEX RCP8.5 scenario. Geopotential height at 500 hPa (Z5000) variable (in m) plotted by the climatic periods divided by files. Variance explained in the upper-right corner and the pattern in the bottom-left. Model: WRF

**Fig. 17:**
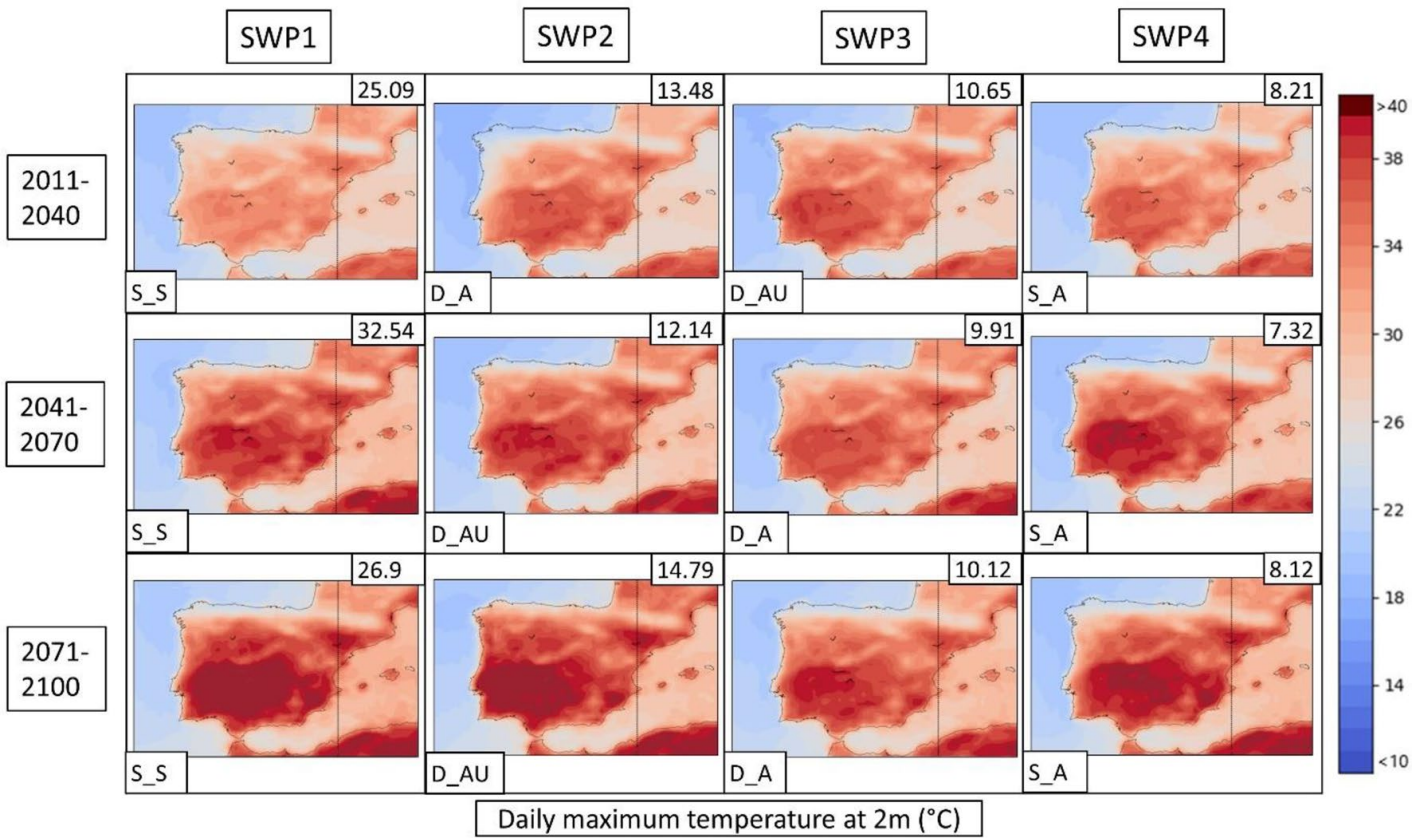
SWPs for the CORDEX RCP8.5 scenario. Daily maximum temperature at 2 m variable (in °C) plotted by the climatic periods divided by files. Variance explained in the upper-right corner and the pattern in the bottom-left. Model: WRF

Next, the PSPA method described in Sect. 3.3 is applied to the three climatic model future simulations for both scenarios. Due to the better representation of the WRF model described in the previous section, the following description only considers this model. A detailed description of all CORDEX models is found in supplementary material S14. The WRF results are represented in Figs. 12, 13, 14 for the RCP4.5 scenario and Figs. 15, 16, 17 for the RCP8.5 scenario. To reduce the number of plots in the main description, we have chosen only three climatic periods (2011–2040, 2041–2070 and 2071–2100) to represent the whole analyzed period (2011–2100) despite the seven periods (e.g., 2011–2040, 2021–2050, …, 2071–2100). The complete sequence for WRF, HIRHAM and REMO can be found in the Supplementary Material (S15–S23).

In general, terms, RCP4.5 shows four patterns (SWP_S-S, SWP-D_A, SWP-S_A and SWP-D_AU) in the same manner as the historical dataset. However, there are some differences and tendencies to mention.

**SWP-S_S: stationary and stable pattern**: The variance explained by this pattern does not have important changes, remaining the most important pattern (SWP1) with a variance that ranges between 21.71 and 27.42%, while in the historical WRF dataset, it ranges between 20.24 and 22.52%. MSLP shows the same structure as the historical period with a more intense thermal low. Z500 shows the same pattern with a more intense ridge, which is moving to higher latitudes. This fact generates higher geopotential heights in the Iberian Peninsula, with maximum values that increase from 5925 to 5975 m.

**SWP-D_A: dynamic and advective**: The variance explained by this pattern increases from 8.82–10.85% in the historical WRF dataset (SWP4) to 12.98–17.5% in WRF4.5 (SWP2). The MSLP shows a less intense blocking anticyclone that generates a more intense thermal low, covering more terrain in southern Europe. Z500 shows a similar pattern with increased advection due to the more important gradient generated by the north movement of the anticyclonic ridge.

**SWP-S_A: stationary and advective:** The explained variance by this pattern remains similar during WRF4.5 (9.9–11.84%) in comparison with WRF historical (10.87–11.81%). MSLP shows no differences in the general structure, although the blocking anticyclone is less intense, which generates a stronger thermal low. Z500 shows higher geopotential height values (+ 50 m in some cases) due to the north displacement of the ridge, although the structure remains the same.

**SWP-D_AU: dynamic, advective and undulated**: The explained variance by SWP-D_AU decreases from 13.61–14.79% (WRF historical) to 6.59–9.19% (WRF4.5), which makes this pattern SWP4 (SWP2 in the historical dataset). In this sense, WRF4.5 shows less undulation in the patterns. MSLP shows a similar structure with a slightly increased blocking anticyclone. Z500 shows less advection due to the less undulated pattern and the north shift of the anticyclonic ridge.

The daily maximum temperatures shown in Fig. 14 for WRF4.5 result in a significant increase with respect to historical WRF. WRF4.5 simulates the SWP-S_S pattern as the warmest on average in the Iberian Peninsula (26.31 °C), followed by SWP-DA_U (25.95 °C) and SWP-D_A (25.91 °C), which rise 1.93 °C, 2.03 °C and 1.75 °C, respectively, compared to the historic WRF.

The PSPA has also been applied to scenario 8.5. Figures 15 (MSLP), 16 (Z500) and 17 (TMAX) show the SWPs for WRF8.5. A detailed description of WRF, HIRHAM and REMO can be found in supplementary material S24. The complete associated charts can be found in Figures S25–S33.

The MSLP synoptic structure does not present significant changes in comparison with WRF4.5, although there are remarkable differences at Z500. According to WRF8.5, D_AU explains more variance (10.45–14.79%) than WRF4.5 (6.59–9.19%), and at the end of the period, it is the second most important pattern (SWP2). However, the structure of this pattern at Z500 has differences. The S‒SW advection at the Iberian Peninsula that WRF4.5 and WRF historical simulate is not observed at WRF8.5, which simulates an intense anticyclonic ridge located at the west of the Iberian Peninsula. Z500 also shows westerly flows in the other three SWPs, which differ from SW flows from WRF4.5. The maximum geopotential height values are increased, reaching 6000 m in extended parts of the Iberian Peninsula. The MSLP shows more intense and Mediterranean-centered thermal lows, which indicates the intense warming of land and sea.

The temperature values for scenario 8.5 are higher in the mid and late periods in comparison with WRF4.5. The pattern that shows the highest increase is SWP-DA_U, which reaches the highest values in the last period (28.12 °C in comparison to 26.26 °C in WRF4.5). The SWP-S_S shows a mean temperature value in 2071–2100 of 27.81 °C, which is higher in comparison to the 25.81 °C simulated at WRF4.5. The rest of the patterns show less important increases.

## Discussion

### Main synoptic types associated with summer days

It is important to determine the most common types of synoptic events occurring during summer days to later classify the synoptic trends of HWs. The results of the classification for the synoptic weather types applied to historical ERA5 data show that type 12 is dominant for summer days, followed by types 11 and 02. The rest of the types represent less than 10% of the summer days. Type 12 is defined as an undetermined pattern with no important advections or influence of a synoptic high- or low-pressure center (Jenkinson and Collison 1977). In some cases, these patterns are reinforced by strong irradiation, which generates mesoscalar thermal low pressures in the SW of the peninsula (Hoinka and Castro 2003). The intense solar radiation due to cloudy weather and the scarce wind on the surface and lower layers is a potential cause of HWs in the AMB (Gil et al. 2019). According to the Mann–Kendall test, this pattern shows a nonsignificant increasing trend, accounting for 37.8% of the summer days in 1951 to 40% in 2020. Types 11 and 02 are the next most frequent patterns on summer days according to ERA5, which are defined as a thermal low and anticyclonic western advection, respectively. Thermal lows are generated by intense solar radiation and stability, which rises warm air from lower atmospheric layers, generating a low pressure (Portela and Castro 1996), commonly produced after stationarity of type 12. However, western advections are dynamic situations that can advect warm and dry air masses from the interior of the peninsula (Mazon et al. 2014), generating potential HW days in the Mediterranean basin. No significant trends are found for types 11 (18.1–16.3%) or 02 (9.6–11.1%) in the 1951–2020 period.

We found a significant decreasing trend for type 07 (east advection with cutoff low above), a rare synoptic type in all climatic periods (with a frequency of 2.5% in 1951–1980 to 1.56% in 1991–2020). North advections (type 04) show a nonsignificant decreasing trend that ranges from 6.2% to 4.93%. These results show more stability in late climate periods in comparison with the middle of the XX century, which could potentially translate to more HW potential days due to the reduction in less warm advections. The mid-1970s suffered moderate cooling (− 0.03 °C/year) that was followed by significant warming (+ 0.07 °C/year) (Lionello and Scarascia 2018). This trend is reflected in the SWPs we discuss next, in which late periods show warmer and more stable patterns than the first periods.

### Main synoptic patterns associated with heatwaves at the regional and urban levels

We determined that four SWPs explain more than 50% of the HW statistical variability in the climatic periods covering 1951–2020 at the AMB: two stationary patterns (SWP-S_S and SWP-S_A) that explain 27.9% of the variance and two dynamic patterns (SWP-D_A and SWP-D_AU) that explain 22.6%. The MSLP shows, in all four cases, an anticyclone over the Atlantic that blocks the possible flux of low-pressure centers or weather fronts, generating stability with long periods of cloudless conditions. Due to the latitude of the Iberian Peninsula, cloudless conditions can intensify solar radiation flux and generate positive temperature anomalies, promoting HW conditions (Tomczyk et al. 2017). There is also a general presence of thermal lows over the Iberian Peninsula, which are deeper in the case of intense solar radiation. The Z500 variable shows more information about general circulation, such as the presence of ridges and troughs, which could indicate warm or cold intrusions. All four SWPs show an anticyclonic ridge in the Iberian Peninsula, which, depending on its position, generates different directions and intensities of advections in height.

The most stationary and static pattern associated with HWs is SWP-S_S, with a ridge that covers the entire Iberian Peninsula for at least three days. This pattern reaches a mean temperature of 34.4 °C. MSLP shows a blocking anticyclone over the Atlantic and a thermal low over the peninsula. The Z500 from SWPS-S_S presents an intense ridge centered on the peninsula, which due to its stationarity can remain for long periods of time, causing a warm air mass to be generated by the peninsula itself. These synoptic conditions increase the surface sensible heat fluxes warming the air parcels near the surface adiabatically (Zschenderlein et al. 2019), resulting in HW events.

The SWP-S_A is also stationary, although it shows a more advective pattern at Z500 with SW flux. There are two main differences between both stationary patterns (SWP-S_S and SWP-S_A). First, at MSLP, the Atlantic anticyclone is located a few km to the south in case of SWP-S_A. Second, at Z500, there is a greater geopotential height gradient due to the location of the low-pressure center in northern latitudes and the anticyclone ridge in the south. These conditions make a potential HW pattern in the E‒SE of the Iberian Peninsula, while in the NW, there are windy and colder conditions. Some studies have discussed the relation between European HWs and the tropical Atlantic conditions represented in this pattern acting as a forcing, which amplify the residence of blocking regimes and therefore increase the possibility of HWs in southern and central Europe (Cassou et al. 2005; Gil et al. 2019.).

The SWP-DA_U pattern is caused by a trough NW of the Iberian Peninsula that undulates the general circulation and generates advection from S‒SW. This pattern not only advects warm air from the African continent but also generates intrusions of Saharan dust (Sousa et al. 2019), which worsens the air quality in the Iberian Peninsula. This pattern generates the warmest HWs registered in this study due to the major south component of advection. The SWP-D_A case is characterized by the lowest surface pressures and thermal lows centered in the Mediterranean region. This is possibly generated by the temperature of the sea, which is warming 20% more than the global average (Lionello and Scarascia 2018). This pattern also shows SW advection at Z500, which supports the importance of SW advections at 500 hPa in HW episodes due to warm advection.

Zooming in at the urban level, the AMB is significantly influenced by the dynamic patterns SWP-D_A and SWP-DA_U (dynamic and advective and dynamic, advective and undulated, respectively). SWP-D_AU for 1981–2010 reaches a mean value of 35.1 °C in the AMB, followed by SWP-D_A for 1981–2010 (35 °C) and SWP-D_A for 1991–2020 (34.9 °C). Cases of S‒SW advections are potentially the warmest patterns in the AMB. The mountain ranges, especially the *Sistema Ibérico*, which is located parallel to the Mediterranean coast, generate a foehn effect throughout the E‒NE of the Iberian Peninsula in the case of W‒SW wind flow (Peña et al. 2016). In that context, prefrontal patterns such as SWP-D_AU intrinsically have an SW flow that maintains warm temperatures and dry air in the AMB but not in the W‒NW of the Iberian Peninsula.

Our analysis shows that there is an increasing trend of HWs in the AMB associated with SWP-S_S, characterized by an intense anticyclonic ridge that covers the Iberian Peninsula and reaches central Europe. This SWP generates a stationary pattern that can last for multiple days, covering a large part of western and central Europe. SWP-D_AU lost some frequency of HWs with respect to the beginning of the period (1951–1980), contrary to other studies (Zschenderlein et al. 2019). This discrepancy could be because we are explaining only 50–55% of the variance in HWs with the four SWPs and do not analyze the remaining 45–50% of the information, which we consider noise (Wold et al. 1987).

The SWP-D_A pattern shows an increase in thermal lows in the Mediterranean regions affecting the AMB due to intense solar radiation. This fact also indicates a notable warming of the sea temperature compared to the beginning of the analyzed period, which could result in more potential HW days for the AMB. This warming of the sea coincides with Lionello and Scarascia (2018), where the Mediterranean area is considered a climate change hot spot due to the expected warming of the region compared to the rest of the planet.

Our analysis of the SWPs associated with HWs in the AMB is limited due to the coarse resolution of reanalysis data, which is available at 0.25° (approx. 25 km) and is thus unable to capture human activity and building materials that exacerbate the effects of HWs (Liu et al. 2018; Morris et al. 2017). In this sense, different downscaling techniques are being investigated at the urban scale, albeit with a high degree of uncertainty (Duchêne et al. 2020; le Roy et al. 2021), but further work is needed to improve mesoscale and urban scale meteorological simulations.

### CORDEX historical simulations: analysis of limitations and advantages

Both model simulations and reanalysis data generally agree in the summer’s overall synoptic classification, representing similar SWPs in terms of the locations of high pressures, thermal lows, ridges and troughs. However, we have found some robustness and limitations on the performance of climatic models in simulating the synoptic structure of the HW episodes that are noteworthy. The CORDEX historical simulations show discrepancies among the WRF, REMO and HIRHAM models that are worth mentioning due to the possible overestimation of HW temperatures. Regarding the TMAX variable, a positive BIAS of > 1.5 °C was found in all models compared to ERA5, even after the Q-Q technique for intermodel comparison was applied. This positive BIAS agrees with what has been found in other studies such as Lhotka et al. (2018), in which the 90% quantiles were calculated separately for each simulation to remove the influence of the TMAX bias.

The MSLP and Z500 variables show similar patterns as ERA5, although the structures are shown in a less defined way (general decrease in geopotential height gradient) as a result of the low resolution of the models. The Z500 variable results in an overestimation of the intensity of the anticyclonic ridges, simulating values that range between 5900 and 5925 m, while ERA5 simulates values between 5800 and 5825 m. In this way, the three analyzed models tend to shift circulation excessively to the north. On the other hand, when analyzing the MSLP variable, the models tend to underestimate the pressure gradient, simulating more indeterminate patterns. Even so, the models are able to reflect the thermal lows and anti-cyclonic blockings in an approximate way, which contrasts with other studies where the underestimation of these blockings has been demonstrated (Scaife et al. 2010).

Several studies have analyzed the potential limitations of HW analysis with CORDEX. The elaboration of composites can lead to multiple limitations that have been discussed in several studies (Lhotka et al. 2018). These limitations are related to the fact that some effects, such as advections from opposite directions, can be canceled when applying the composite of different HW days. Since HWs can be produced by different synoptic patterns, performing a composite of different days that do not have synoptic similarity between them (despite producing HWs) can be counterproductive and limiting. This is especially important for RCMs in which land‒atmosphere iteration plays a vital role in the development of HWs. In this study, this issue is largely corrected by using the PSPA, which groups the synoptic patterns for statistical proximity, separating these patterns with different synoptic structures and avoiding this "canceling out" effect. Some articles (Sfîcă et al. 2017; Zong et al. 2022) have remarked on the importance of the connections between extremely warm periods and synoptic structures analyzed with PCA and PSPA, such as the Western Mediterranean Oscillation found in Mohammed et al. (2018).

Finally, we would like to point out the limitations in analyzing HWs at the urban scale. Due to the CORDEX resolution (0.11°), it is not possible to analyze the results at the urban scale. In that sense, some articles (Duchêne et al. 2020; le Roy et al. 2021) have elaborated downscaling techniques to consider the influence of cities on the local climate (e.g., urban heat island). However, CORDEX simulations show the main HW synoptic patterns in a way that allows an analysis of the main structures of HWs and the possible future trends, as we have shown in this study. In this sense, WRF has been considered the model that best represents the synoptic structure of HWs in terms of similar variance, pressure centers and ridge locations when compared to ERA5.

### Trends of future HW events based on CORDEX simulations

When we analyze the HW episodes of future simulations available from CORDEX based on temperature alone, we find that the 95th percentile increases every period, resulting in warmer HW episodes. For example, HIRHAM4.5 and WRF4.5 predict an increase in the 95th percentile by + 0.12 °C and + 0.53 °C every ten years, respectively, for the RCP4.5 scenario. The same trend is more pronounced for RCP8.5, with a potential increment ranging from + 0.53 °C/10Y for WRF8.5 to + 0.95 °C/10Y for HIRHAM8.5. This increase in temperature matches the findings of the TICC report (Government of Catalonia 2017), which estimates an increase of 0.8 °C this decade and 1.4 °C by 2050 compared to 1971–2000. In that sense, both the TICCC report and the results obtained in this article point to an increase in extreme temperatures that would result in more intense HWs.

Next, we determine the synoptic weather types associated with summer days of future scenarios. This step is necessary to quantify the tendency of potential HW types, such as type 12, which in ERA5 represents 40% of the summer days and 49.7% of the HW days in the 1991–2020 period. There are discrepancies between the CORDEX models for types 11 and 12. HIRHAM is the only model that shows a significant increasing trend for type 12, which would result in more potential HW days by the end of this century. However, the most notable trends are the decrease in pure anticyclones (type 13) and east advections, such as types 06 and 07, and the increase in north advections, such as types 04 and 05, indicating a more extreme climate. In that sense, all models suggest a slight tendency toward an increase in atmospheric dynamism in the summer months of JJA, generating a slightly more extreme climate than the current climate. This is especially the case for RCP8.5, where we see more advective types, such as north and northeast advections. This change in climate extremes has been found by other studies: Wang et al. (2017) used Student’s test to show that climate change features an increase in warm extremes, and Donat et al. (2016) confirmed that there is high confidence that temperature extremes have been warming since the middle of the twentieth century. In the mentioned works, they found a significant correlation between the changes in climate extremes and global warming. In our study, we find a similar relation, especially in RCP8.5. We see a major number of days classified as type 12 (+ 1.75%) and type 11 (+ 1.66%), which are considered potential HW types but, at the same time, an increase in cold advections from the north (+ 2.48%) in the 2011–2100 period.

The PSPA for the CORDEX models gives us some insight into potential trends associated with HWs in the 2011–2100 period. The Z500 variable shows an increase in geopotential height, especially in scenario 8.5. The maximum geopotential height ranges between + 50 and + 75 m in RCP4.5 and from + 75 to + 150 m in RCP8.5. This suggests that the anticyclonic ridge will shift to the north, especially in RCP8.5, advecting warm air from northern Africa. It has been proven in multiple studies (Fischer et al. 2007; Serrano-Notivoli et al. 2022) that positive 500 hPa height anomalies are the main contributor to the increase in temperatures during HWs, which agrees with what we have found in this study, where geopotential heights are increased at the end of the century. Some articles have related the increase in extreme patterns, such as anomalous anticyclonic ridges, with the waviness of both polar and subtropical jets (Maher et al. 2020; Martin 2021). According to these studies, both jets become wavier, while there are no significant trends in their average speeds. This effect generates large-scale circulation anomalies that are intrinsically linked to the HWs. Both ERA5 and CORDEX simulations show that the variable MSLP indicates an increase in thermal lows not only over the south of the peninsula, as pointed out by Jerez et al. (2012) but also over the Mediterranean region, further exacerbating HW events in the AMB.

## Conclusions

This study analyzes the past and future HWs from multiple datasets: ERA5 reanalysis (historical period 1951–2020), CORDEX historical (1951–2000) and CORDEX future (RCP4.5 and RCP8.5 for the 2011–2100 period). We have evaluated the synoptical structure and significant trends associated with HW episodes to better understand the potential HW patterns and the possible evolution not only in the recent past but also in the future. The key findings can be summarized as follows:

- The historical analysis (1950–2020) using ERA5 data concluded that the most dominant synoptic weather type for summer days in the Metropolitan Area of Barcelona is type 12 (undetermined pattern without influence of high or low pressures), followed by types 11 (thermal low or cyclone) and 02 (anticyclonic western advection).
- The PSPA analysis based on historical ERA5 data from 1950 to 2020 identified some specific patterns associated with the development of HW events. Four patterns were found for each climatic period, representing at least 50% of the total HW variance. The four patterns are divided into two groups: stationary patterns (S_S and S_A) and dynamic patterns (D_A and D_AU).
-The HW patterns with the highest temperatures on the AMB are the dynamic cases, with maximum mean temperatures of 35 °C. The dynamic patterns show advections from SW‒W that increase the temperature especially in the Mediterranean region, possibly due to the inhibition of sea breezes, which is a thermal regulator in case of high temperatures. This pattern may be influenced by the presence of a low-pressure in the NW of the Iberian Peninsula, which generates an increase of S-SW advection in the AMB. However, we have found that the stationary patterns with deep anticyclonic ridges increase the temperatures in inland areas of the Iberian Peninsula. These stationary patterns can remain for multiple days over the Iberian Peninsula, generating inland warming due to intense solar radiation. This pattern may be influenced by the static presence of the Azores anticyclone, which is commonly located over the Atlantic and influences the meteorology of the Iberian Peninsula.
- The historical CORDEX simulations, once corrected with the Q-Q technique, provide SWPs similar to those of ERA5, showing good model performance in general. This performance allows us to analyze possible future trends according to the CORDEX models for both the RCP4.5 and 8.5 scenarios. However, we found that overestimation of the geopotential height could result in overestimation of the temperatures.
- The evolution of the HWs, according to the PSPA applied to CORDEX RCP4.5 and RCP8.5, has a tendency toward the intensification of the anticyclonic ridges, which reach geopotential values ​​higher than 6050 m in the RCP8.5. This increase in the geopotential height could generate an increase in temperatures in the lower layers of the atmosphere. Furthermore, we have found that the values of geopotential height reach higher values in the case of the RCP8.5 scenario, compared with the RCP4.5, which may reflect the influence of the anthropogenic warming.
- The PSPA analysis for CORDEX future simulations shows that the S‒SW advections caused by a trough in the NW of the Iberian Peninsula will be less frequent in the case of HWs at the end of the century in comparison with the present. This fact contradicts the synoptic weather type analysis, in which RCP8.5 has an increase in atmospheric dynamism. Specifically, RCP8.5 shows a decrease in anticyclones and an increase in north advections.

This study has evidenced the limitations of applying RCMs at the urban scale for a more robust analysis of synoptic patterns of HWs and hopes to motivate future work to improve methods to determine the development and effect of future HWs in urban areas. Future research using high-resolution models with urban parameterizations is being planned, with the objective of further analyze the effects of changing different urban land uses and parameters in the future climate, focusing in HWs. In this work, we found that SW advections increase temperatures in coastal areas such as the city of Barcelona, possibly due to the inhibition of sea breezes. However, we would like to study it in more detail in future work.

### Supplementary Information

Below is the link to the electronic supplementary material.

Supplementary file1 (DOCX 12880 KB)

## Data Availability

The datasets generated during and/or analyzed during the current study are available in the URBAG-ICTA PSPA_HW repository, [https://github.com/URBAG-ICTA/PSPA_HW.git].
