# Supplementary material for: Analysis of synoptic weather patterns of heatwave events
Source: Clim Dyn. 2023 May 19;61(9-10):4679–702. doi: 10.1007/s00382-023-06828-1 (PMC10579140; doi:10.1007/s00382-023-06828-1)

Analysis of synoptic weather patterns of heatwave events

Sergi Ventura ^1^, Josep Ramon Miró ^2^, Juan Carlos Peña^2^ and Gara Villalba ^1,3^*

^1^ Sostenipra Research Group (SGR 01412), Institute of Environmental Science and Technology (MDM-2015-0552), Z Building, Universitat Autònoma de Barcelona (UAB), Campus UAB, 08193 Bellaterra, Barcelona, Spain

^2^ Department of Territory and Sustainability, Meteorological Service of Catalonia, Generalitat de Catalunya. Barcelona, Spain

^3^ Department of Chemical, Biological and Environmental Engineering, Universitat Autònoma de Barcelona (UAB), Campus UAB, 08193 Bellaterra, Barcelona, Spain

***** Corresponding author (gara.villalba@uab.cat)

**Supplementary material**

**S1: The process of Principal Sequence Pattern Analysis has required a data matrix, where x[i]j represents SLP and Z500 anomalies, varying on the n grid points ([i]) and for each of the m sequences (j). The matrix structure is in T-mode.**

| **Atm. Level** | **Days** | **Grid point** | **Sequence 1** | **Sequence 2** | **…** | **Sequence m** |
| --- | --- | --- | --- | --- | --- | --- |
| **SLP** | **D-3** | **Grid [1]** | **x_[1]1_^D-3^**(SLP) | **x_[1]2_^D-3^**(SLP) | **…** | **x_[1]m_^D-3^**(SLP) |
|  |  | **…** | **…** | **…** | **…** | **…** |
|  |  | **Grid [n]** | **x_[n]1_^D-3^**(SLP) | **x_[n]2_^D-3^**(SLP) | **…** | **x_[n]m_^D-3^**(SLP) |
|  | **…** | **…** | **…** | **…** | **…** | **…** |
|  | **D** | **Grid [1]** | **x_[1]1_^D-0^**(SLP) | **x_[1]2_^D-0^**(SLP) | **…** | **x_[1]m_^D-0^**(SLP) |
|  |  | **…** | **…** | **…** | **…** | **…** |
|  |  | **Grid [n]** | **x_[n]1_^D-0^**(SLP) | **x_[n]2_^D-0^**(SLP) | **…** | **x_[n]m_^D-0^**(SLP) |
| **Z500** | **D-3** | **Grid [1]** | **x_[1]1_^D-6^**(Z500) | **x_[1]2_^D-6^**(Z500) | **…** | **x_[1]m_^D-6^**(Z500) |
|  |  | **…** | **…** | **…** | **…** | **…** |
|  |  | **Grid [n]** | **x_[n]1_^D-3^**(Z500) | **x_[n]2_^D-3^**(Z500) | **…** | **x_[n]m_^D-3^**(Z500) |
|  | **…** | **…** | **…** | **…** | **…** | **…** |
|  | **D** | **Grid [1]** | **x_[1]1_^D-0^**(Z500) | **x_[1]2_^D-0^**(Z500) | **…** | **x_[1]m_^D-0^**(Z500) |
|  |  | **…** | **…** | **…** | **…** | **…** |
|  |  | **Grid [n]** | **x_[n]1_^D-0^**(Z500) | **x_[n]2_^D-0^**(Z500) | **…** | **x_[n]m_^D-0^**(Z500) |

**S2: Mean daily maximum temperature for every SWP in the AMB according to Spain02 dataset.**

|  | swp1 | swp2 | swp3 | swp4 |
| --- | --- | --- | --- | --- |
| 1951-1980 | 32.64 | 32.11 | 31.92 | 32.57 |
| 1961-1990 | 33.7 | 33.5 | 33.23 | 33.1 |
| 1971-2000 | 33.69 | 33.98 | 33.68 | 32.73 |
| 1981-2010 | 34.72 | 34.97 | 34.1 | 35.09 |
| 1991-2020 | 34.65 | 34.88 | 34.22 | 34.47 |

**S3: Mean daily maximum temperature for every** **SWP in Spain according to Spain02 dataset.**

|  | swp1 | swp2 | swp3 | swp4 |
| --- | --- | --- | --- | --- |
| 1951-1980 | 31.7 | 33.32 | 30.55 | 30.66 |
| 1961-1990 | 30.94 | 32.54 | 33.74 | 32.9 |
| 1971-2000 | 33.74 | 31.78 | 32.97 | 32.28 |
| 1981-2010 | 34.31 | 32.85 | 32.96 | 32.18 |
| 1991-2020 | 34.41 | 32.7 | 33.65 | 32.73 |

**S4: Mean maximum temperature maps (in °C) for every SWP in Spain according to Spain02 dataset. Sorted by climatic periods (a) 1951-1980, (b) 1961-1990 and (c) 1971-2000.**


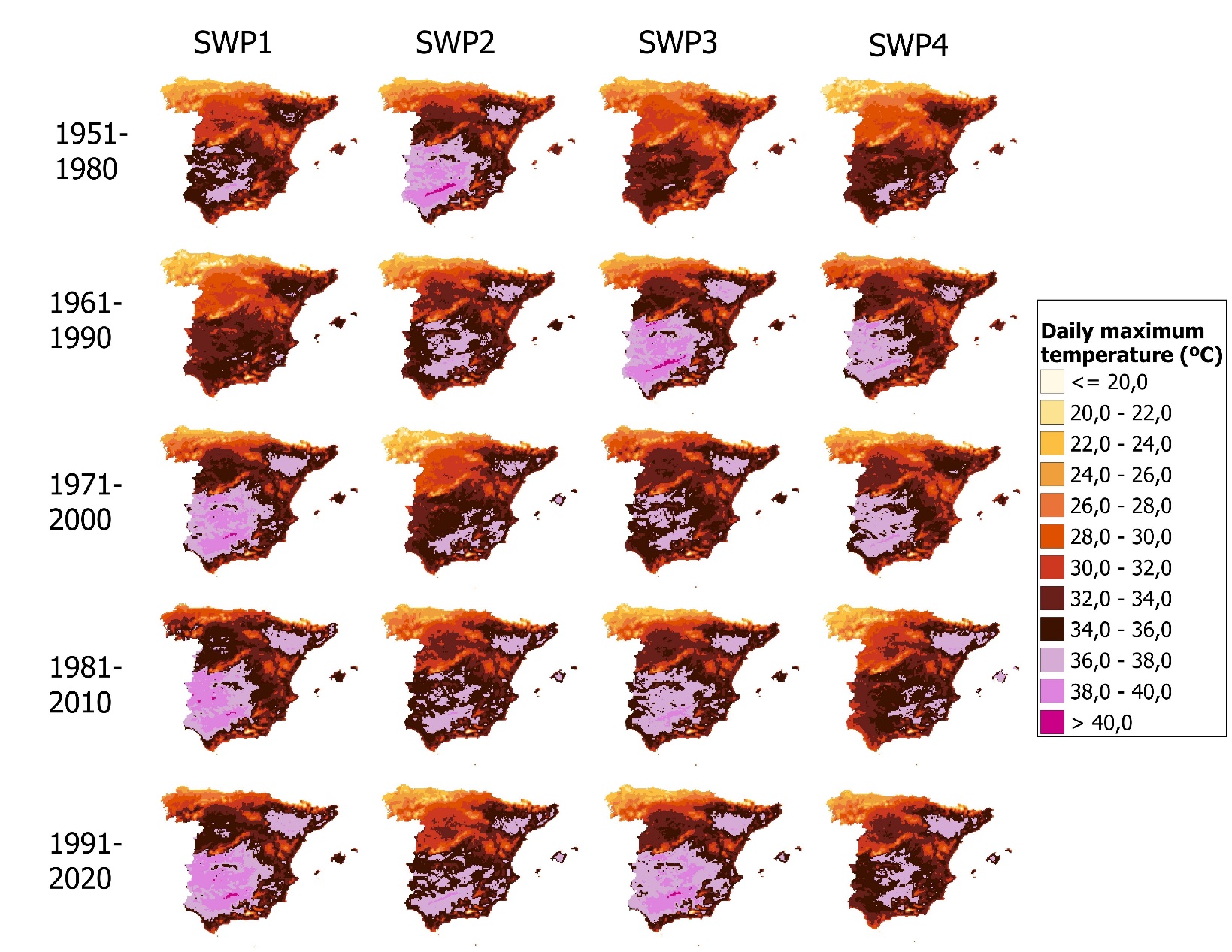


**S5: Percentage of times that SWTs occur for every model and climatic period for the JJA and CORDEX historical. Statistics Mann-Kendall (Tau-Kendall, p-value and trend. NSDT: Non-significant decreasing trend / NSIT: Non-significant increasing / SDT: Significant decreasing trend / SIT: Significant increasing trend).**

|  |  | **1951-1980** | **1961-1990** | **1971-2000** | **Tau Kendall** | **p-value** | **Trend** |
| --- | --- | --- | --- | --- | --- | --- | --- |
| **Type01** | WRF | 1.23 | 1.56 | 1.49 | 0.00 | 0.97 | X |
|  | HIRHAM | 0.33 | 0.25 | 0.33 | -0.03 | 0.81 | NSDT |
|  | REMO | 1.16 | 1.34 | 1.01 | -0.03 | 0.82 | NSDT |
| **Type02** | WRF | 4.28 | 3.99 | 3.88 | -0.08 | 0.44 | NSDT |
|  | HIRHAM | 13.26 | 12.32 | 11.99 | -0.14 | 0.18 | NSDT |
|  | REMO | 22.64 | 24.75 | 25.11 | 0.06 | 0.56 | NSIT |
| **Type03** | WRF | 7.07 | 8.15 | 7.68 | -0.04 | 0.70 | NSDT |
|  | HIRHAM | 1.45 | 1.45 | 1.81 | 0.02 | 0.86 | NSIT |
|  | REMO | 4.57 | 4.24 | 3.73 | -0.16 | 0.13 | NSDT |
| **Type04** | WRF | 8.73 | 8.77 | 8.15 | -0.01 | 0.89 | NSDT |
|  | HIRHAM | 3.19 | 2.79 | 3.19 | 0.12 | 0.27 | NSIT |
|  | REMO | 6.16 | 6.56 | 6.49 | 0.06 | 0.54 | NSIT |
| **Type05** | WRF | 10.36 | 11.05 | 10.40 | 0.01 | 0.92 | NSIT |
|  | HIRHAM | 8.01 | 8.41 | 8.55 | 0.08 | 0.46 | NSIT |
|  | REMO | 7.83 | 7.61 | 9.13 | 0.11 | 0.29 | NSIT |
| **Type06** | WRF | 5.18 | 4.60 | 4.53 | -0.05 | 0.60 | NSDT |
|  | HIRHAM | 6.70 | 7.10 | 6.41 | -0.02 | 0.89 | NSDT |
|  | REMO | 4.24 | 3.88 | 4.13 | -0.02 | 0.83 | NSDT |
| **Type07** | WRF | 3.33 | 3.33 | 3.59 | 0.11 | 0.31 | NSIT |
|  | HIRHAM | 2.61 | 3.08 | 2.61 | 0.01 | 0.90 | NSIT |
|  | REMO | 2.61 | 2.28 | 1.74 | -0.16 | 0.14 | NSDT |
| **Type08** | WRF | 0.07 | 0.00 | 0.00 | X | X | X |
|  | HIRHAM | 0.25 | 0.11 | 0.14 | X | X | X |
|  | REMO | 0.40 | 0.18 | 0.18 | X | X | X |
| **Type09** | WRF | 2.03 | 2.14 | 2.21 | -0.03 | 0.80 | NSDT |
|  | HIRHAM | 3.15 | 2.68 | 2.86 | -0.07 | 0.51 | NSDT |
|  | REMO | 3.55 | 3.48 | 3.73 | 0.02 | 0.89 | NSIT |
| **Type10** | WRF | 4.28 | 4.38 | 4.93 | 0.13 | 0.20 | NSIT |
|  | HIRHAM | 4.64 | 3.99 | 4.06 | -0.14 | 0.17 | NSDT |
|  | REMO | 5.25 | 5.33 | 5.22 | 0.09 | 0.39 | NSIT |
| **Type11** | WRF | 27.61 | 26.99 | 29.24 | 0.06 | 0.52 | NSIT |
|  | HIRHAM | 11.96 | 11.20 | 12.28 | -0.01 | 0.94 | NSDT |
|  | REMO | 9.24 | 9.28 | 8.55 | -0.10 | 0.31 | NSDT |
| **Type12** | WRF | 23.37 | 22.86 | 21.67 | -0.09 | 0.39 | NSDT |
|  | HIRHAM | 41.27 | 42.90 | 41.92 | 0.06 | 0.56 | NSIT |
|  | REMO | 27.93 | 26.81 | 26.92 | -0.10 | 0.30 | NSDT |
| **Type13** | WRF | 2.46 | 2.17 | 2.25 | -0.08 | 0.44 | NSDT |
|  | HIRHAM | 3.19 | 3.73 | 3.84 | 0.13 | 0.23 | NSIT |
|  | REMO | 4.42 | 4.28 | 4.06 | -0.03 | 0.74 | NSDT |

**S6: Box plot from observations (Fabra), ERA5, and historical quantile‒quantile adjusted datasets for three periods: 1951–1980, 1961–1990 and 1971–2000. The boxes are defined by 25–75th quantiles, and whiskers are defined by the 5–95th quantiles. The 95th percentile is quantified (°C).**


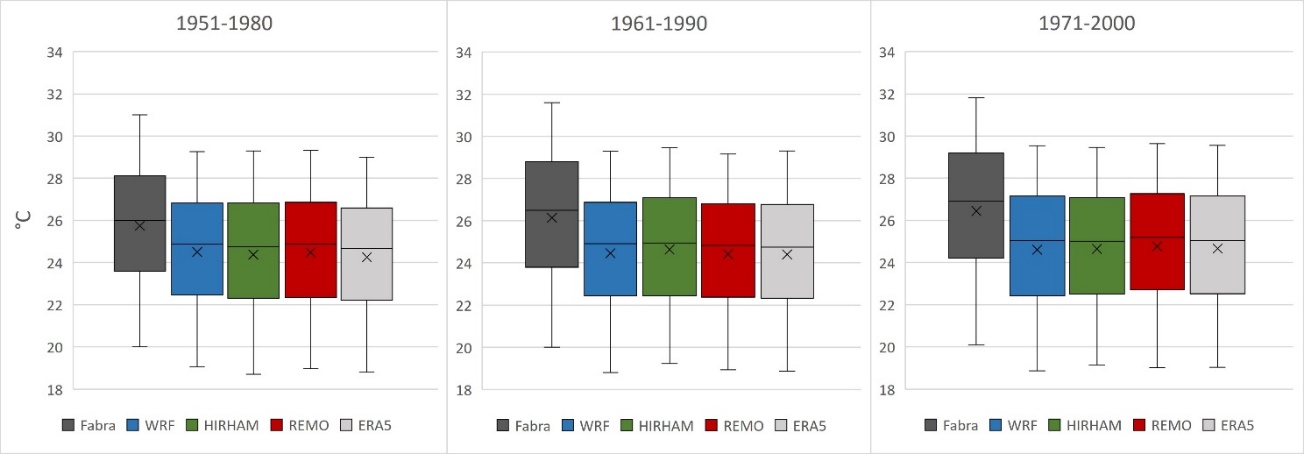


**S7: CORDEX historical simulations. Definition of the four synoptic weather patterns:**

**SWP-S_S: stationary and stable pattern.** Although all CORDEX simulations have a lack of resolution and generate the synoptic structures in a diffuse way, this pattern is also found in CORDEX datasets. In case of the MLSP variable, WRF and REMO correctly simulate a thermal low in the south and a blocking anticyclone in the NW. PSPA for HIRHAM increases the intensity of the blocking anticyclone. In Z500, all CORDEX simulations project a similar ridge in the south, even REMO and HIRHAM simulates it more advective than WRF. Moreover, WRF and HIRHAM simulate a displacement of jet stream to the north. All three models overestimate the variance for this pattern, ranging between 20.24 and 28.32%.

**SWP-S_A: stationary and advective.** MSLP indicates a situation of blocking anticyclone with thermal low over the south of the IP, which is the same pattern found in ERA5. CORDEX models do not observe remarkable changes in the intensity of the blocking anticyclone. Z500 shows a wavy situation with advection from SW, found in all three models. Although, there are differences between models. WRF simulate an intensification of the ridge and the waviness of the stream, while HIRHAM and REMO do not simulate any clear trend. The variance explained for this SWP by the models is approximated to ERA5, ranging from 8.89 to 11.81%.

**SWP-D_AU: dynamical, advective and undulated.** This pattern has been found in a less undulated structure in all three models. MSLP shows a blocking anticyclone and a thermal low over the IP. There is a general trend to the weakness of the blocking anticyclone, as well as ERA5. Z500 shows a general increasing trend of the intensity of the ridge. CORDEX simulations simulate the ridge in the SE and a low-pressure center in the NW, generating a wavy situation with an advection from SW. Although, ERA5 shows an advection with more south component. The variance explained for this pattern is well approximated to ERA5, ranging from 7.8 to 10.93%.

**SWP-D_A: dynamic and advective.** In the MSLP, all three models simulate a blocking anticyclone and a thermal low in the IP. However, this thermal low is not centered over the Mediterranean but in the IP and North Africa, so the heating of the Mediterranean is not remarkable according to CORDEX. In the case of Z500, CORDEX simulations show a similar case with an advection from SW, which has a trend to increase the waviness, as well as ERA5 does. The variance explained by this pattern in all three models is similar to ERA5, ranging from 11.96 to 15.84%.

**S8: SWPs for CORDEX historical period. Top: Mean Sea level pressure (MSLP) variable (in hPa). Middle: Geopotential height at 500 hPa (Z500) variable (in m). Bottom: Maximum daily temperature (TMAX) variable (in °C). Columns: Principal Components from SWP1 to SWP4. Files: Climatic periods from 1951-1980 to 1971-2000. Model: REMO.**
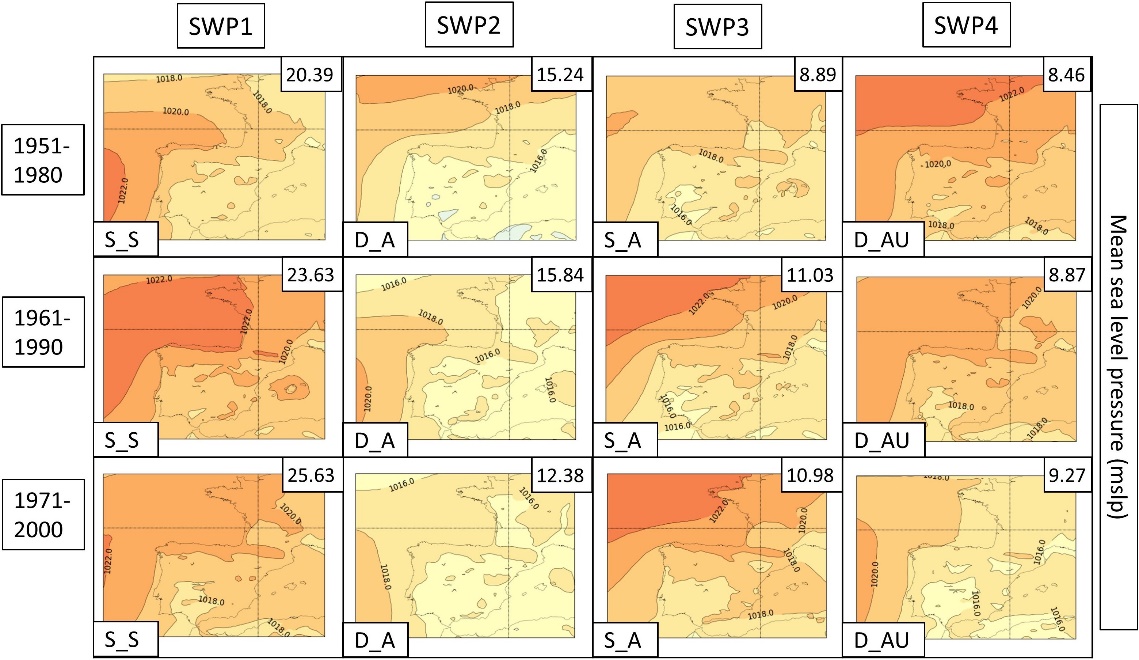

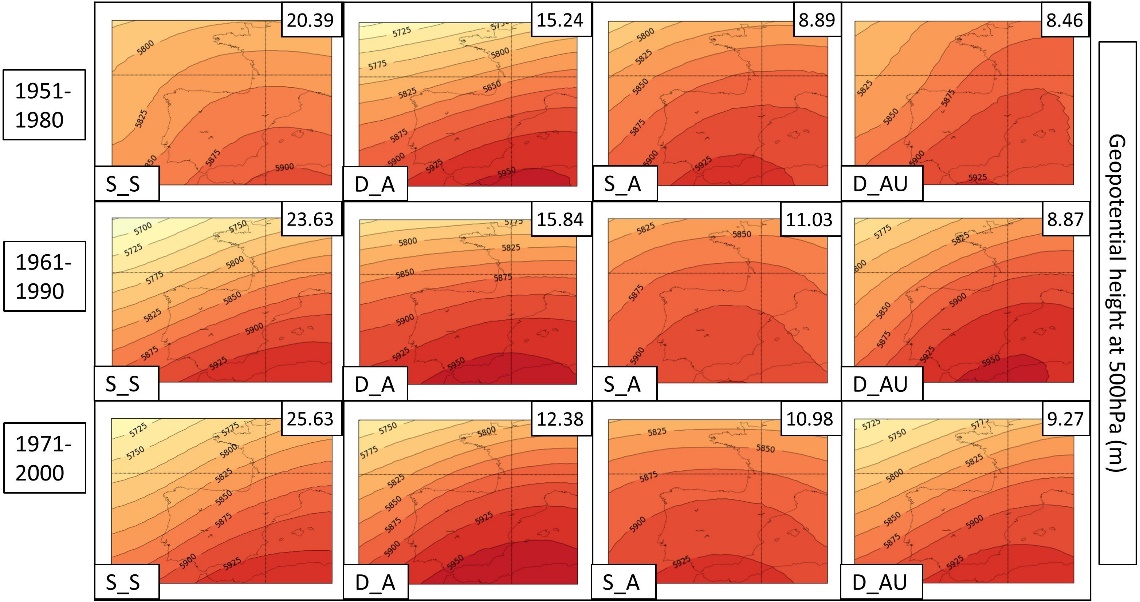

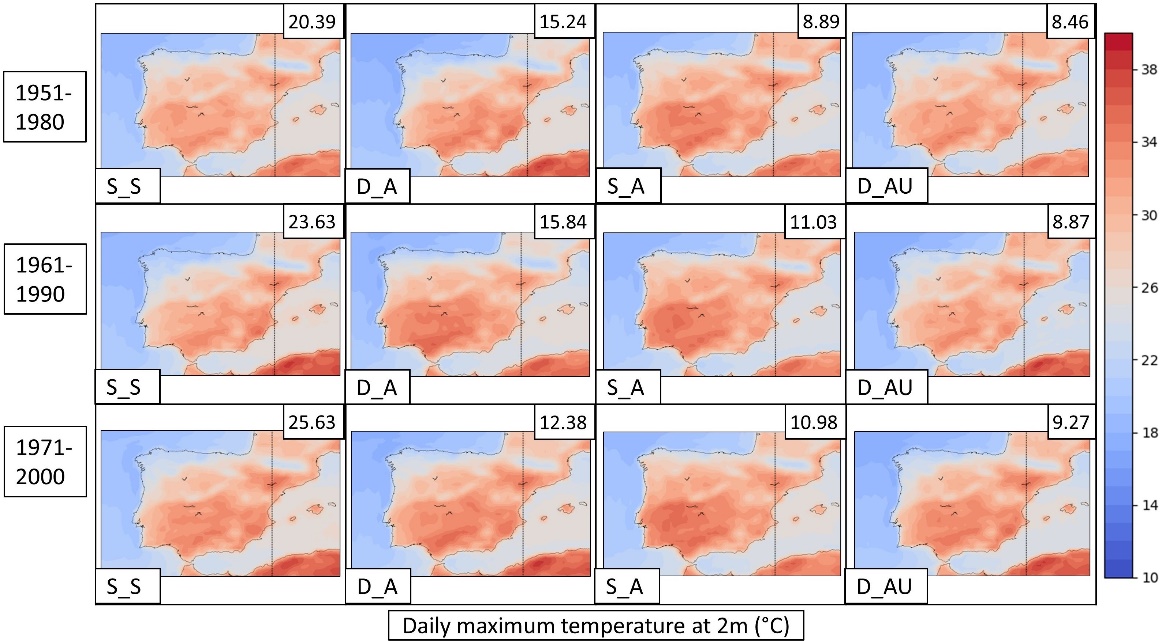


**S9: SWPs for CORDEX historical period. Top: Mean Sea level pressure (MSLP) variable (in hPa). Middle: Geopotential height at 500 hPa (Z500) variable (in m). Bottom: Maximum daily temperature (TMAX) variable (in °C). Columns: Principal Components from SWP1 to SWP4. Files: Climatic periods from 1951-1980 to 1971-2000. Model: HIRHAM.**


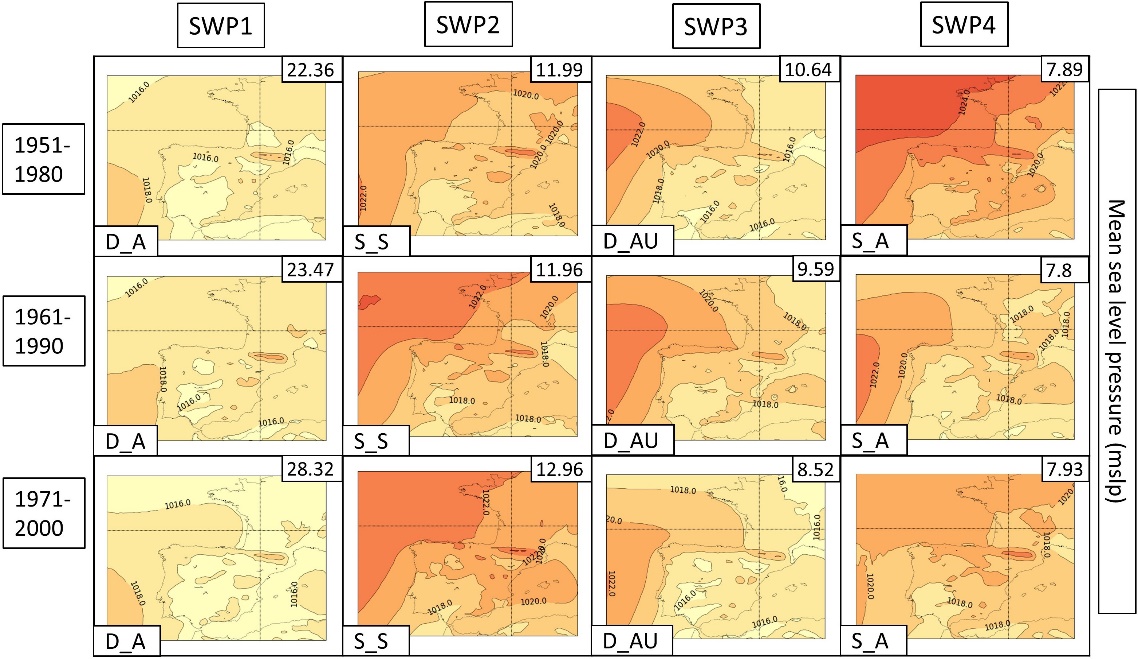

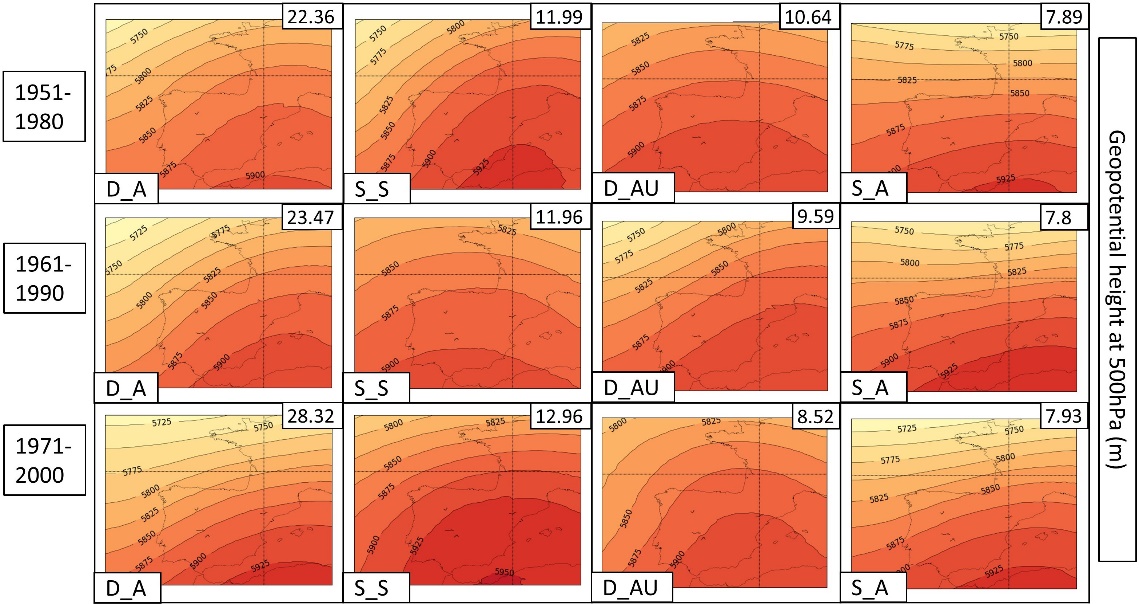

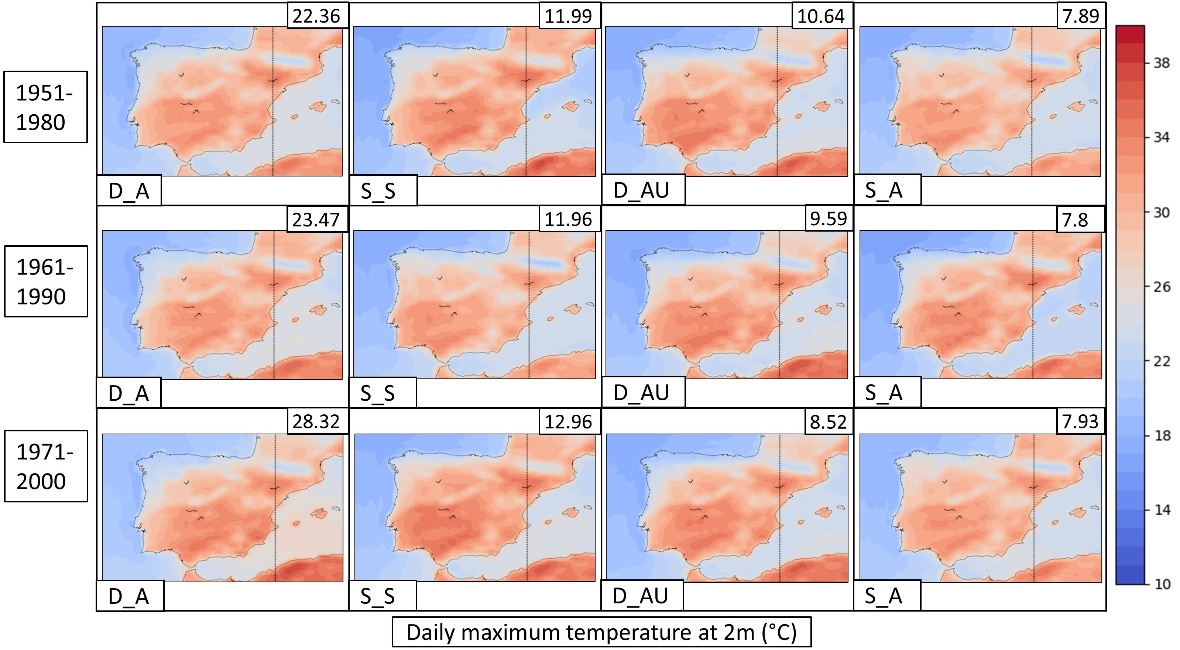


**S10: Percentage of times that SWTs occur for every model and climatic period for the JJA and RCP4.5. Statistics Mann-Kendall (Tau-Kendall, p-value and trend. NSDT: Non-significant decreasing trend / NSIT: Non-significant increasing / SDT: Significant decreasing trend / SIT: Significant increasing trend).**

|  |  | **2011-2040** | **2021-2050** | **2031-2060** | **2041-2070** | **2051-2080** | **2061-2090** | **2071-2100** | **Tau Kendall** | **p-value** | **Trend** |
| --- | --- | --- | --- | --- | --- | --- | --- | --- | --- | --- | --- |
| **Type01** | WRF | 0.94 | 1.01 | 1.12 | 0.91 | 0.94 | 0.76 | 1.09 | 0.03 | 0.75 | NSIT |
|  | HIRHAM | 0.41 | 0.22 | 0.19 | 0.15 | 0.07 | 0.07 | 0.04 | X | X | X |
|  | REMO | 0.76 | 0.69 | 0.76 | 1.05 | 1.01 | 0.76 | 0.62 | -0.05 | 0.58 | NSDT |
| **Type02** | WRF | 2.90 | 3.30 | 3.73 | 3.26 | 2.75 | 2.54 | 2.86 | 0.03 | 0.70 | NSIT |
|  | HIRHAM | 10.93 | 10.52 | 10.04 | 8.70 | 7.89 | 6.74 | 6.90 | **-0.24** | **0.00** | **SDT** |
|  | REMO | 20.25 | 19.49 | 21.85 | 21.52 | 21.96 | 20.62 | 20.62 | -0.02 | 0.83 | NSDT |
| **Type03** | WRF | 9.78 | 11.20 | 12.25 | 11.81 | 12.75 | 12.68 | 14.20 | 0.08 | 0.26 | NSIT |
|  | HIRHAM | 1.56 | 1.11 | 0.93 | 0.74 | 0.89 | 1.04 | 0.84 | -0.09 | 0.25 | NSDT |
|  | REMO | 2.83 | 2.57 | 2.90 | 3.01 | 2.97 | 2.50 | 2.68 | -0.01 | 0.87 | NSDT |
| **Type04** | WRF | 9.82 | 9.60 | 9.60 | 11.09 | 13.30 | 14.09 | 13.19 | 0.11 | 0.12 | NSIT |
|  | HIRHAM | 3.70 | 3.78 | 3.56 | 3.30 | 3.48 | 3.44 | 3.75 | 0.08 | 0.32 | NSIT |
|  | REMO | 5.43 | 5.80 | 5.65 | 5.62 | 5.87 | 5.72 | 6.05 | 0.03 | 0.72 | NSIT |
| **Type05** | WRF | 10.25 | 9.82 | 9.42 | 8.84 | 9.57 | 9.64 | 9.53 | -0.11 | 0.13 | NSDT |
|  | HIRHAM | 8.26 | 7.33 | 6.85 | 6.67 | 6.93 | 7.11 | 7.78 | -0.04 | 0.63 | NSDT |
|  | REMO | 10.54 | 9.60 | 9.02 | 10.18 | 10.65 | 11.41 | 11.23 | 0.05 | 0.54 | NSIT |
| **Type06** | WRF | 3.37 | 3.22 | 3.30 | 3.04 | 3.08 | 2.64 | 2.64 | -0.08 | 0.32 | NSDT |
|  | HIRHAM | 5.93 | 6.41 | 6.07 | 6.48 | 6.96 | 7.11 | 6.28 | 0.00 | 0.99 | X |
|  | REMO | 5.47 | 6.41 | 6.23 | 6.59 | 6.30 | 6.52 | 6.16 | 0.10 | 0.18 | NSIT |
| **Type07** | WRF | 2.28 | 2.64 | 2.57 | 2.17 | 1.81 | 1.52 | 2.25 | -0.04 | 0.58 | NSDT |
|  | HIRHAM | 2.07 | 1.70 | 1.93 | 1.74 | 2.00 | 1.89 | 1.69 | -0.09 | 0.25 | NSDT |
|  | REMO | 2.64 | 2.61 | 2.57 | 2.17 | 2.36 | 2.10 | 2.43 | -0.10 | 0.19 | NSDT |
| **Type08** | WRF | 0.00 | 0.00 | 0.00 | 0.04 | 0.04 | 0.04 | 0.00 | X | X | X |
|  | HIRHAM | 0.04 | 0.07 | 0.15 | 0.15 | 0.19 | 0.11 | 0.11 | X | X | X |
|  | REMO | 0.80 | 0.80 | 0.65 | 0.29 | 0.18 | 0.25 | 0.29 | X | X | X |
| **Type09** | WRF | 1.74 | 1.34 | 1.85 | 1.74 | 1.52 | 0.98 | 0.69 | -0.13 | 0.11 | NSDT |
|  | HIRHAM | 2.74 | 2.81 | 2.56 | 2.52 | 1.74 | 2.59 | 2.61 | -0.09 | 0.26 | NSDT |
|  | REMO | 4.02 | 3.59 | 2.90 | 2.54 | 2.79 | 3.30 | 3.44 | -0.02 | 0.79 | NSDT |
| **Type10** | WRF | 4.17 | 4.17 | 4.09 | 4.35 | 4.35 | 4.67 | 3.30 | -0.07 | 0.33 | NSDT |
|  | HIRHAM | 4.41 | 4.11 | 4.26 | 4.04 | 4.15 | 3.81 | 3.75 | -0.06 | 0.45 | NSDT |
|  | REMO | 4.64 | 5.25 | 4.67 | 4.67 | 4.75 | 5.47 | 5.51 | 0.07 | 0.32 | NSIT |
| **Type11** | WRF | 32.21 | 30.58 | 30.43 | 30.76 | 28.41 | 28.30 | 28.26 | -0.01 | 0.93 | NSDT |
|  | HIRHAM | 12.89 | 11.78 | 11.93 | 13.04 | 13.04 | 14.26 | 13.87 | 0.01 | 0.85 | NSIT |
|  | REMO | 9.46 | 8.91 | 8.70 | 8.73 | 8.33 | 8.66 | 8.15 | -0.04 | 0.56 | NSDT |
| **Type12** | WRF | 20.62 | 21.34 | 20.22 | 21.01 | 20.65 | 21.12 | 21.01 | -0.01 | 0.88 | NSDT |
|  | HIRHAM | 43.37 | 46.15 | 47.22 | 48.37 | 49.26 | 49.26 | 50.57 | **0.19** | **0.01** | **SIT** |
|  | REMO | 28.80 | 30.04 | 30.00 | 29.31 | 28.51 | 28.37 | 28.88 | 0.01 | 0.89 | NSIT |
| **Type13** | WRF | 1.92 | 1.78 | 1.41 | 0.98 | 0.83 | 1.01 | 0.98 | -0.11 | 0.19 | NSDT |
|  | HIRHAM | 3.70 | 4.00 | 4.33 | 4.11 | 3.41 | 2.56 | 1.80 | **-0.17** | **0.03** | **SDT** |
|  | REMO | 4.35 | 4.24 | 4.09 | 4.31 | 4.31 | 4.31 | 3.95 | -0.10 | 0.19 | NSDT |

**S11: Percentage of times that SWTs occur for every model and climatic period for the JJA and RCP8.5. Statistics Mann-Kendall (Tau-Kendall, p-value and trend. NSDT: Non-significant decreasing trend / NSIT: Non-significant increasing / SDT: Significant decreasing trend / SIT: Significant increasing trend).**

|  |  | **2011-2040** | **2021-2050** | **2031-2060** | **2041-2070** | **2051-2080** | **2061-2090** | **2071-2100** | **Tau Kendall** | **p-value** | **Trend** |
| --- | --- | --- | --- | --- | --- | --- | --- | --- | --- | --- | --- |
| **Type01** | WRF | 1.23 | 1.16 | 1.05 | 0.83 | 0.76 | 0.62 | 0.72 | -0.13 | 0.12 | NSDT |
|  | HIRHAM | 0.22 | 0.22 | 0.14 | 0.04 | 0.04 | 0.04 | 0.04 | X | X | X |
|  | REMO | 1.15 | 1.04 | 0.74 | 0.74 | 0.67 | 0.45 | 0.41 | **-0.18** | **0.03** | **SDT** |
| **Type02** | WRF | 2.46 | 2.93 | 3.08 | 2.64 | 2.14 | 2.39 | 2.50 | 0.01 | 0.87 | NSIT |
|  | HIRHAM | 11.92 | 10.91 | 9.89 | 9.17 | 8.22 | 6.92 | 5.70 | **-0.40** | **0.00** | **SDT** |
|  | REMO | 20.84 | 20.99 | 19.61 | 19.76 | 19.21 | 19.02 | 18.24 | **-0.17** | **0.02** | **SDT** |
| **Type03** | WRF | 9.46 | 11.63 | 13.70 | 13.59 | 13.70 | 14.24 | 15.51 | 0.17 | **0.02** | NSIT |
|  | HIRHAM | 1.09 | 0.72 | 0.62 | 0.83 | 0.91 | 1.34 | 1.05 | -0.01 | 0.89 | NSDT |
|  | REMO | 3.19 | 2.38 | 1.67 | 1.86 | 2.60 | 2.93 | 2.30 | -0.10 | 0.20 | NSDT |
| **Type04** | WRF | 11.38 | 12.61 | 12.79 | 11.16 | 11.56 | 12.43 | 15.11 | **0.20** | **0.01** | **SIT** |
|  | HIRHAM | 3.41 | 3.59 | 3.19 | 3.66 | 3.59 | 3.70 | 3.26 | 0.04 | 0.64 | NSIT |
|  | REMO | 5.91 | 7.17 | 6.95 | 6.95 | 5.68 | 5.76 | 5.61 | -0.02 | 0.81 | NSDT |
| **Type05** | WRF | 9.46 | 8.73 | 9.35 | 10.29 | 11.20 | 11.05 | 10.00 | 0.04 | 0.55 | NSIT |
|  | HIRHAM | 7.57 | 7.07 | 7.93 | 8.04 | 7.43 | 8.19 | 8.02 | 0.00 | 0.96 | X |
|  | REMO | 10.70 | 11.22 | 11.18 | 10.74 | 11.74 | 11.89 | 13.11 | **0.17** | **0.02** | **SIT** |
| **Type06** | WRF | 3.33 | 3.15 | 2.64 | 2.25 | 1.96 | 1.78 | 1.45 | **-0.20** | **0.01** | **SDT** |
|  | HIRHAM | 6.34 | 6.52 | 6.63 | 6.16 | 6.78 | 6.38 | 5.70 | -0.07 | 0.37 | NSDT |
|  | REMO | 5.01 | 4.79 | 5.13 | 5.27 | 6.09 | 5.83 | 6.17 | 0.13 | 0.08 | NSIT |
| **Type07** | WRF | 2.32 | 2.25 | 2.43 | 1.70 | 1.23 | 0.80 | 0.94 | **-0.17** | **0.04** | **SDT** |
|  | HIRHAM | 2.07 | 2.17 | 2.21 | 2.57 | 2.39 | 2.03 | 2.10 | 0.03 | 0.73 | NSIT |
|  | REMO | 3.31 | 2.67 | 2.49 | 2.79 | 2.64 | 2.90 | 2.49 | -0.12 | 0.13 | NSDT |
| **Type08** | WRF | 0.00 | 0.00 | 0.00 | 0.00 | 0.00 | 0.00 | 0.00 | X | X | X |
|  | HIRHAM | 0.07 | 0.11 | 0.22 | 0.18 | 0.18 | 0.07 | 0.07 | X | X | X |
|  | REMO | 0.26 | 0.15 | 0.04 | 0.07 | 0.11 | 0.15 | 0.15 | X | X | X |
| **Type09** | WRF | 1.70 | 1.38 | 1.09 | 0.87 | 0.62 | 0.40 | 0.29 | **-0.28** | **0.00** | **SDT** |
|  | HIRHAM | 3.01 | 3.62 | 3.12 | 2.86 | 2.21 | 2.54 | 2.77 | 0.00 | 0.97 | X |
|  | REMO | 3.45 | 2.93 | 2.67 | 2.90 | 2.90 | 2.75 | 2.38 | -0.09 | 0.22 | NSDT |
| **Type10** | WRF | 3.99 | 4.17 | 3.91 | 4.02 | 3.99 | 3.77 | 3.08 | -0.12 | 0.12 | NSDT |
|  | HIRHAM | 4.09 | 4.64 | 5.11 | 5.22 | 4.71 | 3.91 | 3.71 | -0.01 | 0.90 | NSDT |
|  | REMO | 4.64 | 4.72 | 5.39 | 5.68 | 5.46 | 5.57 | 4.68 | -0.05 | 0.47 | NSDT |
| **Type11** | WRF | 31.45 | 29.49 | 28.22 | 29.67 | 30.51 | 32.46 | 31.34 | -0.03 | 0.69 | NSDT |
|  | HIRHAM | 11.70 | 13.01 | 11.67 | 13.55 | 15.65 | 19.38 | 18.67 | **0.29** | **0.00** | **SIT** |
|  | REMO | 8.58 | 8.40 | 9.96 | 10.14 | 9.62 | 8.77 | 8.17 | -0.02 | 0.84 | NSDT |
| **Type12** | WRF | 22.10 | 21.52 | 20.98 | 22.25 | 21.74 | 19.75 | 18.73 | **-0.15** | **0.05** | **SDT** |
|  | HIRHAM | 44.09 | 43.70 | 45.14 | 44.17 | 44.06 | 43.08 | 46.44 | 0.07 | 0.36 | NSIT |
|  | REMO | 29.20 | 30.61 | 30.87 | 29.31 | 28.97 | 29.94 | 32.54 | **0.16** | **0.03** | **SIT** |
| **Type13** | WRF | 1.12 | 0.98 | 0.76 | 0.72 | 0.62 | 0.33 | 0.33 | **-0.23** | **0.01** | **SDT** |
|  | HIRHAM | 4.42 | 3.73 | 4.13 | 3.55 | 3.84 | 2.43 | 2.47 | **-0.18** | **0.02** | **SDT** |
|  | REMO | 3.75 | 2.93 | 3.31 | 3.79 | 4.31 | 4.05 | 3.75 | -0.03 | 0.65 | NSDT |

**S12: Mann Kendall description for CORDEX 4.5 and 8.5:**

The synoptic weather types are analyzed individually ignoring these patterns that occur in less than 5% of the days for not being significant. Type02 has a significant decreasing trend for HIRHAM model at both RCPs (largest decrease for RCP8.5) and for REMO RCP8.5. In this case, HIRHAM and REMO agree that anticyclonic western advections will be reduced, with greater reduction in RCP8.5. Types 04 and 05, which are northeast and north advections, have a significant increasing trend only for one model in the scenario 8.5 (WRF and REMO respectively). Thus, RCP8.5 has more probabilities to have an increase of north advections in comparison with RCP4.5. On the other hand, east advections (types 06 and 07) do not have a significant trend in RCP4.5, but a significant decreasing trend is found at the WRF simulations, following the same trend than past reanalysis. Type 11 generates some discrepancies between models. WRF and REMO show a non-significant decreasing trend while HIRHAM forecast a significant increasing trend in the scenario 8.5. Type 12 also generates discrepancies between the models. On the one hand, WRF has a significant decreasing trend at RCP8.5. However, HIRHAM (at the RCP4.5) and REMO (at the RCP8.5) show a significant increasing trend. In that sense, no clear estimations for type 12 can be done. Finally, for the type 13, HIRHAM simulates a significant decreasing trend for RCP4.5 and 8.5 while REMO do the same at the RCP8.5.

**S13: Box plot from RCP4.5 and RCP8.5 simulations for WRF, HIRHAM and REMO JJA 2011–2100 period. The boxes are defined by the 25–75^th^ percentile, and whiskers are defined by the 5–95th percentile. The 95th percentile is indicated at the top of the whisker.**


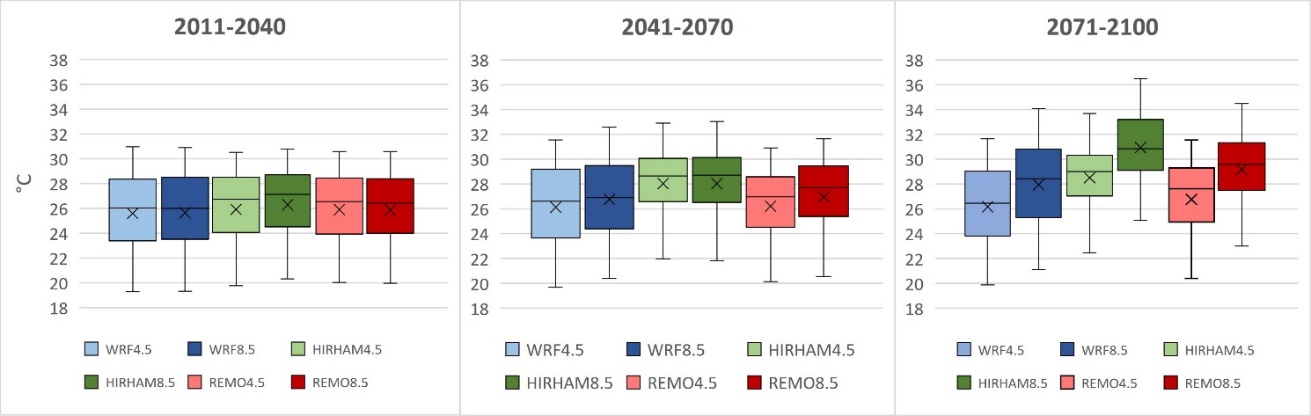


**S14: Description of the CORDEX 4.5 synoptic weather patterns:**

**SWP-S_S: stationary and stable pattern.** MSLP shows an increase of the intensity of the blocking anticyclone in the WRF model, REMO simulates an undetermined pattern and HIRHAM has a trend to reduce the blocking intensity. Z500 shows a north shift of the jet stream in all the models, which generates an increase of the intensity of the anticyclonic ridge in the IP. The values of geopotential height are increased in more than 50 meters in all three models from 1971-2000 to 2071-2100. The variance explained for the SWP-S_S is increased in all three models. At REMO, the increase ranges from 20.39-25.63% to 22.61-29.27%.

**SWP-S_A: stationary and advective.** MSLP shows a general intensification of the thermal low, which, in some cases, is especially intense over the Mediterranean due to the warm sea. There is a general intensity reduction trend of the blocking anticyclone. Z500 shows differences between the models, although the three models coincide in an increase of the geopotential height (especially HIRHAM, which increase ranges between 50-100 m). REMO and HIRHAM project more advection and WRF shows no remarkable trends. The variance explained for this pattern do not show any trend in comparison with the historical period and ranges between 5.67 and 11.85% in the models.

**SWP-D_AU: dynamical, advective and undulated.** MSLP shows a blocking anticyclone and a thermal low over the IP with no significative trends in the models. Z500 shows more differences between the models. WRF projects an undefined pattern with undulation in the west of the IP, although REMO and HIRHAM show more advection from SW with an increase of the undulation in the circulation. The variance explained by this pattern ranges between 6.59 and 10.33%.

**SWP-D_A: dynamic and advective.** In the case of WRF, the MSLP shows a reduction of the blocking anticyclone intensity, which generates an increase of the surface thermal low. REMO does not simulate any important trend and HIRHAM shows an undefined pattern with low pressures over Europe and the Mediterranean. Z500 is defined by a ridge in the south of the IP and a dynamic SW advection at the AMB. WRF projects an increase of the advection due to the presence of a low-pressure in the NW of the IP increasing the undulation of the general circulation. REMO also shows an increase of the undulation with a northward shift of the anticyclonic ridge. There is a general projection of an increase of the geopotential height in all the region, increasing the maximum peaks by 50-75m. All three models project an increase of the frequency for this pattern, which ranges from 29.42 to 33% for HIRHAM and from 11.25 to 17.5% for WRF and REMO.

**S15. SWPs for CORDEX RCP4.5 scenario. MSLP variable (in hPa) is represented. Complete figure with periods 2011-2100. Variance explained in the upper-right corner and SWP in the bottom-left. Model: WRF.**


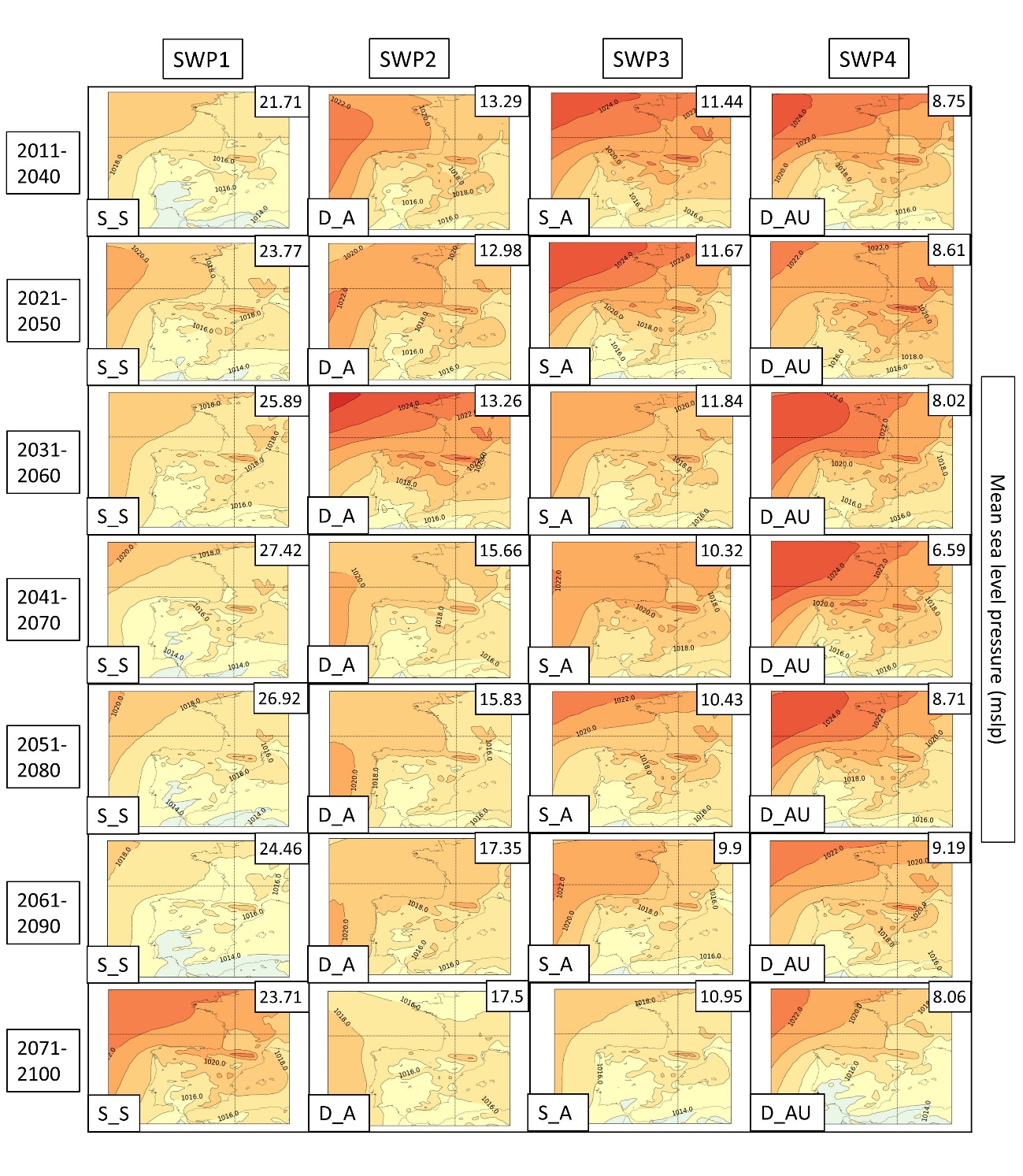


**S16. SWPs for CORDEX RCP4.5 scenario.** **Geopotential height at 500 hPa (Z500) variable (in m). Complete figure with periods 2011-2100. Variance explained in the upper-right corner and SWP in the bottom-left. Model: WRF.**


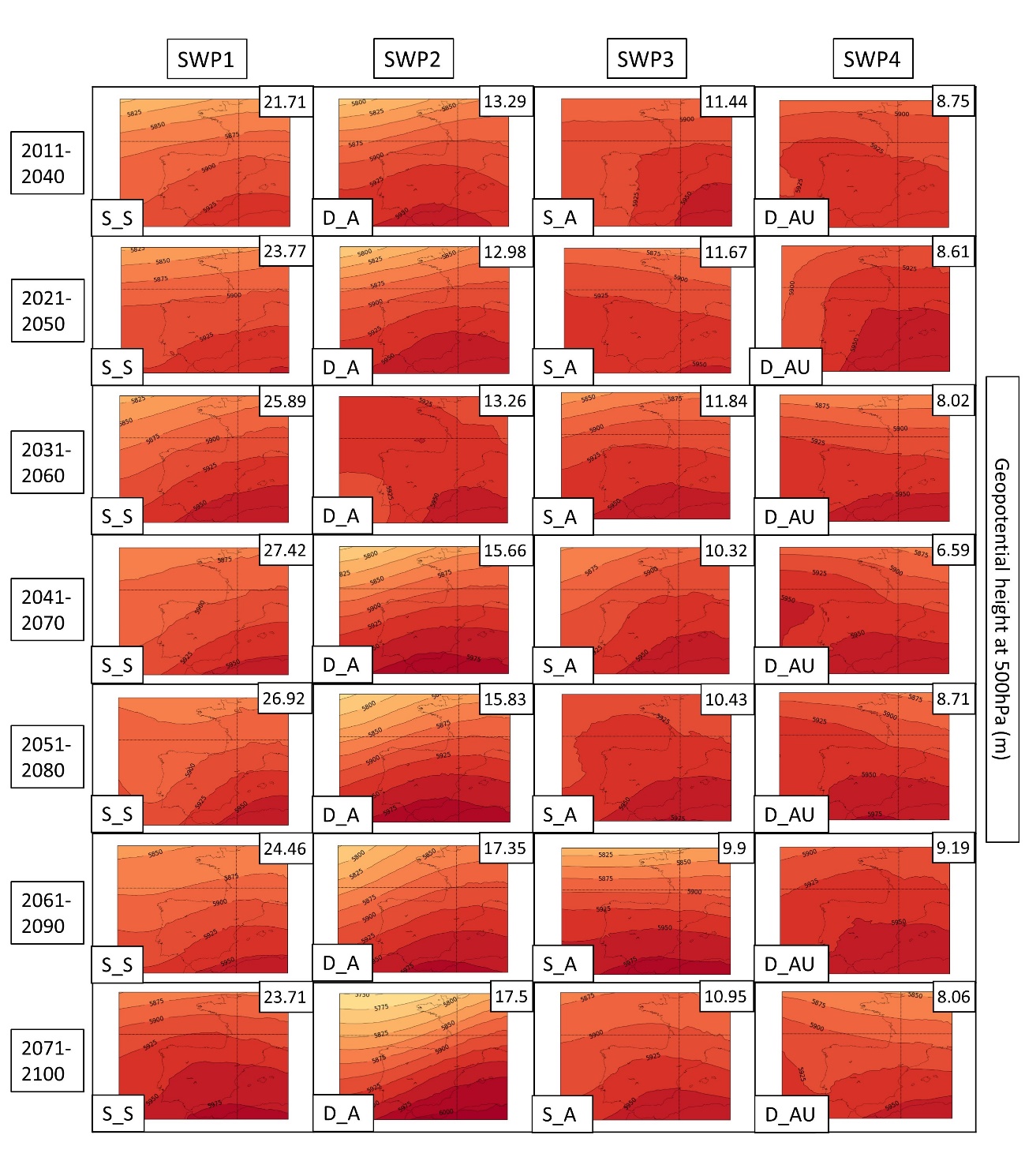


**S17. SWPs for CORDEX RCP4.5 scenario.** **Daily maximum temperature at 2m variable (in °C).** **Complete figure with periods 2011-2100. Variance explained in the upper-right corner and SWP in the bottom-left. Model: WRF.**


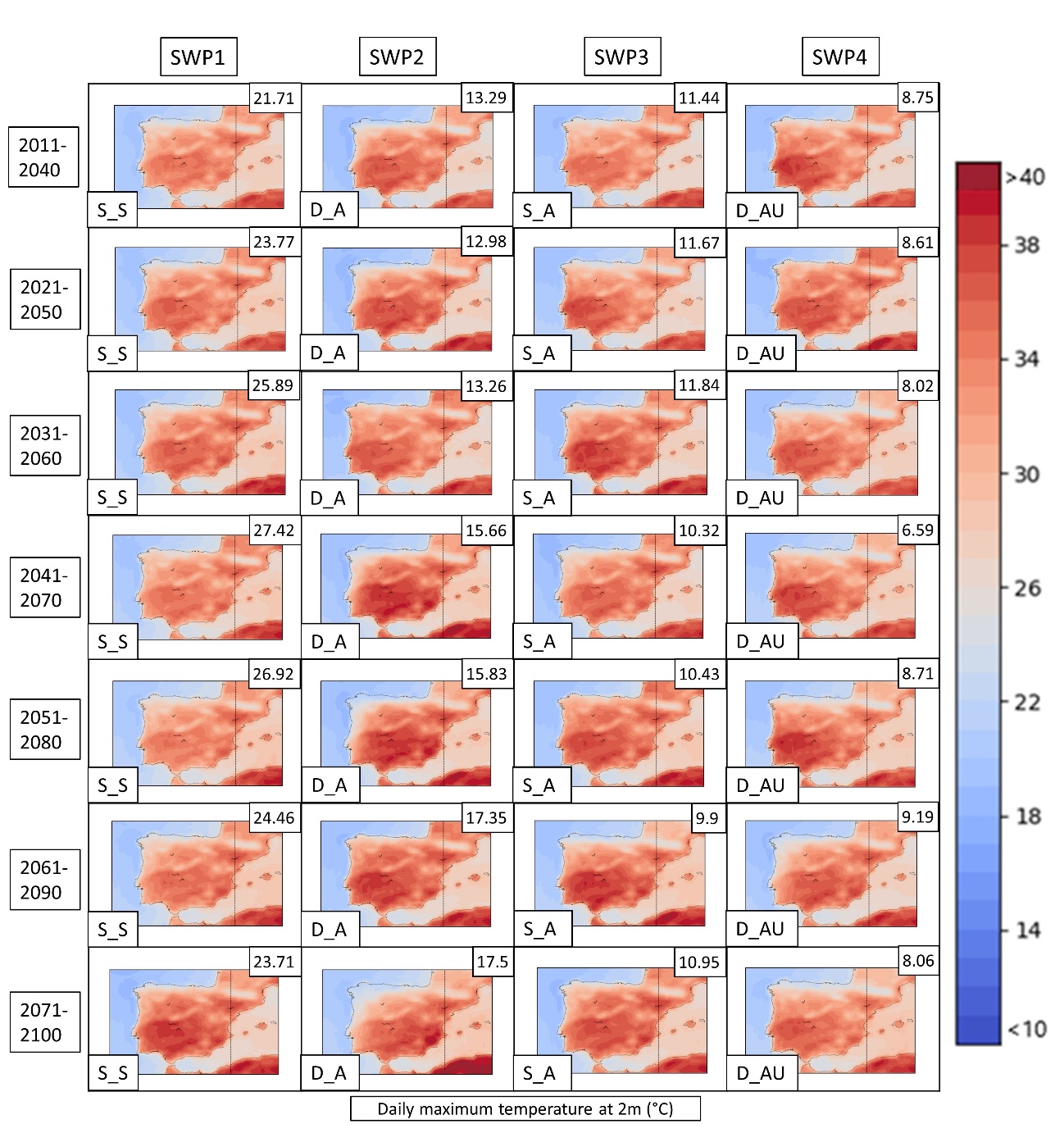


**S18. SWPs for CORDEX RCP4.5 scenario. MSLP variable (in hPa) is represented. Complete figure with periods 2011-2100. Variance explained in the upper-right corner and SWP in the bottom-left. Model: REMO.**


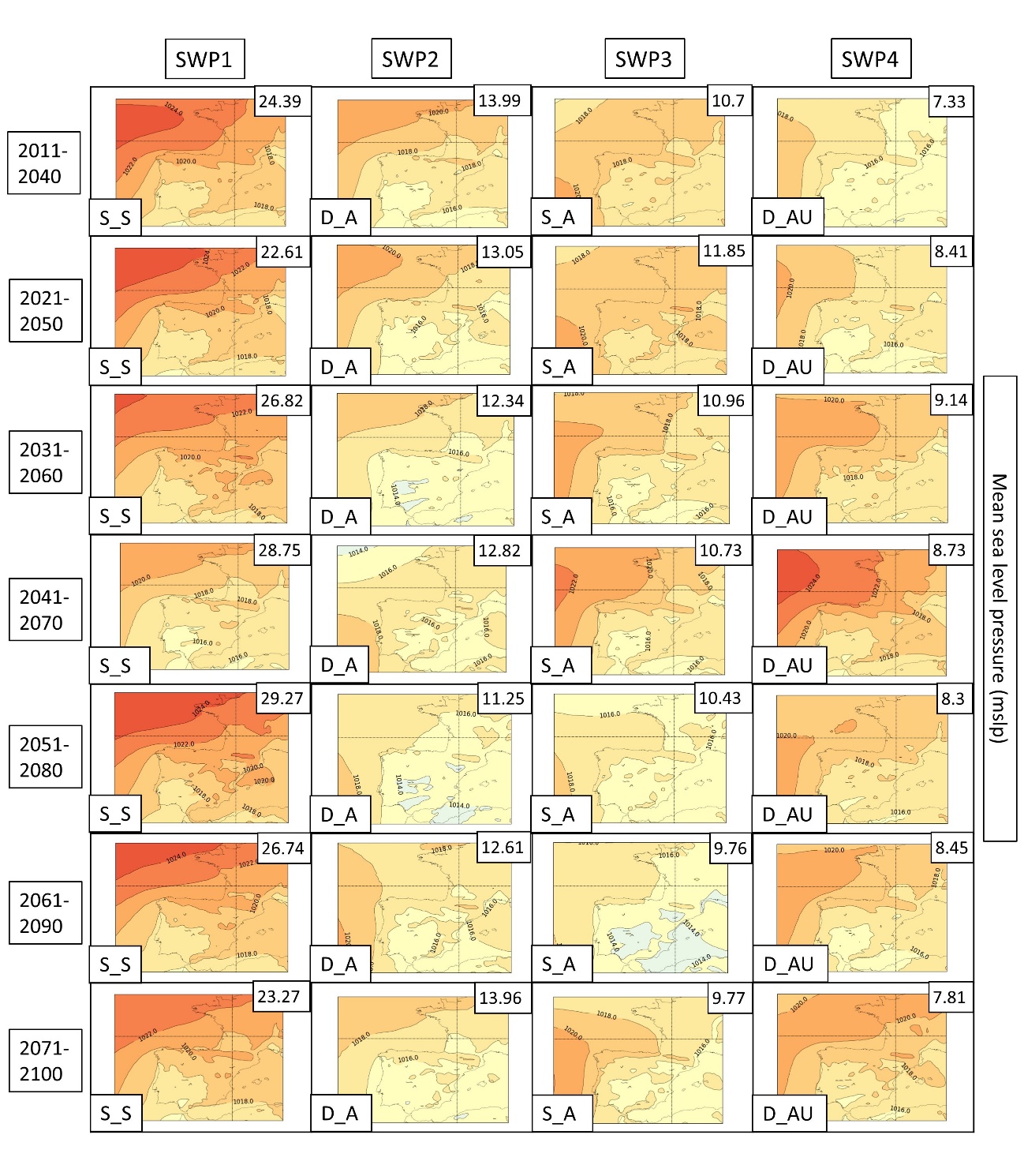


**S19. SWPs for CORDEX RCP4.5 scenario.** **Geopotential height at 500 hPa (Z500) variable (in m). Complete figure with periods 2011-2100. Variance explained in the upper-right corner and SWP in the bottom-left. Model: REMO.**


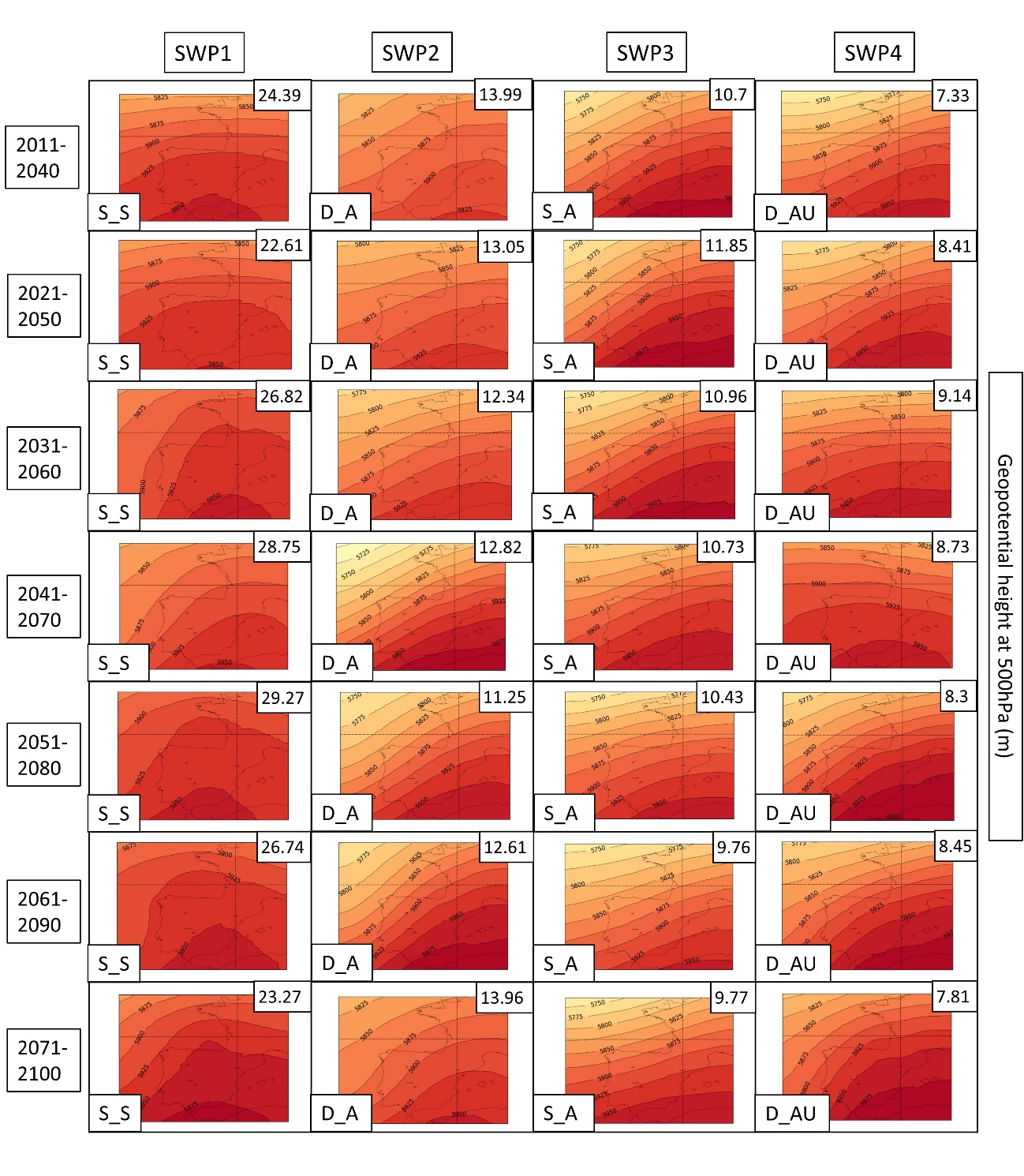


**S20. SWPs for CORDEX RCP4.5 scenario.** **Daily maximum temperature at 2m variable (in °C).** **Complete figure with periods 2011-2100. Variance explained in the upper-right corner and SWP in the bottom-left. Model: REMO.**


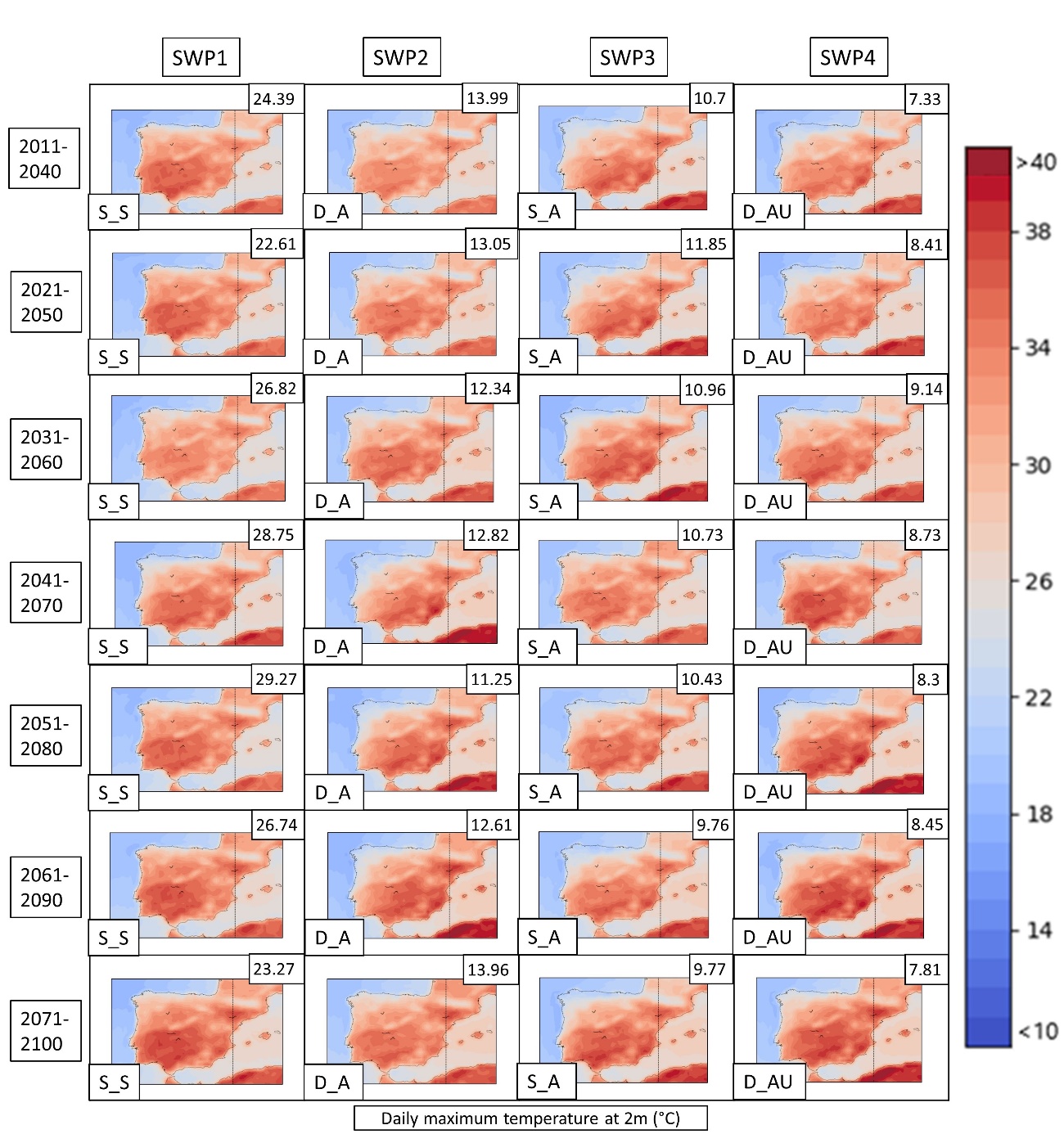


**S21. SWPs for CORDEX RCP4.5 scenario. MSLP variable (in hPa) is represented. Complete figure with periods 2011-2100. Variance explained in the upper-right corner and SWP in the bottom-left. Model: HIRHAM.**


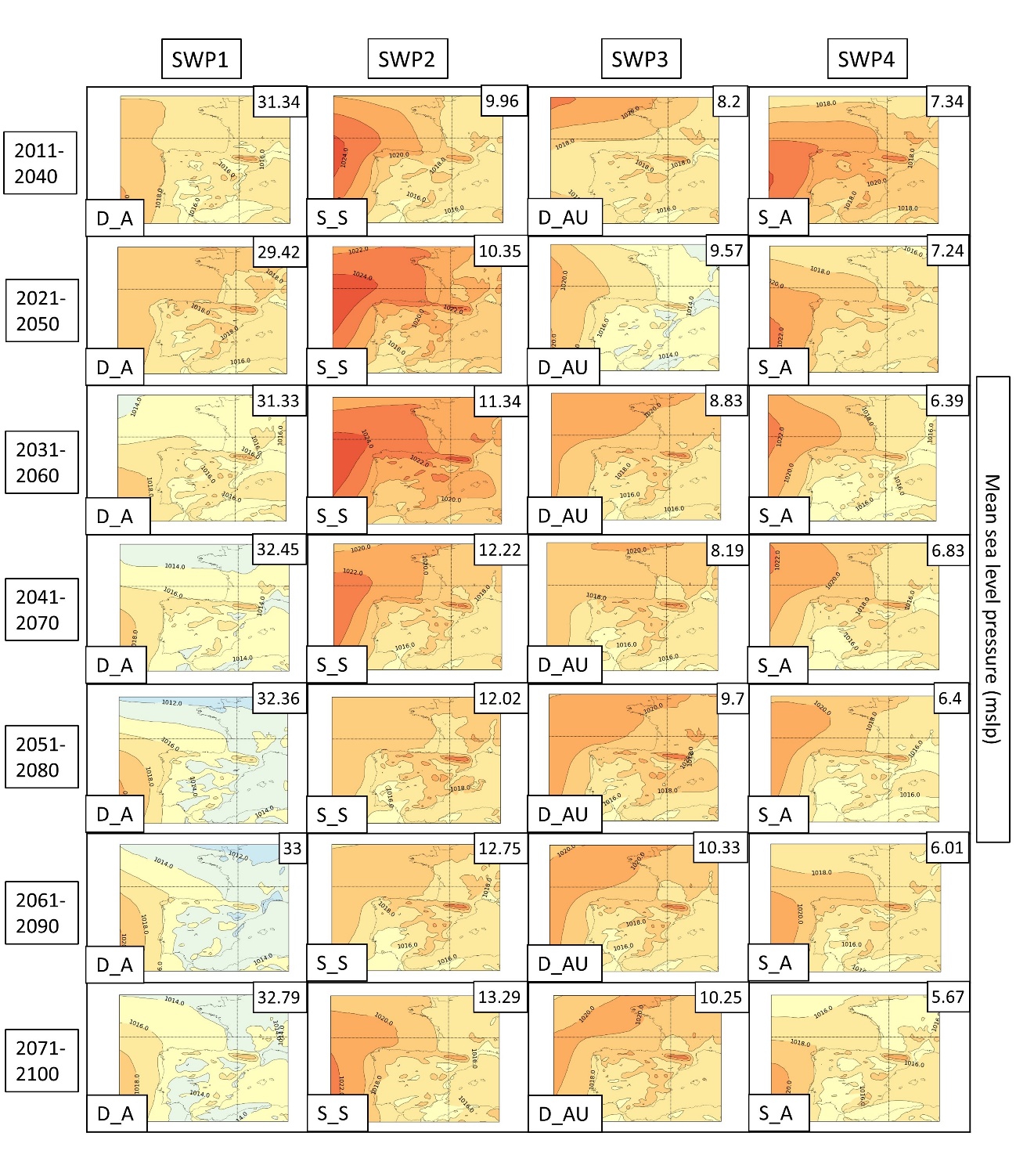


**S22. SWPs for CORDEX RCP4.5 scenario.** **Geopotential height at 500 hPa (Z500) variable (in m). Complete figure with periods 2011-2100. Variance explained in the upper-right corner and SWP in the bottom-left. Model: HIRHAM.**


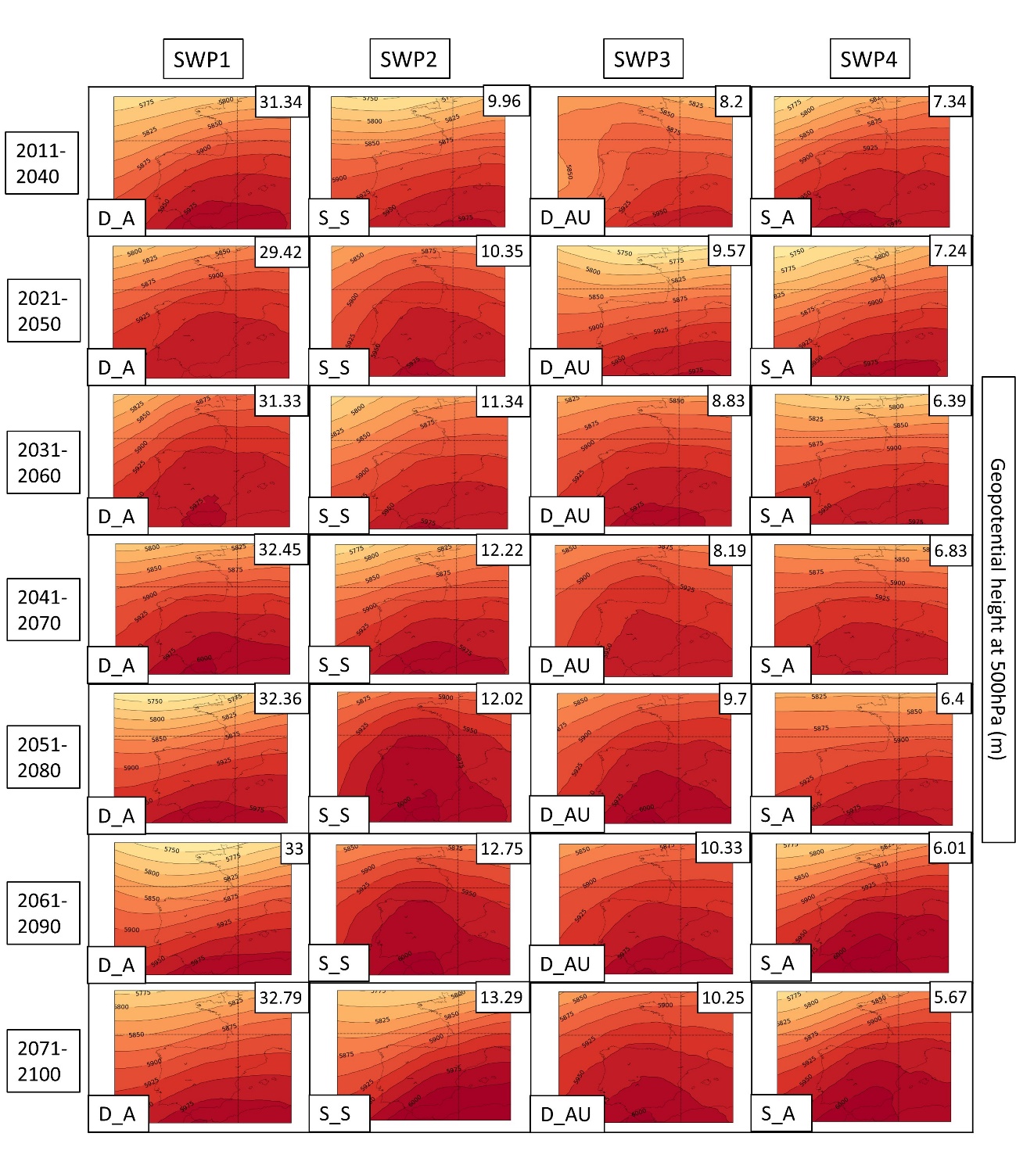


**S23. SWPs for CORDEX RCP4.5 scenario.** **Daily maximum temperature at 2m variable (in °C).** **Complete figure with periods 2011-2100. Variance explained in the upper-right corner and SWP in the bottom-left. Model: HIRHAM.**


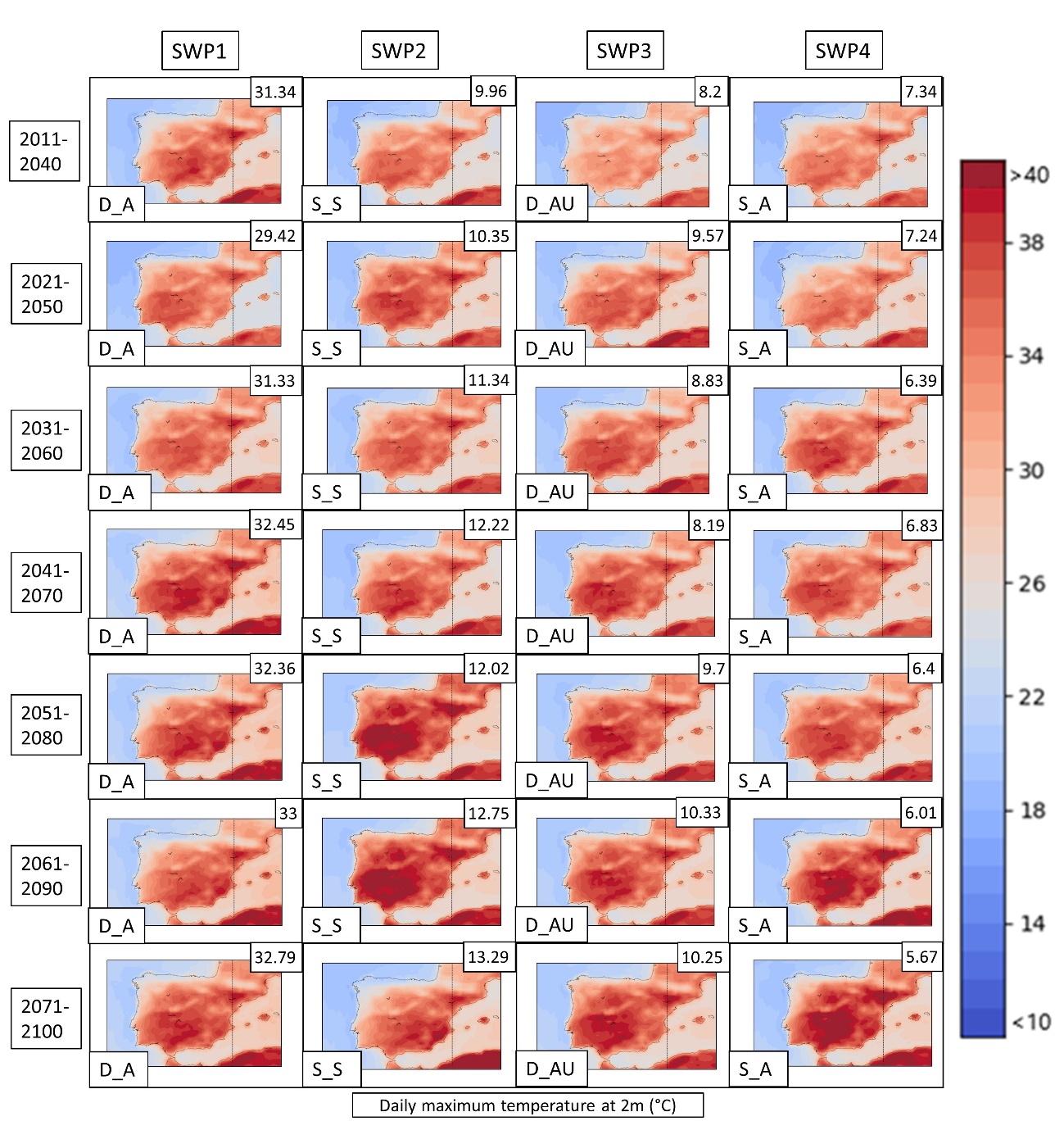


**S24: CORDEX 8.5 description**

**SWP-S_S: stationary and stable pattern.** According to WRF and HIRHAM, the MSLP for the RCP8.5 shows deeper thermal lows located over the IP and the Mediterranean Sea. REMO 8.5 is consistent with RCP4.5 and simulates a blocking anticyclone which reduces the presence of intense thermal lows. Z500 shows similar patterns in comparison with RCP4.5. WRF is consistent and shows an anticyclonic ridge generating west-flow. REMO projects more dynamism in the beginning of the period but, in the end, simulates an intense anticyclonic ridge, similar to HIRHAM, with values that reach more than 6050 m.

**SWP-S_A: stationary and advective.** MSLP shows consistency in all three models in comparison to RCP4.5, although WRF and HIRHAM show more intense thermal lows over the Mediterranean Sea. Z500 shows a more intense anticyclonic ridge in all three models in comparison with the RCP4.5. The north shift of the ridge also generates less advection in the IP. The geopotential height reaches more than 6000 m in HIRHAM and REMO.

**SWP-D_AU: dynamical, advective and undulated.** WRF and HIRHAM show an increase of the intensity of thermal lows in the MSLP in comparison to RCP4.5, which are mostly located over the Mediterranean region. Z500 shows less undulated and more stable structures due to the north shift of the anticyclonic ridge. The geopotential height values reach 6050 in HIRHAM and REMO.

**SWP-D_A: dynamic and advective.** MSLP shows an increase of blocking anticyclones and no important changes in the intensity of thermal lows. Otherwise, Z500 shows the same trend in all three models, which is the north shift displacement of the anticyclonic ridge, generating more stability in the IP and a less advective situation.

**S25. SWPs for CORDEX RCP8.5 scenario. MSLP variable (in hPa) is represented. Complete figure with periods 2011-2100. Variance explained in the upper-right corner and SWPin the bottom-left. Model: WRF.**


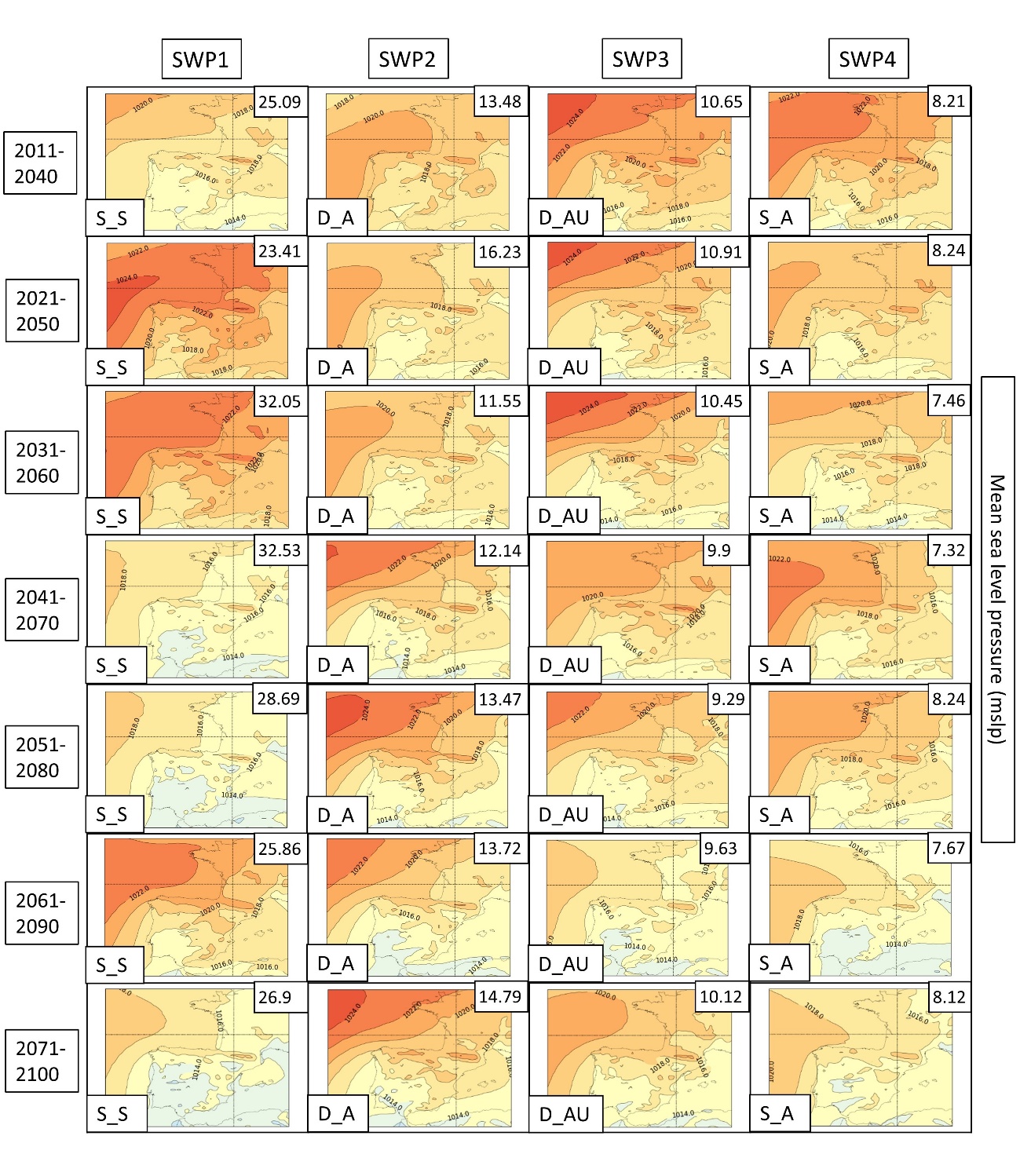


**S26. SWPs for CORDEX RCP8.5 scenario.** **Geopotential height at 500 hPa (Z500) variable (in m). Complete figure with periods 2011-2100. Variance explained in the upper-right corner and SWP in the bottom-left. Model: WRF.**


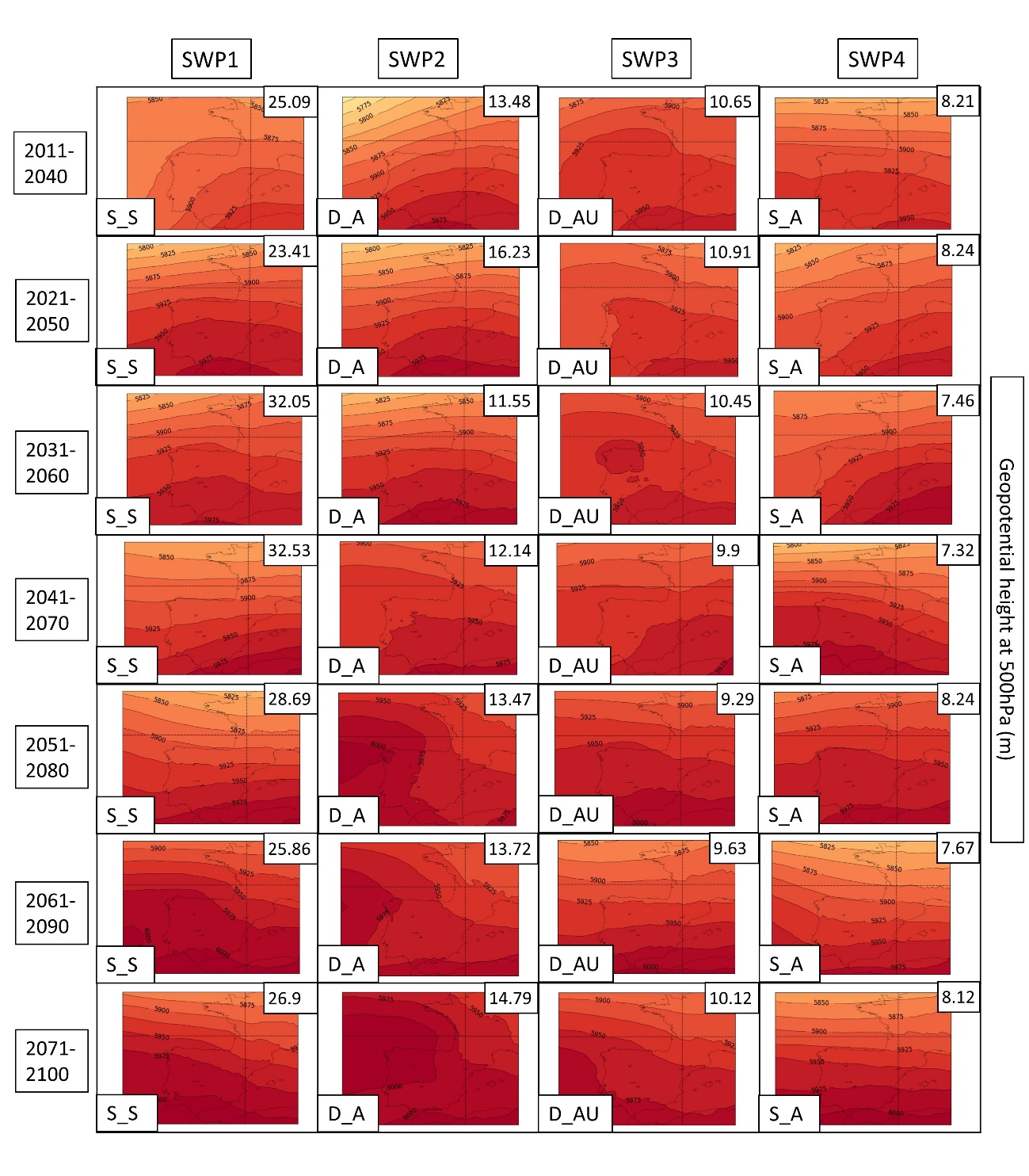


**S27. SWPs for CORDEX RCP8.5 scenario.** **Daily maximum temperature at 2m variable (in °C).** **Complete figure with periods 2011-2100. Variance explained in the upper-right corner and SWP in the bottom-left. Model: WRF.**


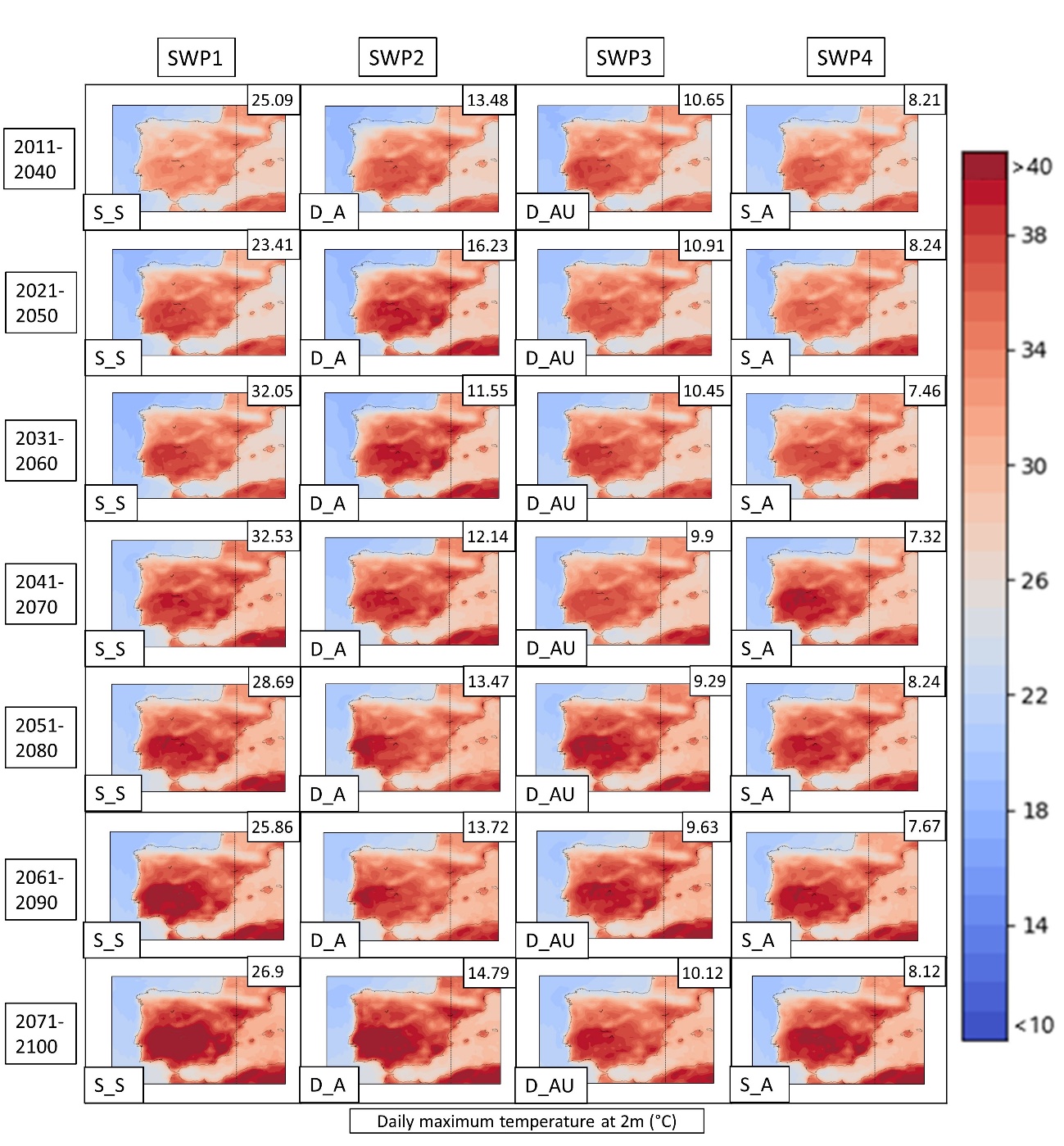


**S28. SWPs for CORDEX RCP8.5 scenario. MSLP variable (in hPa) is represented. Complete figure with periods 2011-2100. Variance explained in the upper-right corner and SWP in the bottom-left. Model: REMO**


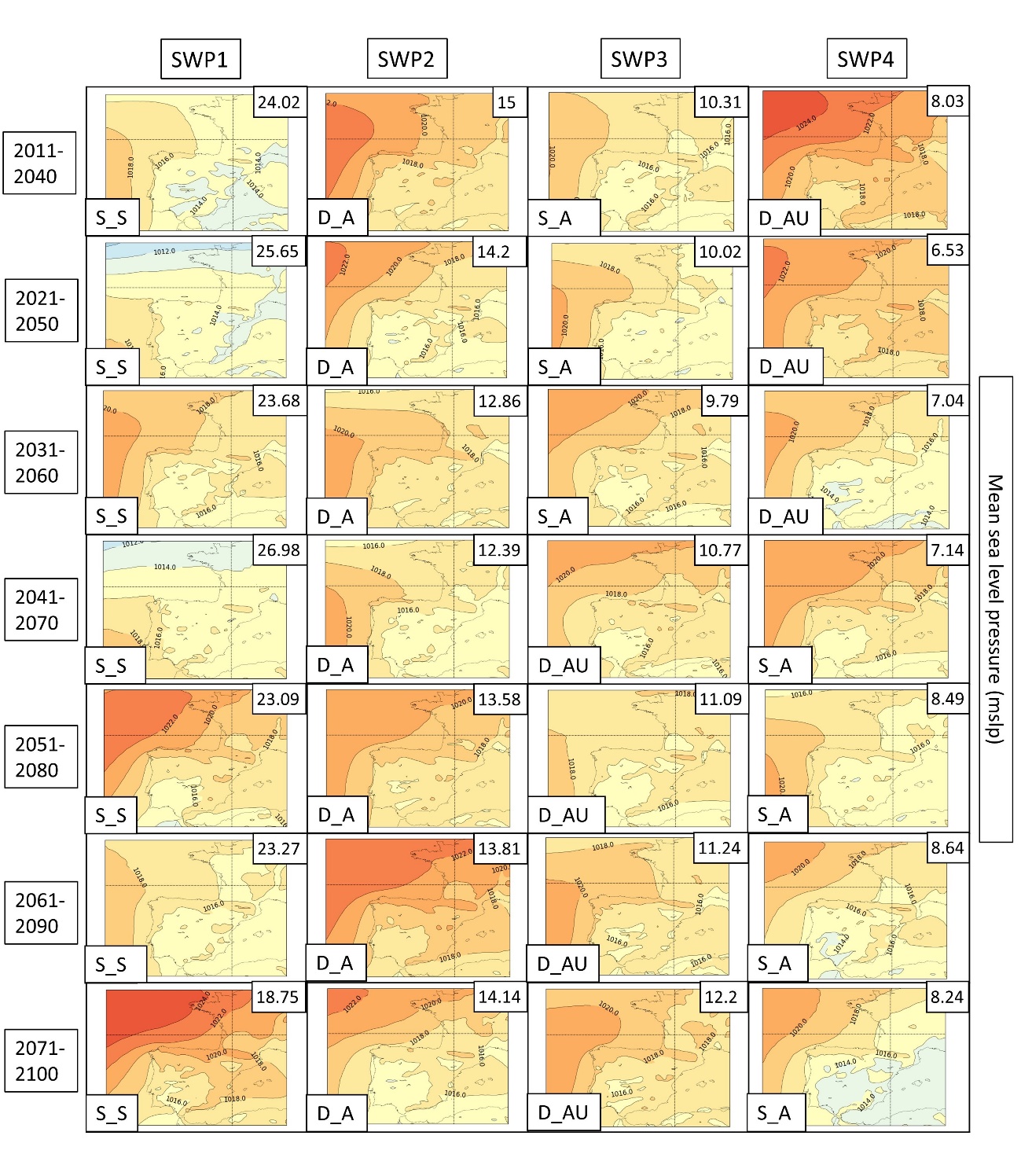


**S29. SWPs for CORDEX RCP8.5 scenario.** **Geopotential height at 500 hPa (Z500) variable (in m). Complete figure with periods 2011-2100. Variance explained in the upper-right corner and SWP in the bottom-left. Model: REMO.**


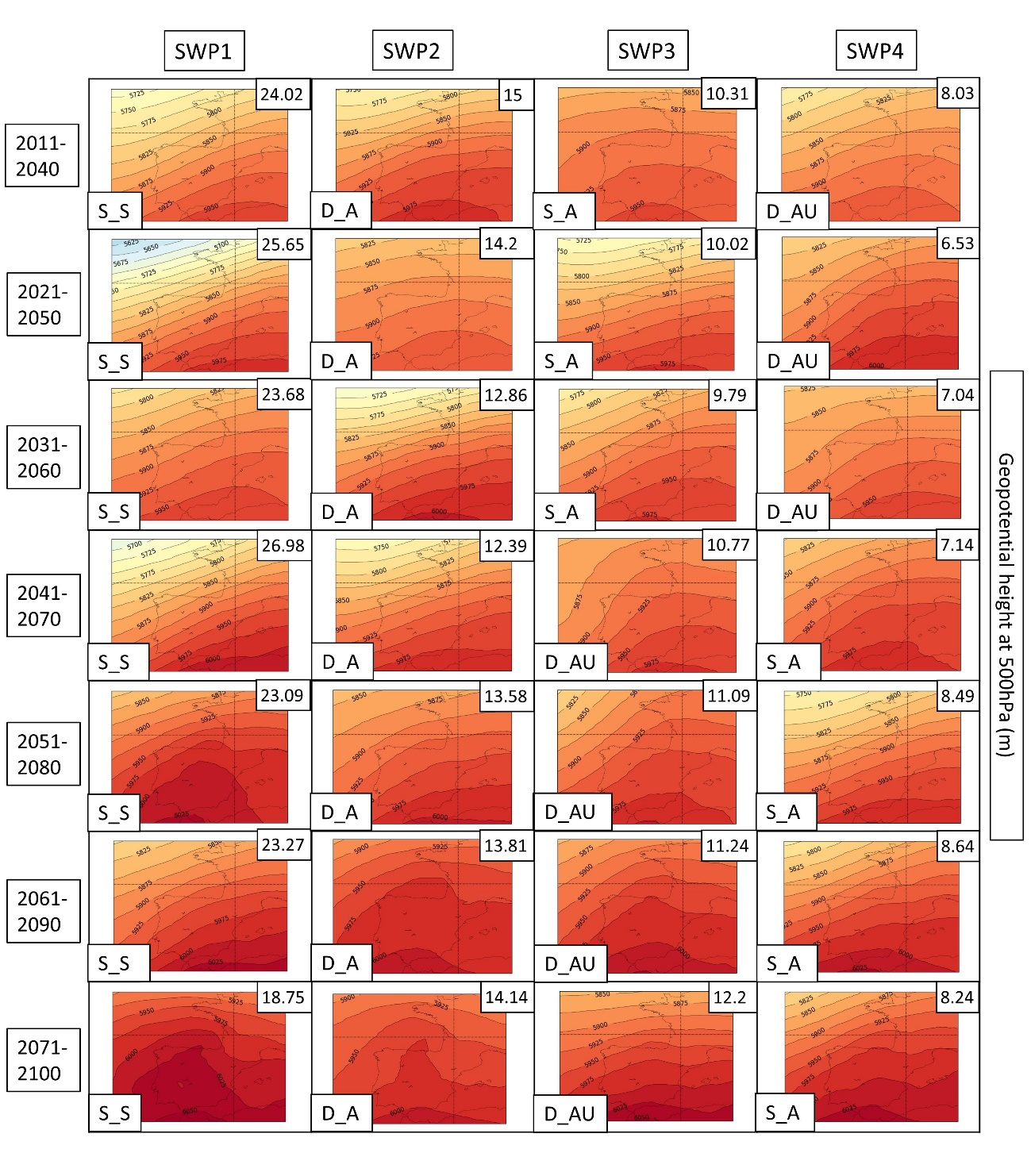


**S30. SWPs for CORDEX RCP8.5 scenario.** **Daily maximum temperature at 2m variable (in °C).** **Complete figure with periods 2011-2100. Variance explained in the upper-right corner and SWP in the bottom-left. Model: REMO.**


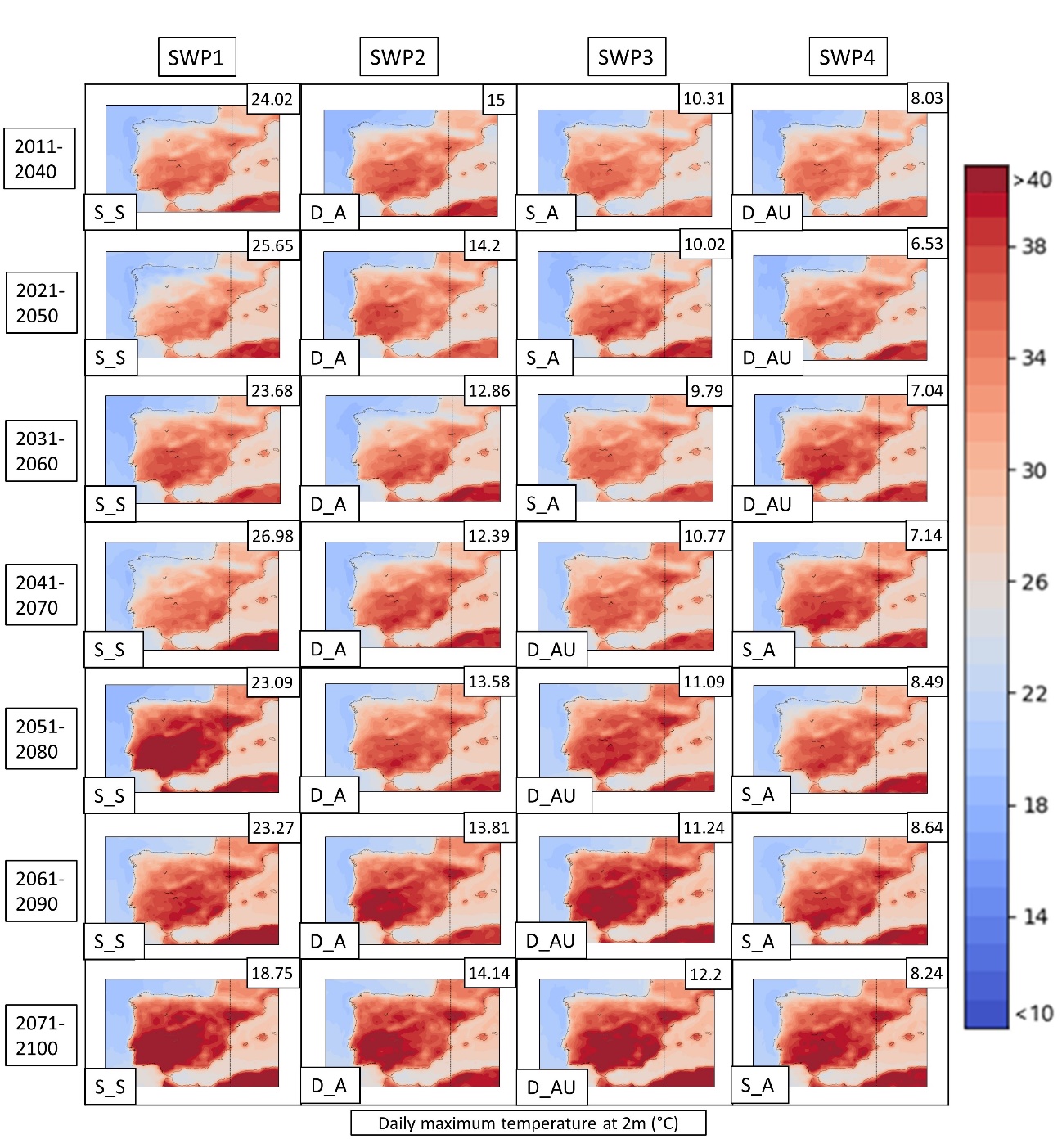


**S31. SWPs for CORDEX RCP8.5 scenario. MSLP variable (in hPa) is represented. Complete figure with periods 2011-2100. Variance explained in the upper-right corner and SWP in the bottom-left. Model: HIRHAM.**


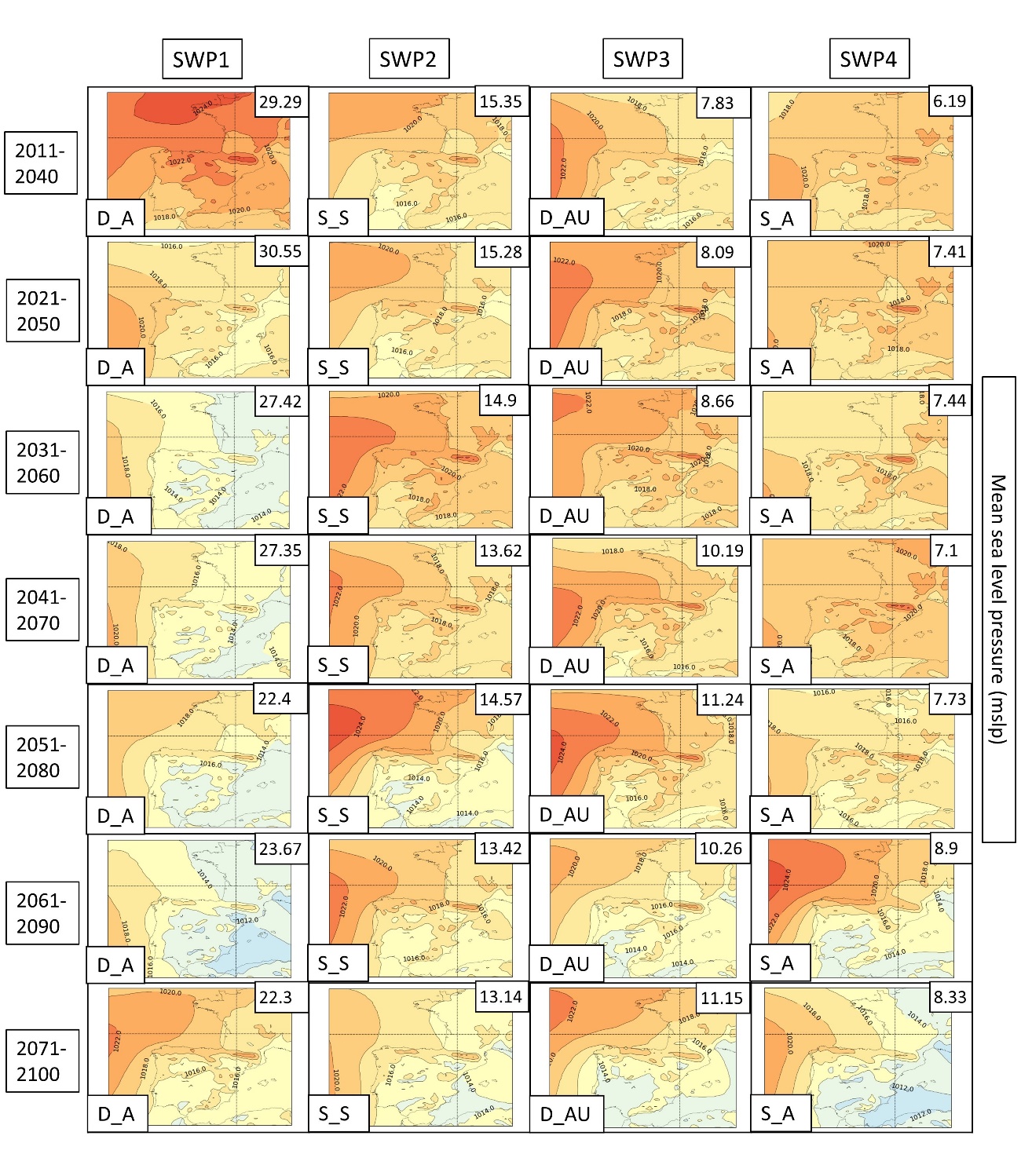


**S32. SWPs for CORDEX RCP8.5 scenario.** **Geopotential height at 500 hPa (Z500) variable (in m). Complete figure with periods 2011-2100. Variance explained in the upper-right corner and SWP in the bottom-left. Model: HIRHAM.**


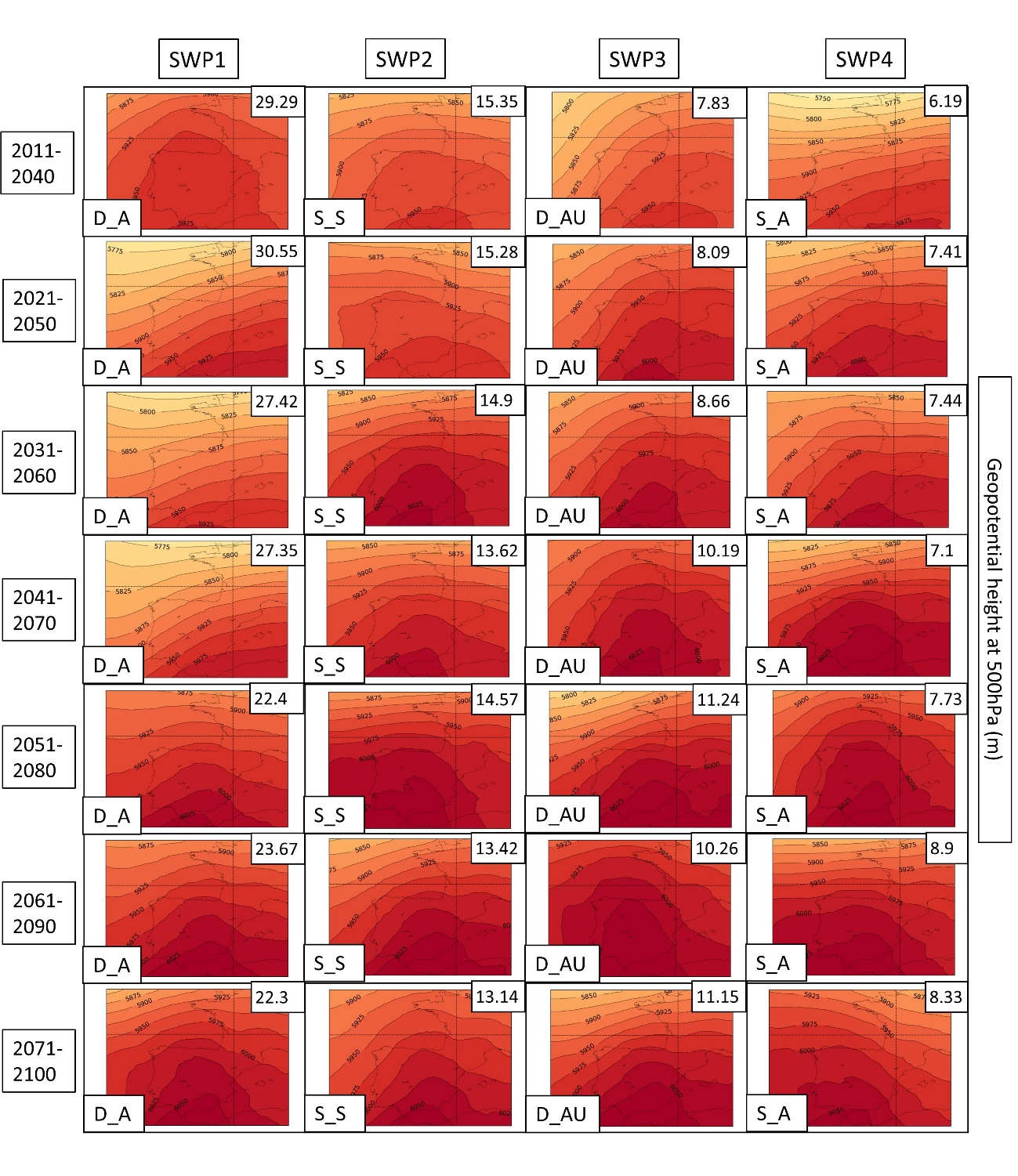


**S33. SWPs for CORDEX RCP8.5 scenario.** **Daily maximum temperature at 2m variable (in °C).** **Complete figure with periods 2011-2100. Variance explained in the upper-right corner and SWP in the bottom-left. Model: HIRHAM.**


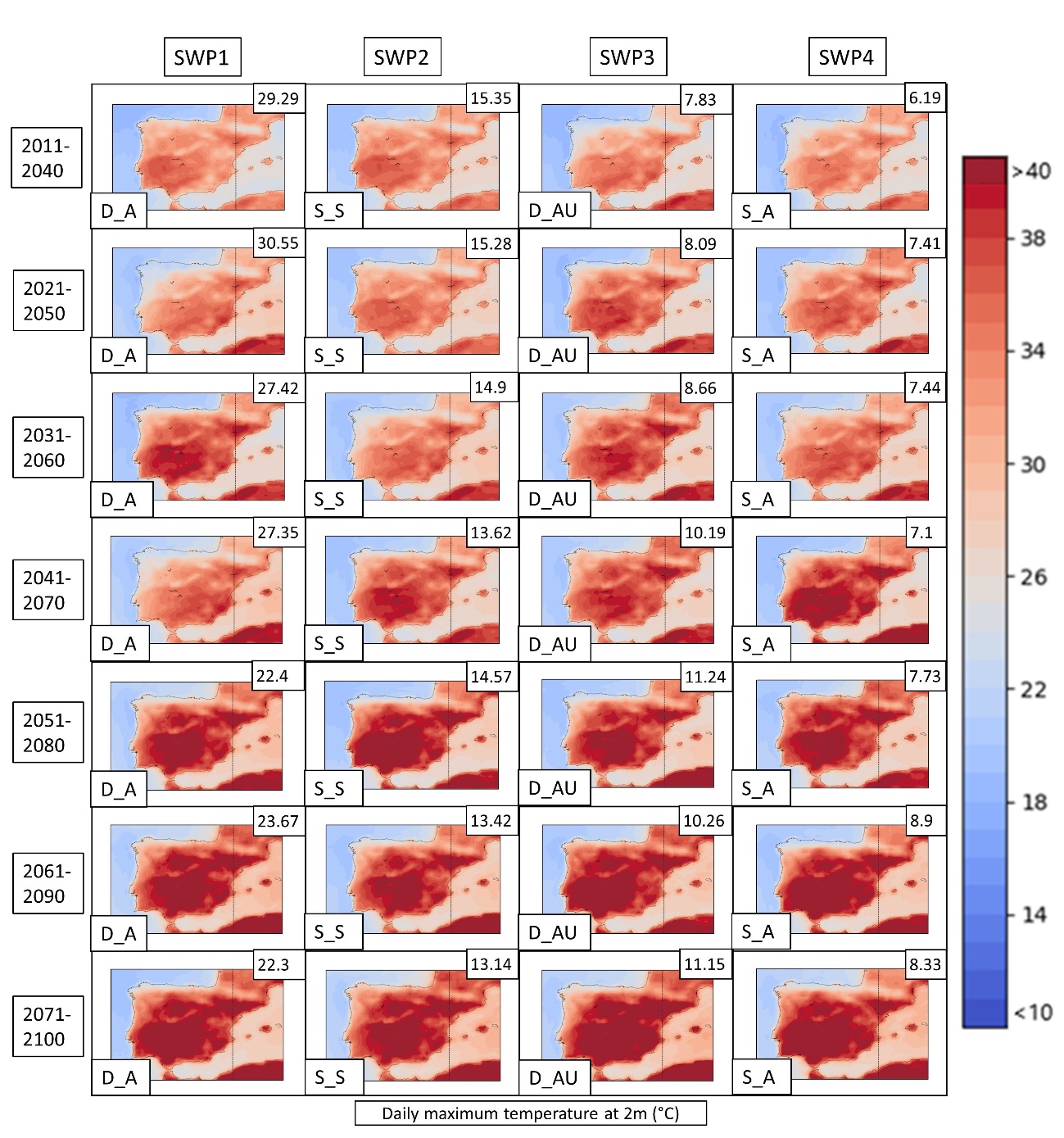

Supplement: Supplementary file 1 — Supplementary file1 (DOCX 12880 KB) [file 382_2023_6828_MOESM1_ESM.docx]
